# Dually Labeled Neurotensin NTS_1_R Ligands for Probing Radiochemical and Fluorescence-Based Binding Assays

**DOI:** 10.1021/acs.jmedchem.4c01470

**Published:** 2024-09-11

**Authors:** Fabian J. Ertl, Sergei Kopanchuk, Nicola C. Dijon, Santa Veikšina, Maris-Johanna Tahk, Tõnis Laasfeld, Franziska Schettler, Albert O. Gattor, Harald Hübner, Nataliya Archipowa, Johannes Köckenberger, Markus R. Heinrich, Peter Gmeiner, Roger J. Kutta, Nicholas D. Holliday, Ago Rinken, Max Keller

**Affiliations:** † Institute of Pharmacy, Faculty of Chemistry and Pharmacy, University of Regensburg, Universitätsstraβe 31, D-93053 Regensburg, Germany; ‡ Institute of Chemistry, University of Tartu, Ravila 14a, 50411 Tartu, Estonia; § School of Life Sciences, University of Nottingham, Queen’s Medical Centre, Nottingham NG7 2UH, U.K.; ∥ Department of Chemistry and Pharmacy, Medicinal Chemistry, Friedrich Alexander University, Nikolaus-Fiebiger-Straβe 10, D-91058 Erlangen, Germany; ⊥ Institute of Biophysics and Physical Biochemistry, Faculty of Biology and Preclinical Medicine, University of Regensburg, Universitätsstraβe 31, D-93053 Regensburg, Germany; # Institute of Physical and Theoretical Chemistry, Faculty of Chemistry and Pharmacy, University of Regensburg, Universitätsstraβe 31, D-93053 Regensburg, Germany

## Abstract

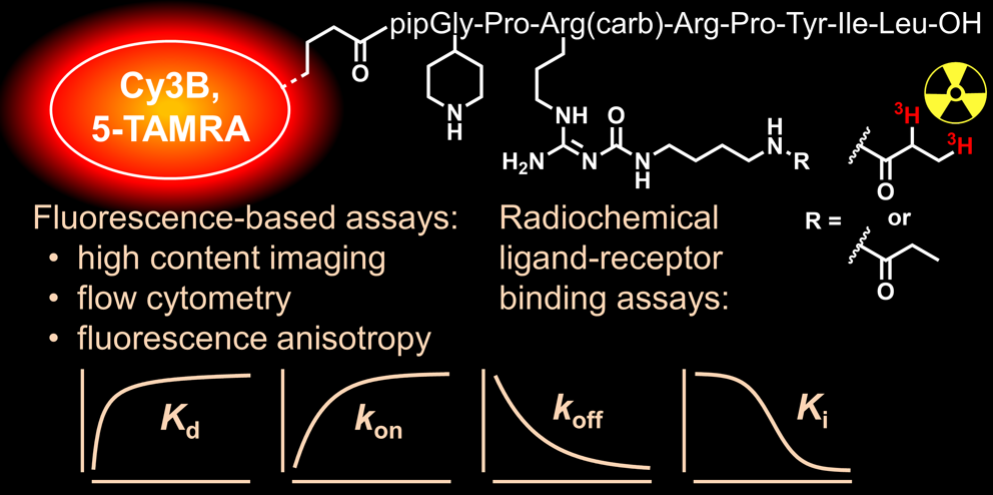

The determination of ligand–receptor binding affinities plays a key role in the development process of pharmaceuticals. While the classical radiochemical binding assay uses radioligands, fluorescence-based binding assays require fluorescent probes. Usually, radio- and fluorescence-labeled ligands are dissimilar in terms of structure and bioactivity, and can be used in either radiochemical or fluorescence-based assays. Aiming for a close comparison of both assay types, we synthesized tritiated fluorescent neurotensin receptor ligands ([^3^H]**13**, [^3^H]**18**) and their nontritiated analogues (**13**, **18**). The labeled probes were studied in radiochemical and fluorescence-based (high-content imaging, flow cytometry, fluorescence anisotropy) binding assays. Equilibrium saturation binding yielded well-comparable ligand–receptor affinities, indicating that all these setups can be used for the screening of new drugs. In contrast, discrepancies were found in the kinetic behavior of the probes, which can be attributed to technical differences of the methods and require further studies with respect to the elucidation of the underlying mechanisms.

## Introduction

The development of pharmacological tools and therapeutics acting at biological receptors involves the determination of ligand–receptor binding affinities. The standard method for studying ligand–receptor binding is the competition binding assay requiring a well characterized labeled receptor ligand. Half a century ago, binding affinities were nearly exclusively determined in radioligand competition binding assays, using a radiolabeled probe that is displaced from the receptor by the studied compound. Today, various luminescence-based methods, such as high-content imaging (HCI), flow cytometry (FC), fluorescence anisotropy (FA) measurements, and bioluminescence resonance energy transfer (BRET)-based binding assays are available to study ligand–receptor binding. The different techniques have advantages and disadvantages. The radiochemical binding assay is highly sensitive and advantageous with respect to the quantification of bound and free labeled ligand. Moreover, the labeling procedure (introduction of a radionuclide such as iodine-125 or tritium) has usually little impact on the ligand structure and consequently also on the bioactivity of the labeled probe. On the downside, radiochemical assays are associated with special safety precautions, the need for specialized laboratory permits, high costs (purchase of radioligands, radioactive waste disposal in the case of long-lived isotopes such as tritium) and the emergence of undefined ligand species in the radioligand stock due to radionuclide disintegration. Furthermore, the radiochemical binding assay requires a separation of unbound and bound radioligand after sample incubation precluding a measurement under equilibrium conditions. Most disadvantages associated with radiochemical assays turn into advantages when ligand–receptor binding is studied by fluorescence-based methods: several techniques (e.g., FC, FA-, and BRET-based assays) enable a measurement of bound ligand under homogeneous conditions, the potential health risk is very low, and waste disposal is inexpensive. Moreover, several luminescence-based methods allow a fast and automated sample measurement by multimode plate readers (e.g., HCI, FA, BRET). However, there are also drawbacks connected with fluorescence-based binding assays. The attachment of a bulky fluorescent dye to a ligand likely affects receptor binding affinity, and the hydrophobic nature of the fluorescent dye usually results in unfavorable physicochemical properties of the fluorescent ligand potentially causing poor solubility and higher unspecific binding. Fluorescence properties such as quantum yield and spectral characteristics depend on many factors, such as the chemical or biological environment (solvent polarity, buffer supplements, cellular proteins and lipids), the concentration of fluorescence quenchers (e.g., molecular oxygen), pH and temperature, impeding a quantification of the concentration of bound and unbound fluorescent ligand.^1^ Moreover, fluorophores can be prone to photobleaching and might catalyze photoreactions.^2−5^

**Figure 1:**
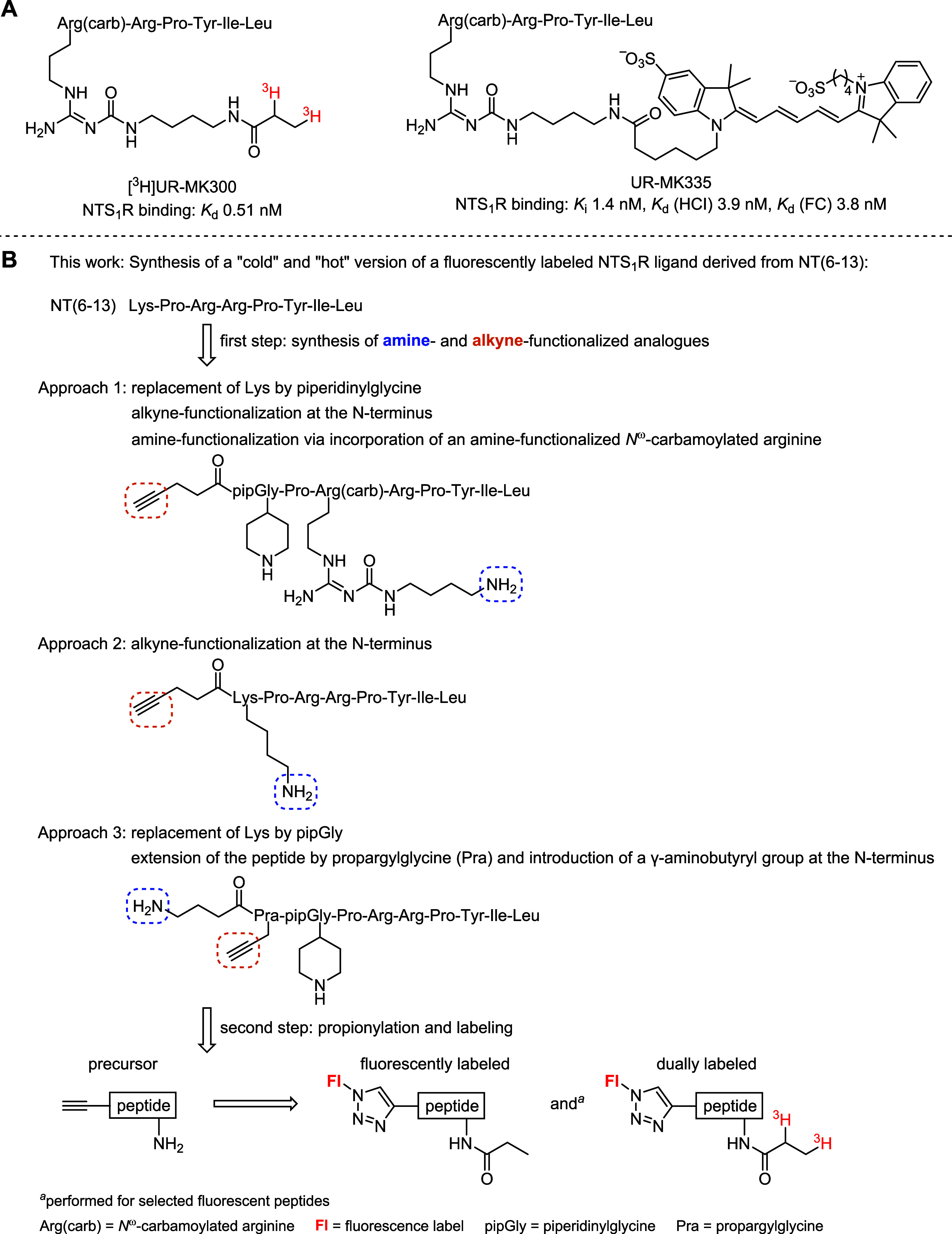
(A) Structures and NTS_1_R binding affinities of the reported tritium-labeled NT(8–13) derivative [^3^H]UR-MK300^13^ and the sulfo-Cy5 labeled ligand UR-MK335^16^ both bearing the label at an *N*^ω^-carbamoylated arginine. (B) Concept of the present study: design and synthesis of fluorescently labeled NT(6–13) derivatives and their structurally identical tritium-labeled analogues as molecular tools to be studied in fluorescence-based and radiochemical binding assays. Three different approaches were pursued. Note that the tritium atoms in the [^3^H]propionyl residues do not represent a quantity of tritium isotopes; they only indicate that tritium is present in the respective position.

Due to the different kind of labels, radioligands and fluorescent ligands are, even if derived from the same pharmacophore or ligand, structurally different, and their receptor binding characteristics cannot be studied in the same type of binding assay. This means that radiochemical and fluorescence-based binding assays usually cannot be directly compared based on the labeled ligands. They can merely be compared indirectly by the determination of ligand–receptor binding affinities of unlabeled ligands in competition binding assays. To enable a direct comparison of radiochemical and fluorescence-based binding assays, we synthesized tritium labeled fluorescent ligands and their structurally identical “cold”/nonradioactive analogues, which were investigated in both, radiochemical and fluorescence-based binding assays, respectively. The neurotensin (NT) receptor type 1 (NTS_1_R),^6^ for which peptidic fluorescent and tritiated ligands were already reported (examples shown in Figure 1A),^7−17^ was chosen as model system.

Aiming for dually labeled ligands, a series of NT(6–13) derivatives, carrying two orthogonal functionalities (alkyne, amine), were synthesized followed by propionylation at the amino group and fluorescence labeling via the alkyne functionality to obtain the “cold” fluorescent ligands (Figure 1B) (note: the propionyl moiety was chosen since succinimidyl [^3^H]propionate is a commercially available labeling reagent allowing for an introduction of [^3^H]propionyl groups). For two selected fluorescent ligands, the tritiated analogues were prepared by introducing a [^3^H]propionyl moiety instead of the propionyl group (Figure 1). While the “cold” fluorescent ligands were characterized by saturation, kinetic, and competition binding studies in three different fluorescence-based assays (high-content imaging (HCI), flow cytometry (FC), fluorescence anisotropy (FA); comprehensive reviews about these methods have been published^18−22^), the dually labeled peptides were investigated in radiochemical saturation, kinetic, and competition binding assays. To note, for the benefit of a better comparison, all binding studies were performed using wild-type hNTS_1_R, i.e., genetically modified proteins such as hNTS_1_R fused to nanoluciferase (useful for BRET binding assays)^23^ were not involved. Likewise, the same type of buffer was used for all binding studies.

## Results and Discussion

### Ligand Design

As lead compound, NT(6–13) (cf. Figure 1B) was chosen since crystal structures of the NTS_1_R in complex with NT analogues revealed that the NTS_1_R binding site is occupied by the C-terminal hexapeptide sequence NT(8–13).^24−26^ An N-terminal extension of the NT(8–13) peptide sequence does not substantially effect NTS_1_R binding as exemplified by a reported study demonstrating almost equal NTS_1_R affinities for full length neurotensin, NT(6–13), and NT(8–13).^27^ Therefore, Lys^6^ and Pro^7^ in NT(6–13) can serve as a spacer between the N-terminus of NT(8–13) and a bulky, N-terminally introduced substituent as confirmed by NT(6–13)-derived PET ligands showing high NTS_1_R affinity.^12,28^ It should be noted that structural modifications at the C-terminus were not considered because this would have high impact on NTS_1_R binding affinity due to the localization of the C-terminus of NT(8–13) in the deep part of the binding pocket.^24^

For the majority of the fluorescently labeled peptides, the bulky fluorescent dye and the propionyl group were attached N-terminally and at an amino acid side chain, respectively. Thus, the introduction of a propionyl or tritiated propionyl moiety required an amino-functionalized side chain, and conjugation to the fluorescent dye via click chemistry required an alkyne-functionalized N-terminus. According to approach 1 (cf. Figure 1B), the primary amino group was introduced by incorporation of an amino-functionalized *N*^ω^-carbamoylated arginine derived from **1**.^13^ To avoid the presence of two side chains with a primary amino group in these peptides, Lys^6^ was replaced by piperidinylglycine (pipGly) as a lysine mimic, reported to act as a bioisoster of lysine.^12,28,29^ To note, for steric reasons, the primary amino group of the amino-functionalized *N*^ω^-carbamoylated arginine is preferred over the secondary amino group in pipGly by acylating reagents, allowing a selective propionylation of the former. In the case of NT(6–13) derivatives containing lysine or d-lysine, the amino-functionalized side chain for propionylation is provided by lysine (approach 2, Figure 1B). For approaches 1 and 2, the alkyne moiety was introduced by N-terminal acylation of the peptides with 4-pentynoic acid.

Regarding approach 3 (cf. Figure 1B), Lys^6^ is replaced by pipGly and the peptide is N-terminally extended by propargylglycine (Pra), which carries an γ-aminobutyryl residue at the *N*^α^-nitrogen atom, meaning that the fluorescent dye is attached to the side chain of Pra and the propionyl group is introduced N-terminally.

**Scheme 1:**
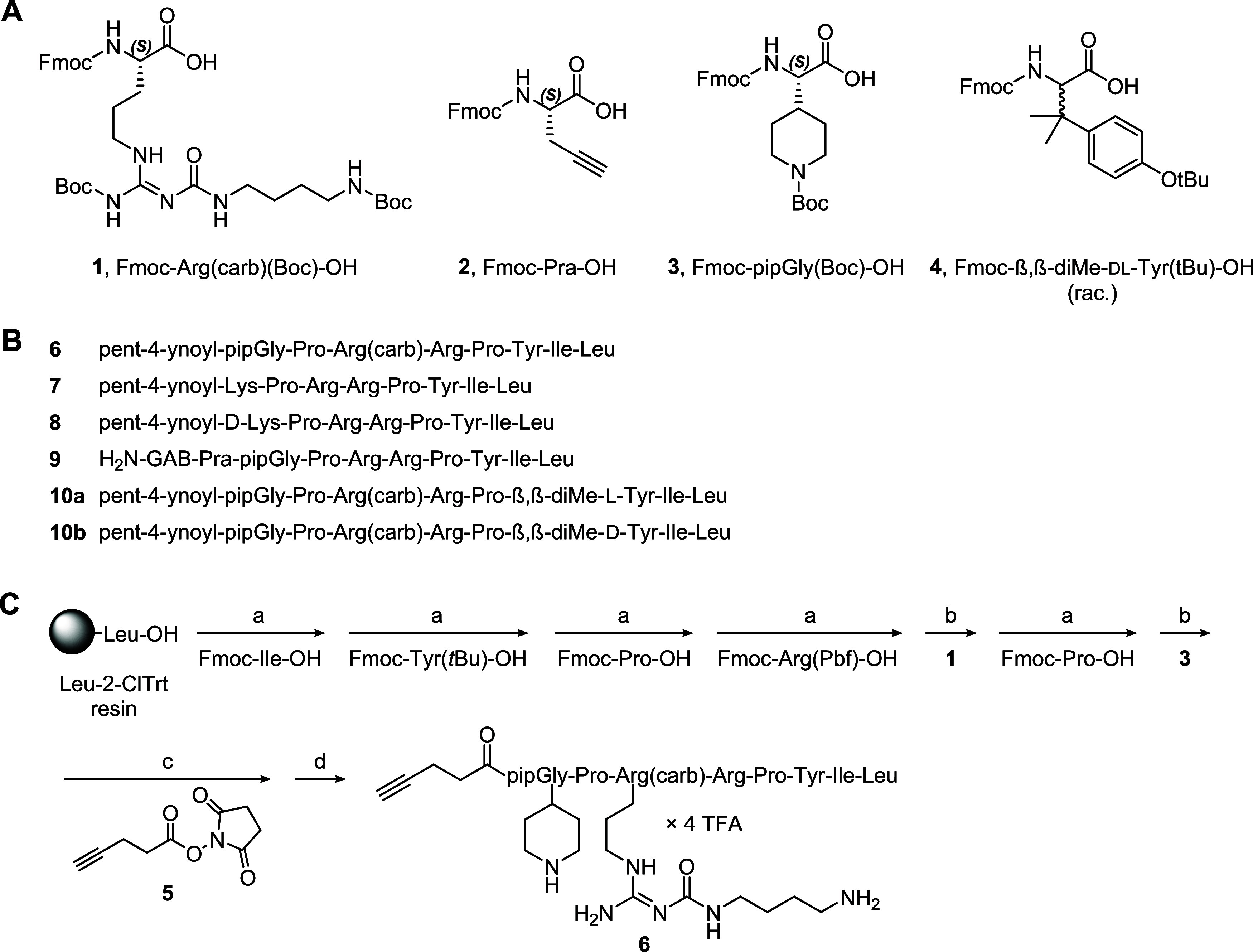
Synthesis of Amine- and Alkyne-Functionalized Precursor Peptides (A) Structures of used unnatural amino acids. (B) Amino acid sequences of the amine- and alkyne-functionalized precursor peptides **6**–**9**, **10a**, and **10b**. GAB = γ-aminobutyryl. Overall yields for SPPS: 10–38%. (C) SPPS exemplarily shown for peptide **6**. Reagents and conditions: (a) amino acid coupling: Fmoc-amino acid/HOBt/HBTU/DIPEA (5/5/4.9/10 equiv), DMF/NMP 8:2 v/v, 35 °C, 2 × 45 min (“double” coupling), Fmoc deprotection: 20% piperidine in DMF/NMP 8:2 v/v, rt, 2 × 10 min; (b) **1** or **3**/HOBt/HBTU/DIPEA (3/3/2.95/6 equiv), DMF/NMP 8:2 v/v, 35 °C, 16 h (“single” coupling), Fmoc deprotection: as under (a); (c) **5**/DIPEA (10/10 equiv), DMF/NMP 8:2 v/v, 35 °C, 30 min; (d) (1) TFA/CH_2_Cl_2_ 1:3 v/v, rt, 2 × 20 min; (2) TFA/H_2_O 95:5 v/v, rt, 5 h.

The rhodamine-type dye 5-TAMRA and the indolinium-type cyanine dye Cy3B (cf. Scheme 1), both exhibiting a high extinction coefficient, high fluorescence quantum yield, and a fluorescence lifetime well compatible with fluorescence polarization-based binding assays,^5,21,30^ were used as fluorescence label.

### Synthesis

Using Fmoc-protected derivatives of proteinogenic amino acids (Arg, Ile, Leu, Lys, Pro) and unnatural amino acids (**1**–**4**, see Scheme 1A), the amine- and alkyne-functionalized precursor peptides **6**–**9**, **10a**, and **10b** (sequences shown in Scheme 1B) were synthesized by solid-phase peptide synthesis (SPPS). To exemplify the peptide synthesis, the preparation of **6**, containing an amino-functionalized *N*^ω^-carbamoylated arginine derived from **1**, is shown in Scheme 1C. In **6**–**8**, **10a**, and **10b**, the alkyne group was introduced N-terminally by final treatment of the resin-bound, Fmoc-deprotected peptide with succinimidyl ester **5**.

In peptide **9**, the alkyne moiety was introduced by incorporation of Pra in the N-terminal position. To introduce an amino-functionalized spacer, this peptide was N-terminally acylated with γ-aminobutyric acid. Worth mentioning, SPPS of **9** (exact mass: 1233.7346 Da) yielded a side product (mass identified by HRMS: 1310.9922 Da) in approximately equal amount to **9**, supposedly caused by the nonprotected alkyne group in Fmoc-Pra–OH (**2**). Separation of the side product from **9** by preparative reversed-phase high-performance liquid chromatography (RP-HPLC) was laborious due to similar retention times. Peptides **10a** and **10b** represent congeners of **6**, containing, compared to **6**, β,β-dimethyl-l-tyrosine (**10a**) or β,β-dimethyl-d-tyrosine (**10b**) instead of l-tyrosine. This modification was taken into consideration due to a recent study on NT(8–13)-derived PET ligands where the replacement of tyrosine by β,β-dimethyl-l-tyrosine resulted in analogues with high in vitro (blood plasma) and high in vivo stability, and increased NTS_1_R affinity.^31^ As Fmoc-β,β-dimethyl-dl-Tyr(tBu)–OH (**4**) was commercially available only as racemic mixture and used as such for peptide synthesis, two diastereomers (**10a** and **10b**) were obtained, which could be separated by preparative RP-HPLC. The assignment of the absolute configuration to β,β-dimethyl-tyrosine in **10a** and **10b** was based on reported, structurally closely related NT(8–13) derivatives, also containing β,β-dimethyl-l-tyrosine or β,β-dimethyl-d-tyrosine in position 11 (structures shown in Figure S1A, Supporting Information).^31^ The absolute configuration at the α-carbon of the β,β-dimethylated tyrosine in these peptides was determined based on CD spectroscopy involving an all-l-configured reference peptide.^31^ The assignment of the configuration to the β,β-dimethylated tyrosine in **10a** and **10b** was guided by, first, the elution order in RP-HPLC, and second, the NTS_1_R binding affinity. In the reported study, the peptide containing β,β-dimethyl-l-tyrosine eluted first and showed considerably higher NTS_1_R binding affinity compared to the diastereomer containing β,β-dimethyl-d-tyrosine (*K*_i_: 0.14 vs 56 nM).^31^ Since **10a** eluted before **10b** in RP-HPLC (same stationary phase and eluent as used in the reported study) and exhibits markedly higher NTS_1_R affinity than **10b** (Table 1), it represents the diastereomer with β,β-dimethyl-l-tyrosine and, consequently, **10b** contains β,β-dimethyl-d-tyrosine. The lower NTS_1_R affinity of the diastereomer containing the d-configured dimethylated tyrosine is also in agreement with a reported d-amino acid scan in NT(8–13), showing that inversion of the stereochemistry in positions 10–13 of NT(8–13) results in a marked decrease in NTS_1_R binding.^32^

**Table 1:** NTS_1_R and NTS_2_R Binding Affinities and NTS_1_R Agonistic Potencies

	NTS_1_R				NTS_2_R	
compd.	p*K*_i_ ± SEM^a^	*K*_i_ [nM]^a^	pEC_50_ ± SEM^b^	EC_50_ [nM]^b^	p*K*_i_ ± SEM^c^	*K*_i_ [nM]^c^
NT(8–13)	9.85^d^	0.14^d^	11.04 ± 0.1	0.0091	9.23 ± 0.06	0.62
**6**	9.22 ± 0.3	0.64	11.19 ± 0.07	0.0065	n.d.	n.d.
**7**	9.27 ± 0.04	0.54	n.d.	n.d.	n.d.	n.d.
**8**	9.08 ± 0.05	0.85	n.d.	n.d.	n.d.	n.d.
**9**	9.52 ± 0.2	0.33	n.d.	n.d.	n.d.	n.d.
**10a**	8.97 ± 0.01	1.1	9.50 ± 0.10	0.32	n.d.	n.d.
**10b**	6.87 ± 0.05	140	n.d.	n.d.	n.d.	n.d.
**13**	9.05 ± 0.2	1.1	10.30 ± 0.2	0.050	8.62 ± 0.2	2.4
**14**	8.86 ± 0.09	1.5	n.d.	n.d.	n.d.	n.d.
**15**	8.53 ± 0.06	3.0	n.d.	n.d.	n.d.	n.d.
**16**	9.14 ± 0.08	0.77	n.d.	n.d.	n.d.	n.d.
**18**	8.39 ± 0.07	4.2	9.41 ± 0.2	0.39	8.71 ± 0.1	1.9

a Determined by radioligand competition binding with [^3^H]UR-MK300^13^ at HT-29 colon carcinoma cells.

b Determined in a Fura-2 Ca^2+^ assay at CHO-NTS_1_R cells.

c Determined by competition binding with [^3^H]UR-MK300^13^ at homogenates of HEK293T-hNTS_2_R cells.

d Keller et al. (reported *K*_i_ value of 0.14 nM was converted to p*K*_i_).^13^ Data represent mean values ± standard error of the mean (SEM) (p*K*_i_, pEC_50_) or mean values (*K*_i_, EC_50_) from at least three independent experiments performed in triplicate. n.d. = not determined.

**Scheme 2:**
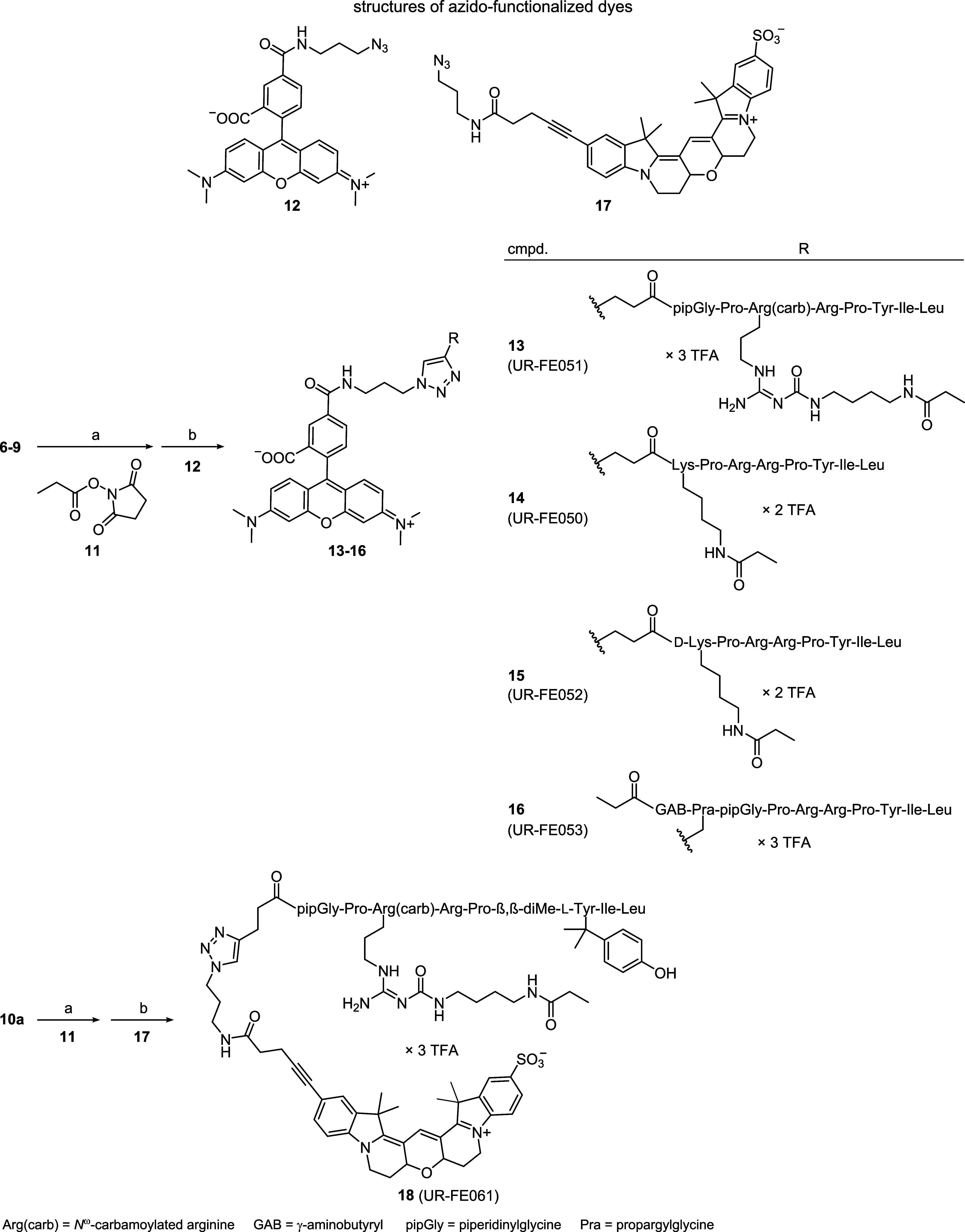
Synthesis of the Fluorescently Labeled Peptides **13**–**16** and **18** Reagents and conditions: (a) DIPEA, DMF, rt, 1–2 h; (b) CuSO_4_, sodium ascorbate, NMP/PBS 1:1 v/v, rt, 2 h; overall yields 35% (**13**), 48% (**14**), 43% (**15**), 12% (**16**), 15% (**18**).

To obtain the nontritiated 5-TAMRA-labeled ligands **13**–**16**, the precursor peptides **6**–**9** were first treated with succinimidyl propionate (**11**) followed by a copper(I)-catalyzed azide–alkyne 1,3-dipolar cycloaddition using the azido-functionalized 5-TAMRA derivative of **12** (Scheme 2). Propionylation of **10a** and subsequent click reaction with the azido-functionalized Cy3B derivative **17** (synthesis shown in Scheme S1, Supporting Information) gave fluorescent ligand **18**.

From the series of fluorescent peptides (**13**–**16**, **18**), the tritiated analogues were prepared for the 5-TAMRA-labeled peptide **13** (UR-FE051) and the Cy3B-conjugated peptide **18** (UR-FE061). The selection of **13** from the 5-TAMRA-labeled compound series (**13**–**16**) was guided by the NTS_1_R binding affinity and the synthetic accessibility of the respective precursor peptide: **13** and **16** showed the highest NTS_1_R binding affinity with comparable *K*_i_ values of 1.1 and 0.77 nM, respectively (Table 1). Looking at the synthesis of their precursor peptides **6** and **9**, the purification of **9**, containing the unnatural amino acid Pra, was considerably less convenient compared to **6** (discussed afore). Therefore, **13** was selected from the series of 5-TAMRA-labeled peptides.

**Figure 2:**
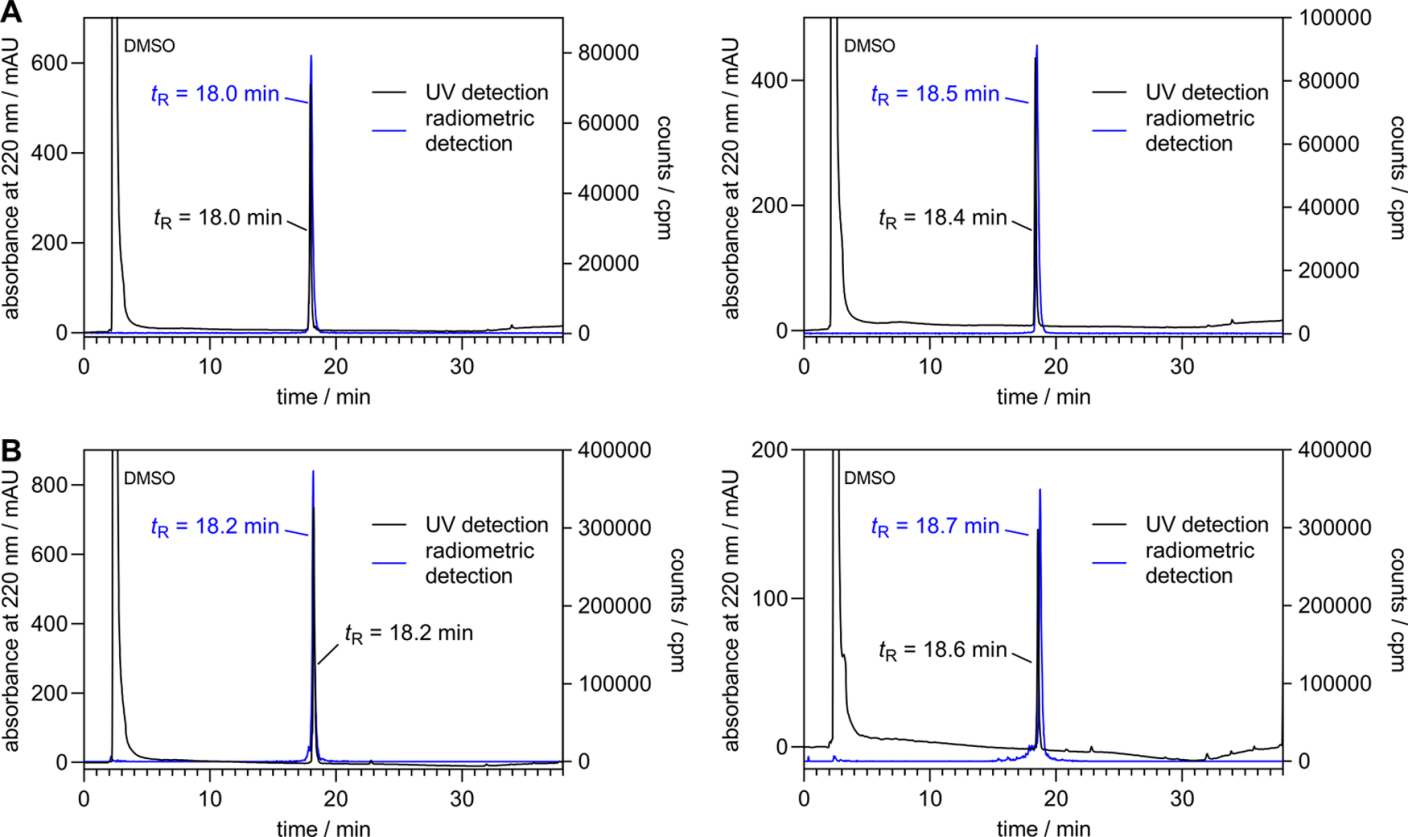
(A) Radiochemical purities (>99%) of [^3^H]**13** and [^3^H]**18** directly after synthesis determined by RP-HPLC analysis. (B) Radiochemical purities of [^3^H]**13** and [^3^H]**18** determined 11 months after synthesis. The purities amounted to 92% ([^3^H]**13**) and 80% ([^3^H]**18**).

**Scheme 3:**
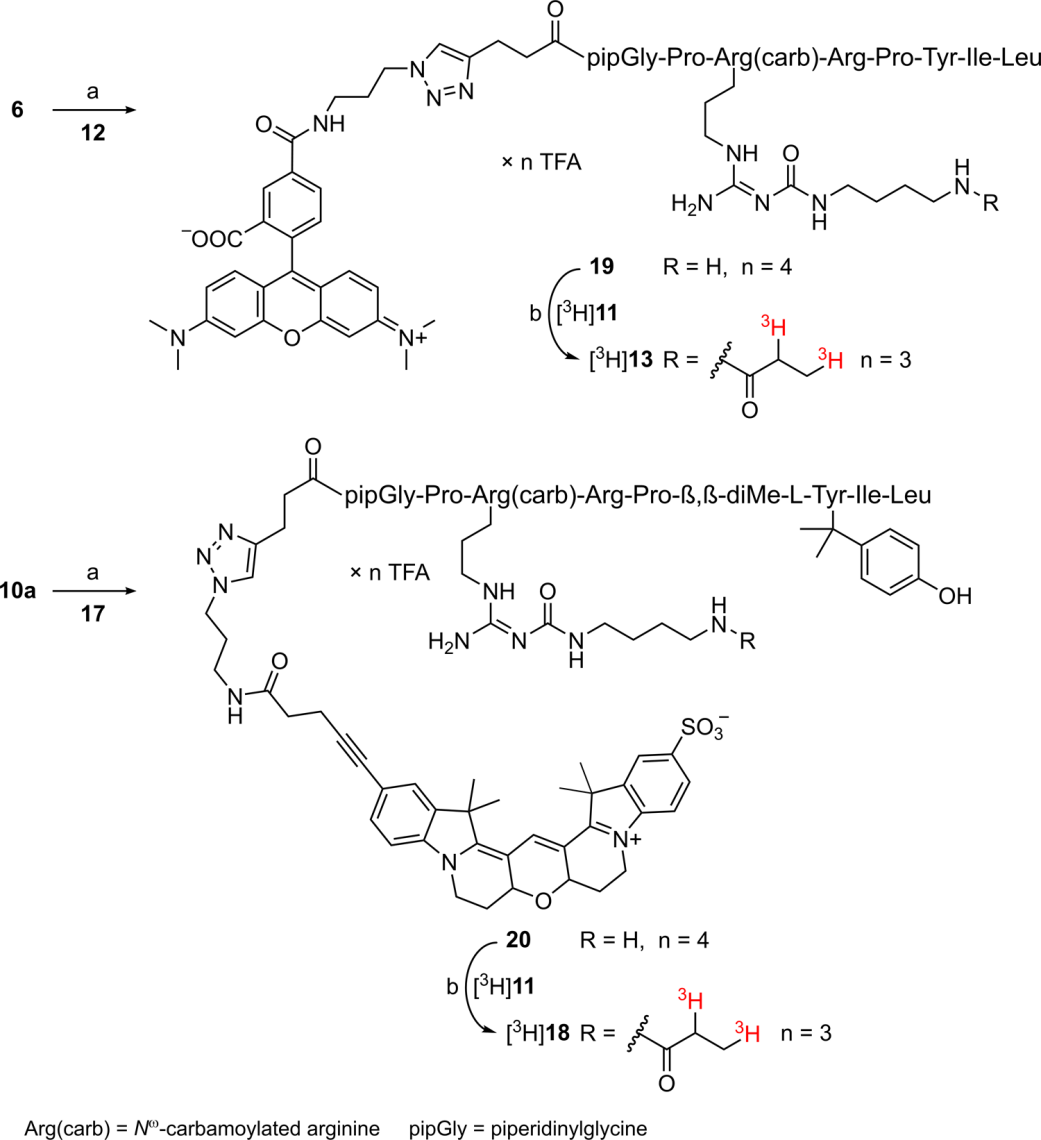
Synthesis of the Dually Labeled NTS_1_R Ligands [^3^H]**13** and [^3^H]**18** Reagents and conditions: (a) CuSO_4_, sodium ascorbate, NMP/PBS 1:1 v/v, rt, 2 h, 61% (**19**), 34% (**20**); (b) DIPEA, DMF/NMP 75:25 v/v, rt, 3 h, radiochemical yields 35% ([^3^H]**13**), 45% ([^3^H]**18**). Note that the tritium atoms in the [^3^H]propionyl residues do not represent a quantity of tritium isotopes; they only indicate that tritium is present in the respective position.

For the synthesis of the tritiated ligands [^3^H]**13** and [^3^H]**18**, the precursor peptides **6** and **10a** were conjugated to the fluorescent dyes 5-TAMRA and Cy3B, respectively, to afford **19** and **20** (Scheme 3). Treatment of an excess of **19** and **20** with [^3^H]**11** gave the tritiated fluorescent ligands [^3^H]**13** and [^3^H]**18**, respectively, which were obtained in high radiochemical purity after purification by RP-HPLC using an analytical HPLC system (Figure 2A).

Over a period of 11 months, degradation of both dually labeled ligands by radiolysis was low (Figure 2B).

### Chemical and Proteolytic Stability of **13** and **18**

The stability of the fluorescently labeled peptides **13** and **18** was studied in phosphate-buffered saline (PBS, pH 7.4) at room temperature over 48 h and in human plasma at 37 °C over 48 h. In PBS, **13** showed a very slow degradation observable after 24 h of incubation (Figure S2, Supporting Information). Peptide **18** proved to be highly stable over 48 h (Figure S3, Supporting Information). Interestingly, both **13** and **18** exhibited high stability in human plasma (>50% intact peptide after 24 h) (see also Table S1, Supporting Information). An N-terminal degradation of **13** and **18** by proteases is considered unlikely due to the N-terminal modification of the peptides (attachment of a fluorescent dye via the 4-pentynoyl moiety). In the case of **13**, containing an unmodified, i.e., the same C-terminal sequence (···Pro-Tyr-Ile-Leu) as NT or NT(8–13), a rapid C-terminal degradation by proteases was expected (e.g., cleavage of the C-terminal dipeptide Ile-Leu^33^) because for NT(8–13) analogues, containing this C-terminal sequence, a low proteolytic stability was reported.^27,31,34−38^ Presumably, the presence of the bulky fluorescent dye at the N-terminus combined with the *N*^ω^-carbamoylated arginine in position 11 of **13** impedes C-terminal degradation of the peptide by proteases. Unlike **13**, a high stability of **18** in blood plasma was indeed expected due to the presence of β,β-dimethyl-tyrosine in position 11, reported to stabilize NT(8–13) analogues against C-terminal proteolytic degradation.^31^

### Neurotensin Receptor Binding and Agonism

NTS_1_R binding affinities were determined in a reported competition binding assay^13^ using intact hNTS_1_R-expressing HT-29 colon carcinoma cells and [^3^H]UR-MK300 (structure see Figure 1A) as radioligand (competition binding curves shown in Figure S4, Supporting Information). The amino- and alkyne-functionalized precursor peptides **6**–**9** and **10a** displayed high NTS_1_R affinity with *K*_i_ values ≤1.1 nM (Table 1). Compound **10b**, containing β,β-dimethyl-d-tyrosine in position 11, showed considerably lower affinity (*K*_i_ = 140 nM). Propionylation and conjugation of the precursor peptides **6**–**9** and **10a** to the fluorescent dyes 5-TAMRA or Cy3B did only marginally affect NTS_1_R binding as becomes obvious from the high binding affinities of the fluorescent peptides **13**–**16** and **18** (*K*_i_ values: 0.77–4.2 nM).

For peptides **13** and **18**, NTS_2_R binding affinities were determined at homogenates of HEK293T-hNTS_2_R cells also using [^3^H]UR-MK300 as radiolabeled probe. These studies revealed that **13** and **18** bind to the NTS_2_R with almost equal affinity as to NTS_1_R (Table 1). For the present study, NTS_1_R selectivity was not needed since all cellular systems (CHO cells, HT-29 cells, Sf9 insect cells for BBV production) used for binding experiments expressed NTS_1_R, but not NTS_2_R. CHO cells and Sf9 cells were transfected with a vector encoding for the NTS_1_R, and in the case of HT-29 cells, endogenously expressing NTS_1_R, the absence of NTS_2_R was proven previously.^13^

NTS_1_R agonism was investigated for NT(8–13), the precursor peptides **6** and **10a**, and the fluorescently labeled peptides **13** and **18** in a Fura-2 Ca^2+^ assay using CHO-hNTS_1_R cells (concentration–response curves shown in Figure S5, Supporting Information). In general, all studied compounds proved to be full agonists and showed higher pEC_50_ values (0.5–2 orders of magnitude) compared to the respective p*K*_i_ values (Table 1). The high pEC_50_ values can be explained by the overexpression of NTS_1_R in stably transfected CHO-hNTS_1_R cells (ca. 300,000 receptors/cell^13^) resulting in an excess of receptors over intracellular G_q_-proteins (receptor reserve). In this case, a low receptor occupancy is sufficient to induce the maximal cellular response leading to apparently higher potencies.

### Fluorescence Characterization

For the fluorescently labeled peptides **13** and **18**, which were studied in various fluorescence-based binding assays (see below), excitation and emission spectra were recorded in PBS and PBS supplemented with 1% BSA (spectra shown in Figure S6, Supporting Information; excitation and emission maxima summarized in Table 2). Fluorescence quantum yields of **13** and **18** were determined in the same solvents. While the emission quantum yield of the 5-TAMRA-labeled ligand **13** was equal in neat PBS and PBS with 1% BSA, the quantum yield of the Cy3B-labeled ligand **18** was markedly reduced in the presence of BSA (Table 2) indicating either an impact of a potential unspecific interaction of the dye with the protein on its photophysical relaxation pathways or a (diffusion-controlled or within the aforementioned unspecific complex) reaction between the excited dye and a solvent exposed amino acid. Interestingly, a recently reported Cy3B-labeled peptidic neuropeptide Y Y_4_ receptor ligand showed almost equal quantum yields in PBS and PBS supplemented with 1% BSA (69 vs 67%),^39^ indicating that this ligand exhibits less interactions with BSA or a different binding motif for attaching to BSA.

**Table 2:** Excitation and Emission Maxima and Fluorescence Quantum Yields of **13** and **18**

	λ_ex_ [nm]/λ_em_ [nm] | Δ[eV]		Φ (%)	
compd.	PBS	PBS + 1% BSA	PBS	PBS + 1% BSA
**13**	558/584 | 0.10	559/583 | 0.091	45	44
**18**	571/584 | 0.048	571/585 | 0.052	77	62

### Investigation of NTS_1_R Binding of **13**, [^3^H]**13**, **18**, and [^3^H]**18** in Fluorescence-Based and Radiochemical Assays

Equilibrium saturation binding, association and dissociation kinetics, and competition binding of **13** and **18** were studied by three different fluorescence-based methods (HCI, FC, and FA). HCI and FC experiments were performed with intact CHO-hNTS_1_R cells. It should be noted that HCI necessitates the use of adherent cells, whereas FC requires the use of cell suspensions. Unlike HCI and FC, FA binding experiments were performed with hNTS_1_R-displaying BBVs. In analogy to the characterization of **13** and **18** in fluorescence-based assays, NTS_1_R binding of the dually labeled ligands [^3^H]**13** and [^3^H]**18** was studied in radiochemical assays. To enable a close comparison with HCI and FC, these experiments were carried out with adherent and suspended CHO-hNTS_1_R cells. For all binding studies, DPBS was used as binding buffer slightly varying with respect to the supplements (for details see Experimental Section). The assay temperature was 23 °C except for FA-based assays, which were performed at 27 °C. Concerning the fluorescence-based methods, measurements under homogeneous conditions were feasible with FC and FA. In the case of HCI, measurements were performed under nonhomogeneous conditions since a washing step was performed immediately before plate reading for the purpose of fluorescence background reduction.

As the obtained binding data of **13** and **18**, as well as [^3^H]**13** and [^3^H]**18** were similar and for the purpose of better comprehension, here we present and discuss the data of **13** and [^3^H]**13** in the first place. Graphs showing saturation binding, association and dissociation kinetics and displacement curves (competition binding) of **18** and [^3^H]**18** are shown in Figures S7–S9 (Supporting Information).

#### Equilibrium Saturation Binding with **13**, [^3^H]**13**, **18**, and [^3^H]**18**

**Figure 3:**
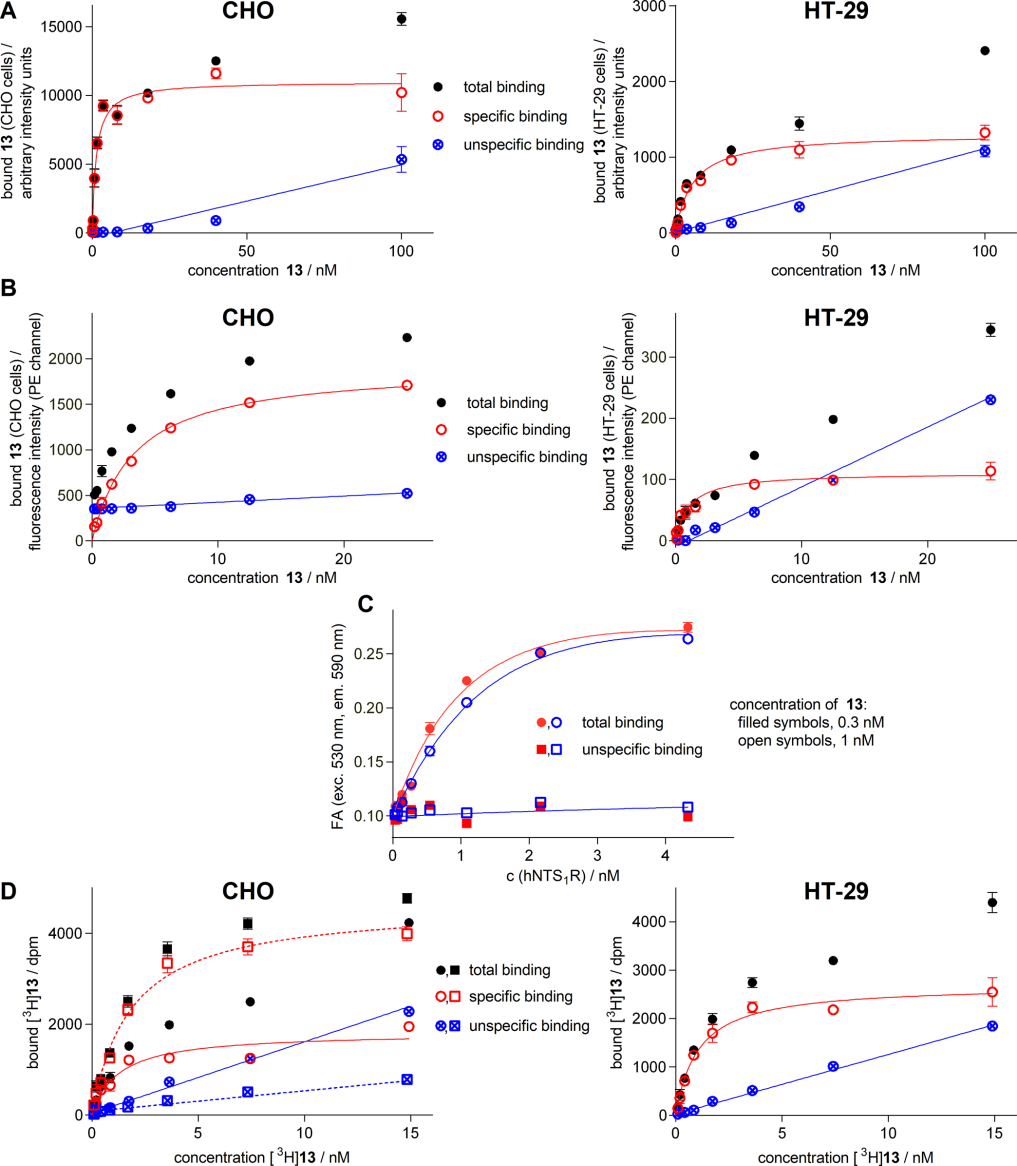
NTS_1_R equilibrium binding of **13** and [^3^H]**13** studied by different methods. (A) Binding isotherms (specific binding, open symbols) of **13** obtained from HCI binding experiments performed at intact CHO-hNTS_1_R and HT-29 cells (incubation: 90 min at 23 °C). (B) Binding isotherms (specific binding, open symbols) of **13** obtained from FC saturation binding experiments performed at intact CHO-hNTS_1_R and HT-29 cells (incubation: 90 min at 23 °C). (C) Binding isotherms (total binding, circles) of **13** obtained from FA-based binding experiments using fixed concentrations of **13** (0.3 or 1 nM) and increasing amounts of NTS_1_R-displaying BBVs (depicted data represent snapshots at 10 min incubation at 27 °C). (D) Binding isotherms of [^3^H]**13** from radiochemical saturation binding experiments performed at intact CHO-hNTS_1_R cells (adherent and in suspension) and at adherent HT-29 cells (incubation: 90 min at 23 °C). Circles represent adherent cells, squares represent suspended cells. Unspecific binding was determined in the presence of 1 μM NT(8–13) (A, B, and D) or 1 μM SR142948 (C). *K*_d_ values are presented in Table 3. Data represent mean values ± SEM (total and unspecific binding) or calculated values ± propagated error (specific binding) from representative experiments performed in triplicate (A, B, and D) or duplicate (C).

HCI, FC, and radiochemical equilibrium binding experiments, using increasing concentrations of the title compounds, yielded saturable NTS_1_R binding throughout (Figure 3; Figure S7, Supporting Information). Unlike the HCI, FC and radiochemical binding assays, requiring a large excess of the labeled ligand relative to the receptor concentration, FA measurements require approximately equal concentrations of the labeled ligand and receptor. Therefore, ligand depletion needs to be taken into account in the case of FA binding assays. For a detailed introduction to FA binding assays, see, e.g., Rinken et al.^21^ All saturation binding experiments were performed in 96-well plates. In the case of FC, this was feasible since the used flow cytometer (FACSCanto II, Becton Dickinson) was equipped with an autosampler (HTS unit) allowing an automated injection from microtiter plates.

The dissociation constants (*K*_d_ values) obtained from the different binding assays performed with intact CHO-hNTS_1_R cells (HCI, FC, radiochemical assay) or BBVs (FA) were in excellent agreement (Table 3, row 1). The *K*_d_ values were also in good agreement with the *K*_i_ values of **13** and **18** determined by competition binding with [^3^H]UR-MK300 at HT-29 colon carcinoma cells (see Table 1). To challenge the HCI, FC, and radiochemical assay, saturation binding of **13**, [^3^H]**13**, **18**, and [^3^H]**18** was also studied at HT-29 cells showing a considerably lower NTS_1_R expression compared to CHO-hNTS_1_R cells.^13^ Although specific binding was lower in absolute values and consequently also relative to unspecific binding due to the lower receptor expression, well reproducible saturation isotherms could be obtained (Figure 3A,C; Figure S7A,C, Supporting Information). However, the *K*_d_ values were slightly less consistent compared to the *K*_d_ values obtained from experiments with CHO-hNTS_1_R cells (Table 3).

**Table 3:** Parameters Characterizing NTS_1_R Binding of **13**, [^3^H]**13**, **18**, and [^3^H]**18** Determined in Different Types of Binding Assays

						radiochemical	
parameter	ligand	receptor source	HCI (adherent)	FC (suspension)	FA	adherent	suspension
*K*_d_ [nM]	**13**, [^3^H]**13**	CHO-hNTS_1_R cells	1.1 ± 0.2	2.6 ± 0.5	-	1.3 ± 0.1	1.8 ± 0.2
		HT-29 cells	4.7 ± 1.1	0.25 ± 0.1	-	1.2 ± 0.1	n.d.
		NTS_1_R-BBV	-	-	1.3 ± 0.1	-	-
	**18**, [^3^H]**18**	CHO-hNTS_1_R cells	1.3 ± 0.3	3.1 ± 0.3		1.1 ± 0.2	1.2 ± 0.1
		HT-29 cells	2.3 ± 0.3	0.65 ± 0.09		1.9 ± 0.3	n.d.
		NTS_1_R-BBV	-	-	1.5 ± 0.1	-	-
*k*_obs_ [min^–1^]	**13**, [^3^H]**13**	CHO-hNTS_1_R cells	0.084 ± 0.003	0.50 ± 0.08	-	0.57 ± 0.08^a^	1.5 ± 0.3^a^
						0.011 ± 0.001^b^	0.051 ± 0.001^b^
		NTS_1_R-BBV	-	-	n.a.	-	-
	**18**, [^3^H]**18**	CHO-hNTS_1_R cells	0.072 ± 0.006	0.18 ± 0.07	-	0.14 ± 0.03^a^	0.15 ± 0.03^a^
						0.013 ± 0.002^b^	0.006 ± 0.002^b^
		NTS_1_R-BBV	-	-	n.a.	-	-
*k*_off_ [min^–1^]	**13**, [^3^H]**13**	CHO-hNTS_1_R cells	0.044 ± 0.002	0.020 ± 0.002	-	0.014 ± 0.04	0.036 ± 0.006
		NTS_1_R-BBV	-	-	0.61 ± 0.03	-	-
	**18**, [^3^H]**18**	CHO-hNTS_1_R cells	0.051 ± 0.002	0.024 ± 0.002	-	0.026 ± 0.007	0.030 ± 0.004
		NTS_1_R-BBV	-	-	0.56 ± 0.04	-	-
*k*_on_ [min^–1^ nM^–1^]	**13**, [^3^H]**13**	CHO-hNTS_1_R cells	0.016 ± 0.002	0.48 ± 0.09	-	0.33 ± 0.09^c^	0.81 ± 0.17^c^
		NTS_1_R-BBV	-	-	1.0 ± 0.2	-	-
	**18**, [^3^H]**18**	CHO-hNTS_1_R cells	0.0081 ± 0.003	0.16 ± 0.07	-	0.10 ± 0.04^c^	0.097 ± 0.13^c^
		NTS_1_R-BBV	-	-	1.4 ± 0.3	-	-
*K*_d_(kin) [nM]	**13**, [^3^H]**13**	CHO-hNTS_1_R cells	2.8 ± 0.5	0.041 ± 0.01		0.41 ± 0.24	0.044 ± 0.02
		NTS_1_R-BBV	-	-	1.0 ± 0.3	-	-
	**18**, [^3^H]**18**	CHO-hNTS_1_R cells	6.4 ± 2.7	0.15 ± 0.08	-	0.26 ± 0.16	0.31 ± 0.44
		NTS_1_R-BBV	-	-	1.4 ± 0.3	-	-

a *k*_obs(fast)_ of the monophasic exponential fit (data describing the initial association phase).

b *k*_obs(slow)_ of the biphasic exponential fit.

c *k*_on(fast)_ calculated from *k*_off_, *k*_obs(fast)_ and the ligand concentration used for the association experiments (note: *k*_on(slow)_ is not given as the values calculated from *k*_off_, *k*_obs(slow)_ and the ligand concentration were negative.). *K*_d_, *k*_obs_, and *k*_off_ values represent mean values ± SEM from at least three independent experiments; *k*_on_ and *K*_d_(kin) values represent calculated values ± propagated error (HCI, FC, radiochemical assay) or mean values ± SEM from at least three individual experiments (FA). n.a. = not applicable. n.d. = not determined.

#### Investigation of the Association and Dissociation Kinetics of **13**, [^3^H]**13**, **18**, and [^3^H]**18**

**Figure 4:**
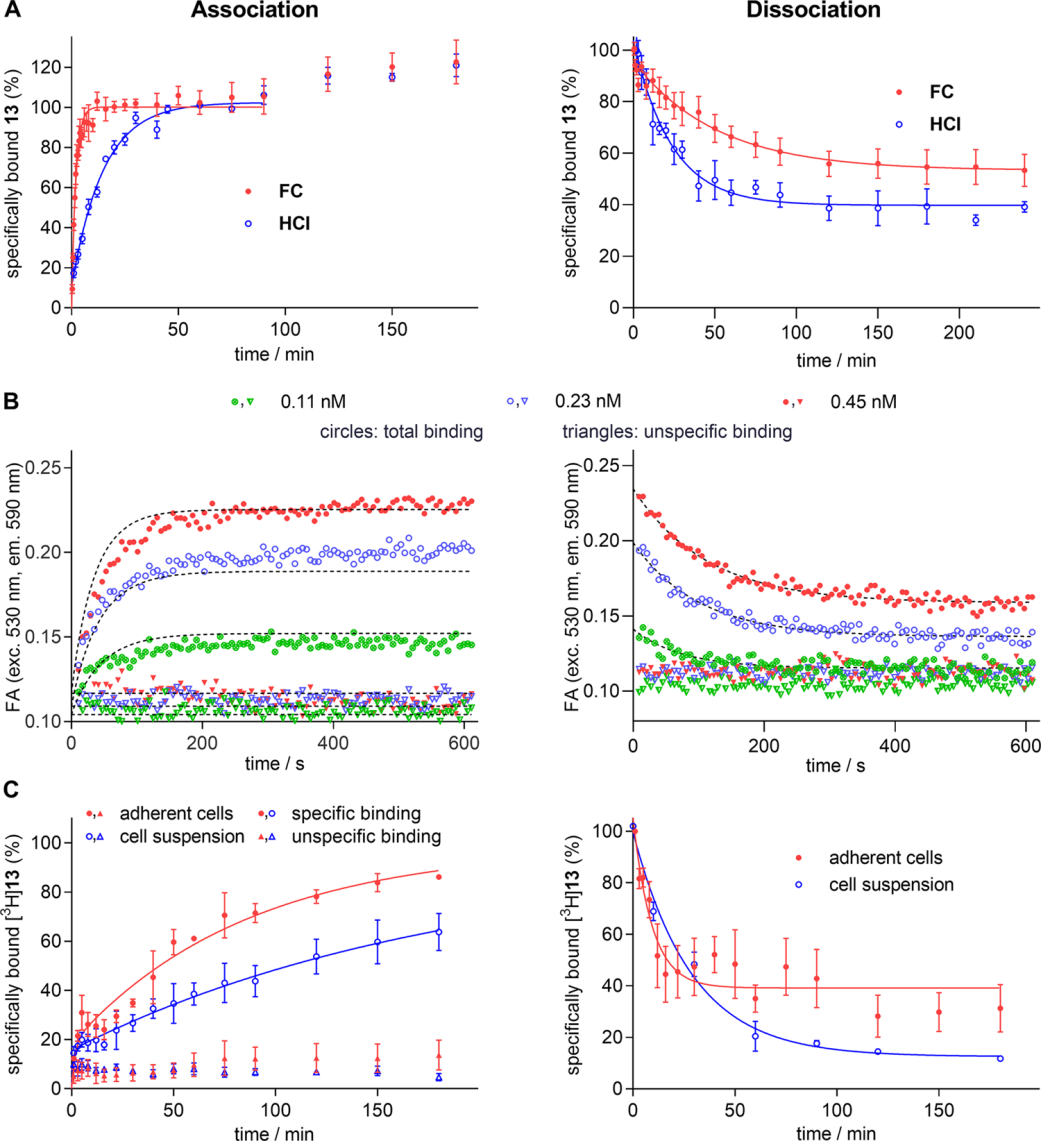
Binding kinetics of **13** and [^3^H]**13** at NTS_1_R studied by different methods. (A) Association and dissociation of **13** studied by HCI and FC at live adherent CHO-hNTS_1_R cells (HCI) or suspended CHO-hNTS_1_R cells (FC) at 23 °C. Concentrations of **13** used for the association: 2.5 nM (HCI) and 1 nM (FC); concentrations of **13** used during the preincubation period (90 min) of dissociation experiments: 2.5 nM (HCI) and 10 nM (FC). The last three data points were excluded from the fit. (B) Association and dissociation of **13** (0.3 nM) determined in an FA-based assay for three different NTS_1_R concentrations (green, blue, and red symbols) at 27 °C using NTS_1_R-displaying BBVs. Total binding is represented by circles and unspecific binding is represented by triangles. Following the association for up to 180 min did not reveal a second association phase (data not shown). (C) Association and dissociation of [^3^H]**13** studied at live adherent CHO-hNTS_1_R cells and suspended CHO-hNTS_1_R cells at 23 °C. Concentrations of **13** used for the association: 1.3 nM (adherent cells) and 1.8 nM (cell suspension); concentrations of **13** used during the preincubation period (90 min) of dissociation experiments: 6.5 nM (adherent cells) and 5 nM (cell suspension). For the biphasic association, unspecific binding, remaining at a constant level, is shown to demonstrate that both association phases account for binding to NTS_1_R. Proportion of fast/slow kinetic components (association): 19%/81% (adherent cells), 21%/79% (suspended cells). In the case of dissociation experiments performed with suspended cells, less times were studied because these experiments were laborious due to the use of 50 mL falcon tubes instead of 96-well plates (in this case, a separate workup process with the cell harvester had to be carried out for each time point). Dissociation and association rate constants are presented in Table 3. Data represent mean values ± SEM from at least three independent experiments performed in triplicate (A (HCI), C (association, dissociation at adherent cells)) or duplicate (A (FC), B, C (dissociation with cell suspensions)).

The association of the title compounds to NTS_1_R, studied at live CHO-hNTS_1_R cells (HCI, FC, radiochemical assay) or BBVs (FA), was nearly monophasic in the case of HCI, FC, and FA (Figure 4A,B; Figure S8A,B, Supporting Information). In the case of **13**, for both HCI and FC association experiments, a second, delayed (>90 min) phase was observed after the initial, clearly plateauing association phase (Figure 4A). Due to the late appearance and the slow rise of specific binding within the second phase relative to the initial association phase, data of the second slow phase were not included in the data analysis.

The radiochemical association experiments with [^3^H]**13** and [^3^H]**18** yielded a clear biphasic association (Figure 4C; Figure S8C, Supporting Information). It should be mentioned that the reproducibility of these experiments was lower compared to the fluorescence-based methods resulting in higher error bars. The variations of the values within a triplicate were also increased. This phenomenon was even more pronounced in the case of radiochemical dissociation experiments as discussed below. As fitting of the data using the two phase association fit (GraphPad Prism 5) failed for several individual experiments (ambiguous results), the following procedure was applied as an approximation: data describing the initial fast association phase, plateauing after ca. 10 min, were analyzed by a one phase association fit (GraphPad Prism 5) followed by a two phase association fit for which *k*_obs(fast)_ was constrained to the *k*_obs_ value obtained from the initial monophasic fit. Applying this approach, biphasic fitting of the data was feasible and *k*_obs(slow)_ values could be obtained (Figure 4C; Figure S8C, Supporting Information; Table 3).

Notably, a biphasic association was not observed for the radioligand [^3^H]UR-MK300 (adherent cells, same procedure as used for [^3^H]**13** and [^3^H]**18**), being devoid of a fluorescence label.^13^ Consequently, the fluorescent dye present in [^3^H]**13** and [^3^H]**18** seems to have an impact on the results of radiochemical association experiments. To further test the radiochemical association assay, [^3^H]**13** was also studied at membrane preparations of CHO-hNTS_1_R cells allowing a decoupling of G-protein from the receptor by the addition of GppNHp^40−47^ and precluding receptor internalization (discussed below). Comparing the radiochemical association experiments (cell suspension) with the FC assay, these two methods differ by nonhomogeneous vs homogeneous conditions and by the sample setup (96-well plates vs 5 mL tubes). To note, in contrast to FC saturation binding experiments, performed in 96 well plates, samples for FC association and dissociation experiments were prepared in 5 mL polypropylene tubes, i.e., data for the different time points originated from the same sample. These two main differences could also account for the observed differences in the association curves and the robustness of the data obtained from the radiochemical and the FC assay. As the radiochemical and the FC assay both use intact suspended CHO-hNTS_1_R cells, an internalization of the ligand–receptor complex would occur in either case, suggesting that the pronounced biphasic association curve observed for the radiochemical assay cannot be explained by endocytosis of ligand-bound NTS_1_R (NTS_1_R-mediated uptake of **13** and **18** in adherent CHO-hNTS_1_R cells was proven by confocal microscopy, see Figures S10–S15, Supporting Information). Likewise, the difference between the association curve radiochemically determined at adherent CHO-hNTS_1_R cells and the association curve obtained from HCI experiments (cf. Figure 4A,C, and Figure S8A,C, Supporting Information), can most likely not be attributed to an internalization of ligand–receptor complex since the cellular uptake can take place in both cases. As discussed below, radiochemical association experiments with [^3^H]**13** using membranes of CHO-hNTS_1_R cells (receptor uncoupled from G-protein) indicated that the biphasic association can be attributed to the presence of two subpopulations of NTS_1_R (coupled to and uncoupled from G-protein) in intact cells. Thus, it remains a matter of speculation why the fluorescence-based methods cannot reproduce the biphasic association curves obtained from radiochemical association experiments. This method-dependent bias needs to be explored in future studies. Altered fluorescence properties (upon ligand binding and/or cellular uptake) and different sensitivities for the detection of bound ligand could be potential reasons.

The hydrogen-tritium exchange in the propionyl group is considered to have no effect since the propionyl residue in UR-MK300, located at the same position as in **13** (Arg^8^), does not contribute to receptor binding.^13^ Comparing HCI with FC, the association of **13** to NTS_1_R determined by FC was considerably faster than observed by HCI (Figure 4A, *k*_on_ values presented in Table 3), which could also be attributed to nonhomogeneous vs homogeneous conditions and the different sample setup (adherent cells in 96-well plates vs suspended cells in 5 mL tubes).

Dissociation curves obtained from HCI, FC, FA, and radiochemical assays were all nearly monophasic (Figure 4; Figure S8, Supporting Information) giving similar *k*_off_ values except FA, which gave higher *k*_off_ values (Table 3). However, the dissociation curves differed considerably in terms of the plateaus representing long-lasting binding or irreversibly bound ligand. The highest plateaus were observed for HCI (**13**: 40%, **18**: 70%) and FC (**13**: 53%, **18**: 64%). An incomplete dissociation from NTS_1_R can be explained by internalization of the ligand–receptor complex and intracellular dissociation of fluorescent ligand from the receptor. Indeed, an NTS_1_R-mediated cellular uptake of **13** and **18** was confirmed by confocal microscopy (Figures S10–S15, Supporting Information) and the images from HCI dissociation experiments showed that the decrease in intracellular fluorescence stopped at a certain level during the dissociation process (Figure S16, Supporting Information). In the case of radiochemical dissociation experiments performed with adherent cells, the reproducibility was considerably lower compared to HCI resulting in high error bars (Figure 4C; Figure S8C, Supporting Information). When using cell suspensions, the radiochemical dissociation experiments performed in 96-well plates resulted in no reproducible data at all (Figure S17A, Supporting Information) (to note, these experiments were performed by three different operators experienced in studying ligand binding kinetics in radiochemical assays). Interestingly, the use of a sample setup similar to that used for FC (sample preparation in 50 mL falcon tubes instead of 96-well plates), allowing data acquisition for the various time points from the same sample, had a dramatic effect on the results: these experiments resulted in highly reproducible monophasic dissociation curves with a low plateau of 12% (**13**) and 18% (**18**) (blue curves in Figure 4C and Figure S8C, Supporting Information). These results suggest that the high variability of the data obtained from radiochemical dissociation experiments performed in 96-well plates is indeed caused by the respective experimental setup (use of 96 well plates meaning separate sample preparations for each time point) and cannot be attributed to the cellular uptake of the radiolabeled fluorescent ligand by internalization of ligand–receptor complex. The observed differences with respect to the plateaus obtained from the different radiochemical dissociation experiments (cell suspensions (falcon tubes) vs intact adherent cells: 12 vs 39% (**13**, cf. Figure 4C), and 18 vs 39% (**18**, cf. Figure S8C, Supporting Information)) indicate that the underlying mechanisms (internalization and potentially externalization) are different in adherent cells compared to suspended cells. However, both HCI dissociation experiments (adherent cells) and FC dissociation studies (suspended cells) yielded pronounced plateaus comparable to that obtained for radiochemical dissociation experiments performed at adherent cells (Figure 4A,C; Figure S8A,C, Supporting Information). If the plateau is only caused by the cellular uptake of the ligand–receptor complex by internalization resulting in intracellularly “trapped” ligand, one would expect similar results for the fluorescence-based and the radiochemical assays. This inconsistency indicates that also unsteady photophysical properties of the fluorescent ligands might play a role as discussed below in more detail.

To further test the 96-well sample setup, dissociation experiments were also performed with the nonfluorescent radioligand [^3^H]UR-MK300 in 96-well plates at suspended CHO-hNTS_1_R cells. As these experiments gave reproducible monophasic dissociation curves indicating a complete dissociation (Figure S17B, Supporting Information), the irreproducibility of the radiochemical dissociation experiments with [^3^H]**13** and [^3^H]**18** (cell suspensions) can also be attributed to the fluorescent dye in these dually labeled ligands besides—or in conjunction with—the technical setup of the assay.

Regarding HCI, FA, and the radiochemical assay using adherent cells, the kinetically derived dissociation constants *K*_d_(kin), calculated from *k*_off_ and *k*_on_, deviated less than a factor of 5 from the *K*_d_ values determined in equilibrium saturation binding experiments (Table 3). For FC, the *K*_d_(kin) values of **13** and **18** were markedly lower than the *K*_d_ from saturation binding studies. This could be due to overestimated *k*_obs_ and/or underestimated *k*_off_ values by the FC method.

While the *K*_d_(kin) value of [^3^H]**18** determined at cell suspensions was only slightly lower than the *K*_d_ from saturation binding experiments, the *K*_d_(kin) value of [^3^H]**13** was markedly lower (factor 41) than the *K*_d_ (Table 3) suggesting that the results of kinetic experiments also depend on the type of fluorescent dye.

It should be emphasized that shifts of the excitation and emission spectra and changes in fluorescence quantum yield, potentially occurring upon receptor binding and cellular uptake of the fluorescent ligand by internalization of the ligand–receptor complex (*cf*. Figures S10–S15, Supporting Information), can impact the fluorescence signals detected by HCI and FC. In contrast, altered fluorescence properties usually do not substantially influence FA measurements since the parallel and perpendicular fluorescence detected by FA would be equally affected, thus accounting for a compensation of the effect. Presumably, besides the different sources of NTS_1_R (intact adherent cells, intact suspended cells, BBVs), these methodological differences account for the observed differences in association and dissociation kinetics between HCI and FA as well as FC and FA. The hypothesis of changes in fluorescence properties upon receptor binding or cellular uptake is supported by the results of the radiochemical dissociation experiments performed with suspended CHO-hNTS_1_R cells: these experiments, performed under the same conditions as FC dissociation studies, yielded an almost complete dissociation of [^3^H]**13** from the receptor (Figure 4C), whereas in the FC assay, a plateau at approximately 50% specifically bound ligand was found (Figure 4A) (to note, the radiochemical assay is unaffected by changes in the photophysics of the fluorescent dye).

#### Competition Binding Experiments Using **13**, [^3^H]**13**, **18**, and [^3^H]**18** as Labeled Probes

**Figure 5:**
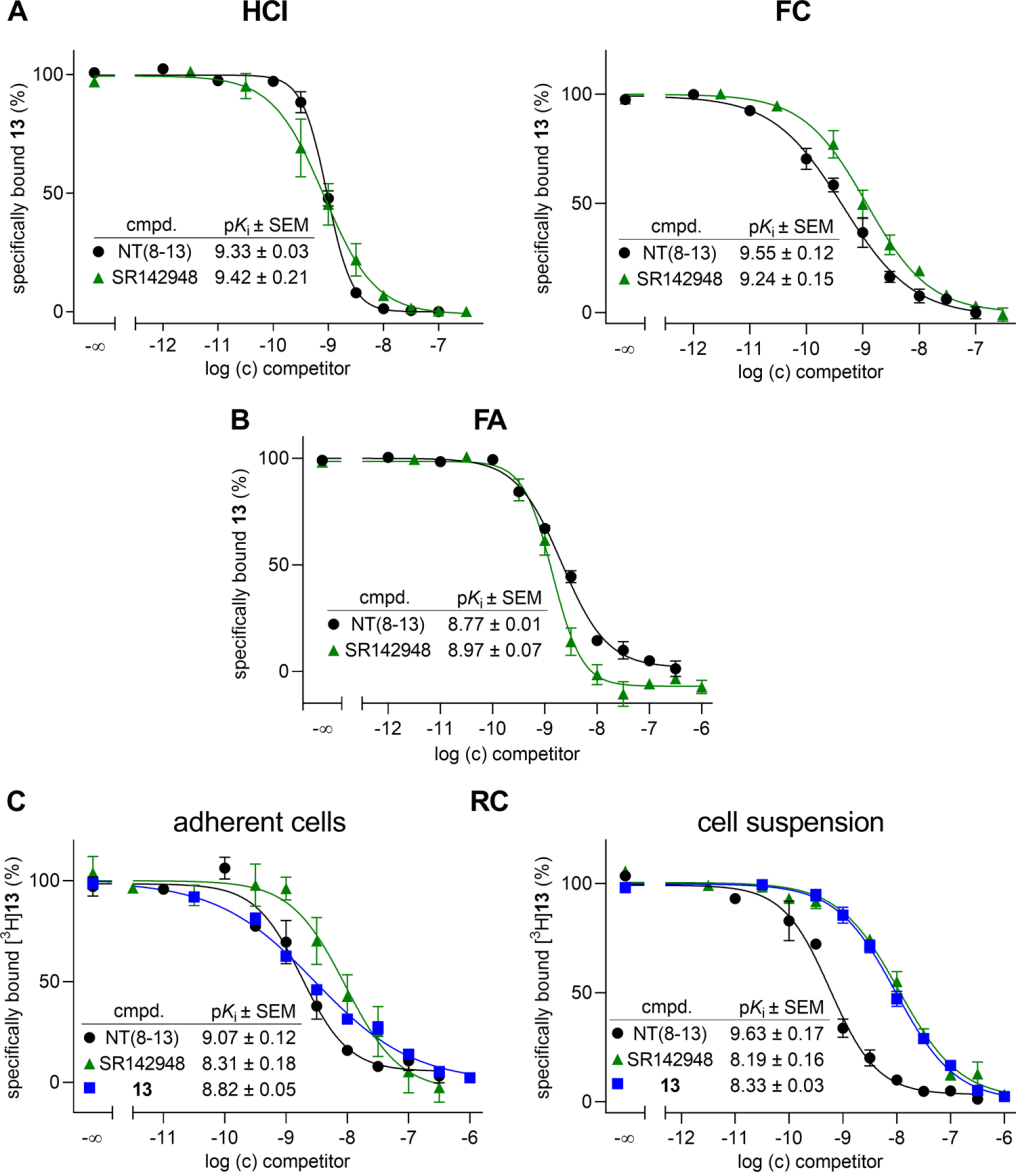
Displacement curves and corresponding p*K*_i_ values from competition binding studies performed with **13** or [^3^H]**13** and NT(8–13) and SR142948 using different types of binding assays (A: high-content imaging and flow cytometry; B: fluorescence anisotropy; C: radiochemical binding assay). Used concentrations of **13**: 1.1 nM (A (HCI)), 2.6 nM (A (FC)), 0.3 nM (B). Used concentrations of [^3^H]**13** (C): 1.3 nM (adherent cells), 1.8 nM (cell suspension). Incubation times: 90 min (A–C). Incubation temperatures: 23 °C (A, C), 27 °C (B). Data represent mean value ± SEM from at least three individual experiments performed in triplicate.

Like saturation binding assays, all competition binding experiments were performed in 96-well plates. For all kinds of studied NTS_1_R binding assays, the *K*_i_ values of the agonist NT(8–13) and the antagonist SR142948 were determined by displacing **13**, [^3^H]**13**, **18**, or [^3^H]**18** from NTS_1_R (displacement curves and p*K*_i_ values presented in Figure 5 and Figure S9, Supporting Information). Additionally, the p*K*_i_ values of **13** and **18** were determined in the radiochemical competition binding assays. For HCI and FC, the obtained NTS_1_R affinities of NT(8–13) and SR142948 were in good agreement with reported binding data (NT(8–13): *K*_i_ 0.14 nM,^13,48^ 0.24 nM,^49^ 0.29 nM,^50^ 1.0 nM;^51^ SR142948: *K*_i_ 1.0 nM,^52^ 1.1 nM^13^). The NTS_1_R binding affinities of NT(8–13) obtained from the FA and the radiochemical assay using adherent cells were slightly lower compared to reported data.^13,48−51^ NTS_1_R affinities of SR142948 determined with **13** and **18** in the FA assay (p*K*_i_ = 8.97 and 9.19, respectively) were perfectly in line with reported data.^13,52^ The NTS_1_R binding affinity of NT(8–13) obtained from the radiochemical competition binding assays using cell suspensions were in good agreement with the aforementioned literature data. In contrast, NTS_1_R affinities of SR142948 determined by competition binding with [^3^H]**13** and [^3^H]**18** (adherent and suspended cells) were slightly lower than the aforementioned literature data. Noteworthy, the NTS_1_R binding affinities of **13** and **18** obtained by competition binding with their tritiated analogues were in good agreement with the *K*_d_ values of [^3^H]**13** and [^3^H]**18** determined by saturation binding (see Table 3, Figure 5C, and Figure S9C, Supporting Information).

#### Binding of [^3^H]**13** to Membranes of CHO-hNTS_1_R Cells

**Figure 6:**
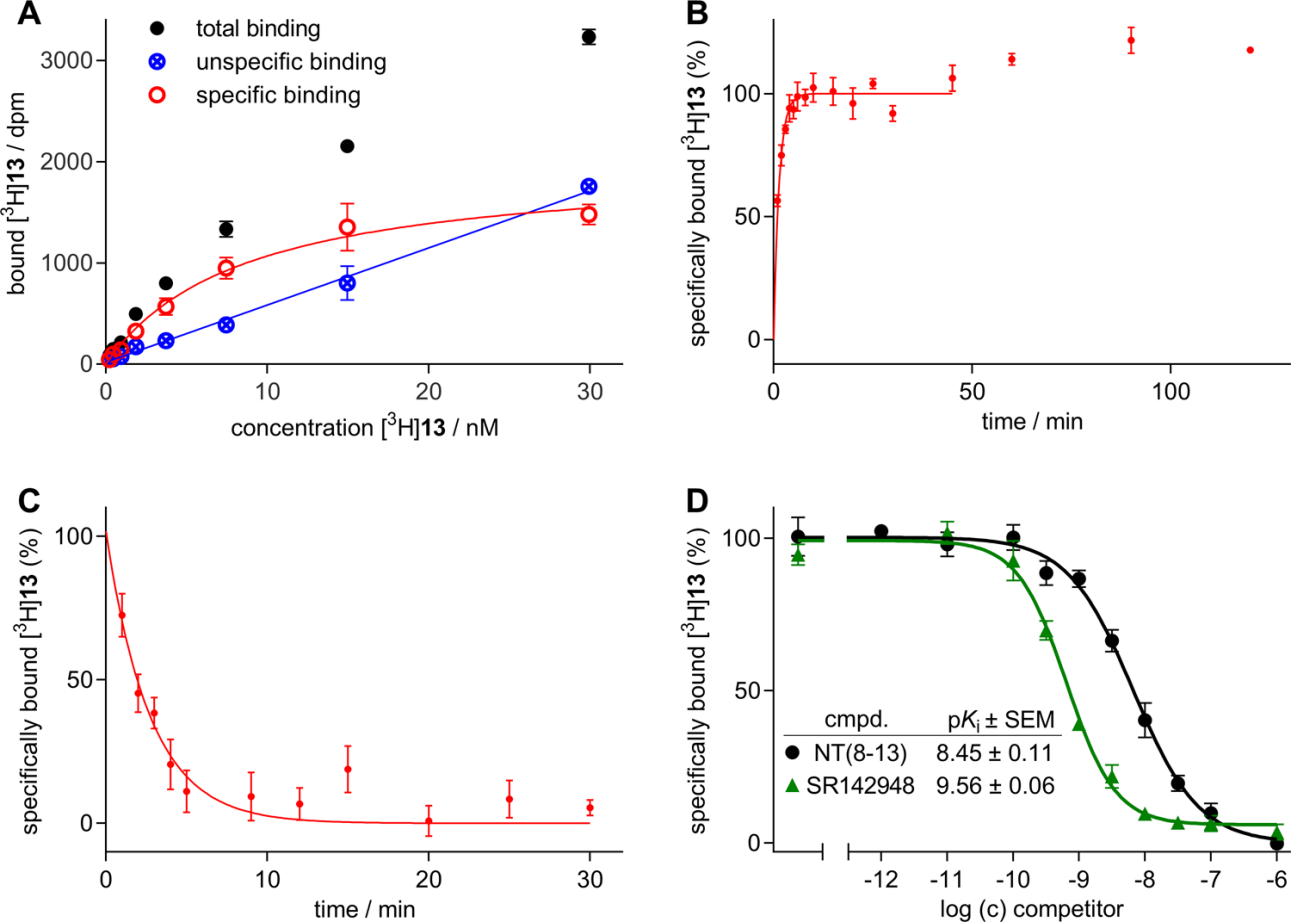
NTS_1_R binding characteristics of [^3^H]**13** studied at membranes of CHO-hNTS_1_R cells at 23 °C. (A) Representative binding isotherm (specific binding, open symbols) of [^3^H]**13** obtained from a radiochemical saturation binding experiment. Data represent mean values ± SEM (total and unspecific binding) or calculated values ± propagated error (specific binding) from a representative experiment performed in triplicate. (B) Association of [^3^H]**13** (*c* = 2.5 nM) to NTS_1_R. The last three data points were excluded from the fit. Data represent mean values ± SEM obtained from five independent experiments performed in triplicate. (C) Dissociation of [^3^H]**13**. Concentration of [^3^H]**13** used for the preincubation (30 min): 12 nM. Data were normalized based on *Y*_0_ (100%) obtained from a one phase decay fit (GraphPad Prism). The fit (plateau value constrained to zero, for details see Experimental Section) was extrapolated to *t* = 0 min. Data represent mean values ± SEM obtained from three independent experiments performed in triplicate. (D) Displacement curves and corresponding p*K*_i_ values from competition binding studies performed with [^3^H]**13** (*c* = 6.2 nM) and NT(8–13) and SR142948. Data represent mean values ± SEM obtained from three independent experiments performed in triplicate.

As [^3^H]**13** showed a pronounced biphasic association when studied at intact CHO-hNTS_1_R cells (Figure 4C), NTS_1_R binding of this dually labeled peptide was additionally investigated using membrane preparations enabling the uncoupling of G-protein from the receptor, e.g., by adding the G-protein inhibitor GppNHp.^40−47^ These experiments were performed in 96-well plates using the same procedure as for radiochemical binding experiments with intact suspended cells, but using cell membranes instead of whole cells. Binding of [^3^H]**13** to the NTS_1_R was saturable (Figure 6A). As expected, the *K*_d_ value of [^3^H]**13** obtained from equilibrium saturation binding experiments at cell membranes in the presence of 50 μM GppNHp (*K*_d_ = 6.2 ± 1.3 nM, mean value ± SEM from three independent experiments performed in triplicate) was higher than the *K*_d_ determined at intact cells (*K*_d_ = 1.8 nM, cf. Table 3). To note, in addition to GppNHp, saponin (100 μg/mL) was added to the samples to improve ligand permeabilization into vesicles that are likely to occur in membrane preparations. The association curve of [^3^H]**13** obtained from experiments with cell membranes (Figure 6B) was considerably different compared to the association curve observed for intact suspended cells (cf. Figure 4C). The biphasic character almost disappeared and with a *k*_on_ value of 0.13 ± 0.05 nM^–1^ min^–1^ (calculated value ± propagated error) the association was slower compared to the initial phase found for experiments with intact cells (*k*_on_ = 0.81 nM^–1^ min^–1^, cf. Table 3). This indicated the existence of two subpopulations of NTS_1_R in intact cells, one population coupled to G-protein and the other not bound to G-protein as a consequence of the overexpression of NTS_1_R in CHO-hNTS_1_R cells. However, this conclusion is not in line with the data obtained from fluorescence-based (HCI, FC) association experiments at intact CHO-hNTS_1_R cells (Figure 4A), showing a course comparable to that found for the radiochemical association experiments performed with cell membranes (Figure 6B). As mentioned before, the exploration of this phenomenon requires additional studies.

Dissociation experiments with [^3^H]**13** at cell membranes were, unlike dissociation studies at intact suspended cells (sample preparation in 96-well plates), highly reproducible yielding a clearly monophasic curve and a complete dissociation (Figure 6C) with a *k*_off_ value of 0.37 ± 0.08 min^–1^ (mean value ± SEM from three independent experiments performed in triplicate). This revealed that the technical setup of the assay causes an irreproducibility of the radiochemical dissociation assay (suspended cells, sample preparation in 96-well plates; cf. Figure S17A, Supporting Information) (see also discussion above) only in conjunction with the use of intact cells, allowing a cellular uptake of ligand/agonist-receptor complex. The *K*_d_(kin) value, calculated from *k*_off_ and *k*_on_, amounted to 2.7 ± 1.6 nM (calculated value ± propagated error), deviating less than a factor of 2.5 from the *K*_d_ value obtained from saturation binding studies (*K*_d_ = 6.2 nM). When comparing the data of the dissociation kinetics obtained from the radiochemical assay performed at cell membranes with the results from the fluorescence-based methods, the highest agreement in *k*_off_ values is found for the FA assay (*k*_off_ ([^3^H]**13**) = 0.37 min^–1^, *k*_off_ (**13**, FA) = 0.61 min^–1^). Likewise, the *K*_d_(kin) values obtained from the radiochemical (cell membranes) and the FA assay showed the highest agreement with the *K*_d_ from saturation binding studies ([^3^H]**13**: *K*_d_(kin) = 2.7 nM, *K*_d_ = 6.2 nM; **13** (FA): *K*_d_(kin) = 1.0 nM, *K*_d_ = 1.3 nM). This is consistent with the fact that internalization of agonist-receptor complex cannot occur when membrane preparations or BBVs^53^ are used as receptor source, supporting an action according to the law of mass action.

The NTS_1_R binding affinities of NT(8–13) and SR142948, determined by competition binding with [^3^H]**13** at cell membranes (Figure 6D), were lower and higher, respectively, compared to the aforementioned reported binding data. This is in agreement with the uncoupling of the receptor from G-protein resulting in a destabilization of the active receptor conformation,^47^ and, in turn, in decreased agonist affinity and potentially in increased antagonist affinity (reported, e.g., for the β2 adrenergic receptor^47^ and the muscarinic acetylcholine M_2_ receptor).^42,54^

## Conclusions

The determination of ligand–receptor binding affinities usually requires labeled ligands that are used as probes in radiochemical or luminescence-based competition binding assays. A comparison of the different types of binding assays is limited due to the fact that radioligands, being structurally different from fluorescent ligands, cannot be used in fluorescence-based assays and vice versa. To enable a close comparison of radiochemical and fluorescence-based ligand–receptor binding assays, a proof-of-concept study was performed with two tritium-labeled fluorescent neurotensin receptor ligands ([^3^H]**13**, [^3^H]**18**) and their nontritiated analogues (**13**, **18**), which were characterized in radiochemical and fluorescence-based (high-content imaging, flow cytometry, and fluorescence anisotropy) NTS_1_R binding assays. While equilibrium binding experiments (saturation and competition binding) gave well comparable dissociation constants (*K*_d_, *K*_i_), the results from kinetic studies (association and dissociation experiments) were less consistent, not only when comparing radiochemical with fluorescence-based, but also among the fluorescence-based methods. Obviously, this is caused by the different kinds of signal detection in conjunction with effects mediated by the fluorescent dye. Indeed, the study revealed that the presence of a fluorescence label in a radioligand can cause a radiochemical assay to fail that works with a structurally closely related nonfluorescently labeled radioligand. Notably, the labeled ligands used in the present study represent NTS_1_R agonists inducing receptor internalization in intact cells, which likely affects the ligand binding kinetics. Therefore, the same study conducted with appropriately labeled antagonists, might give a different picture. In summary, the presented results demonstrate that despite marked differences in kinetic parameters, there is a solid agreement of *K*_d_ values (labeled ligands) and *K*_i_ values (unlabeled ligands) derived from the different methods. This suggests that, for binding assays using nonmodified (wild-type) receptors, the method-dependent bias is low with respect to the determination of ligand–receptor affinities. A main question raised by the present study is to what extent do the changing fluorescence properties (receptor-bound vs free fluorescent ligand) influence the results of fluorescence-based binding assays. This needs to be addressed in future studies by a systematic exploration of changes in the photophysics of fluorescent ligands upon receptor binding.

Beyond the purpose of the present study, i.e., the comparison of different kinds of binding assays, the introduced fluorescently labeled NTS_1_R ligands represent tool compounds useful to characterize nonlabeled NTS_1_R ligands. Besides the determination of binding affinities of orthosteric NTS_1_R ligands, they could also serve to characterize allosteric NTS_1_R modulators, which effect the binding affinity of orthosteric ligands. This includes novel chemotypes such as SBI-553^55^ addressing an intracellular allosteric binding site of NTS_1_R.

## Experimental Section

### Materials

The protected amino acids Fmoc-Tyr(tBu)–OH, Fmoc-Leu-OH, Fmoc-Arg(Pbf)–OH, Fmoc-d-Lys(Boc)–OH and Fmoc-Pra–OH (**2**) were purchased from Carbolution Chemicals (St. Ingbert, Germany). Fmoc-Lys(Boc)–OH, Fmoc-Pro-OH, Fmoc-Ile-OH, HBTU and H-Leu-2-ClTrt resin were from Merck (Darmstadt, Germany). Fmoc-pipGly(Boc)–OH (**3**) was from GL Biochem (Shanghai, China). Fmoc-β,β-dimethyl-Tyr(tBu)–OH (racemic) (**4**) was obtained from Iris Biotech (Marktredwitz, Germany). HOBt and Triton X-100 were from Sigma-Aldrich (Taufkirchen, Germany). NMP and DMF for peptide synthesis, anhydrous DMF and NMP, dichloromethane, piperidine, saponin and TFA were purchased from ACROS/FisherScientific (Schwerte, Germany). DIPEA was obtained from ABCR (Karslruhe, Germany). Acetonitrile (HPLC gradient grade) was from VWR (Ismaning, Germany). NT(8–13) was synthesized via SPPS in-house. SR142948 was purchased from Tocris Bioscience (Bristol, U.K.). Bacitracin, HEPES, and bovine serum albumin (BSA) were obtained from Serva (Heidelberg, Germany). Fetal bovine serum (FBS) was purchased from Pan-Biotech (Aidenbach, Germany) or Sigma. Succinimidyl [^3^H]propionate ([^3^H]**11**, molar activity: 105 Ci/mmol) was purchased from Novandi (Södertälje, Sweden). Fura-2 AM and Pluronic F-127 were obtained from Calbiochem/Merck Biosciences (Beeston, U.K.). GppNHP was purchased from Jena Biosciences (Jena, Germany). The syntheses of *N*-Boc-γ-aminobutyric acid succinimidyl ester^56^ and compound **21**(5) were described previously. The radioligand [^3^H]UR-MK300 (molar activity: 2.41 TBq/mmol) was prepared according to a described procedure.^13^ Compound **1**,^13^**5**,^31^ and **11**(57) were prepared according to the reported procedures. 5-TAMRA-azide (**12**) was purchased from Carl Roth (Karlsruhe, Germany). Millipore water was consistently used for the preparation of stock solutions, buffers, and aqueous eluents for HPLC. Polypropylene reaction vessels with screw cap (1.5 and 2 mL) from Sarstedt (Nümbrecht, Germany) were used for small-scale reactions (e.g., activation of Fmoc-protected amino acids) and to keep stock solutions.

### NMR Spectroscopy

NMR spectra were recorded on an AVANCE 600 instrument with cryogenic probe (^1^H: 600 MHz; ^13^C: 150 MHz) (Bruker, Karlsruhe, Germany). NMR spectra were calibrated based on the solvent residual peaks (^1^H NMR, DMSO-*d*_6_: δ = 2.50 ppm; ^13^C NMR, DMSO-*d*_6_: δ = 39.50 ppm), and data are reported as follows: ^1^H NMR: chemical shift δ in ppm (multiplicity [s = singlet, d = doublet, t = triplet, m = multiplet, and br s = broad singlet], integral, coupling constant *J* in Hz); ^13^C NMR: chemical shift δ in ppm.

### Mass Spectroscopy

High-resolution mass spectrometry (HRMS) was performed with an Agilent 6540 UHD accurate-mass Q-TOF LC/MS system coupled to an Agilent 1290 analytical HPLC system (Agilent Technologies. Santa Clara, CA) using an ESI source and the following LC method: column: Luna Omega C18, 1.6 μm, 50 × 2.1 mm (Phenomenex, Aschaffenburg, Germany), column temperature: 40 °C, solvent/linear gradient: 0–4 min: 0.1% aqueous HCOOH/acetonitrile supplemented with 0.1% HCOOH 95:5–2:98, 4–5 min: 2:98, flow: 0.6 mL/min.

### Preparative HPLC

Preparative HPLC was performed with a system from Knauer (Berlin, Germany) consisting of two K-1800 pumps and a K-2001 detector. A Gemini NX-C18, 5 μm, 250 mm × 21 mm (Phenomenex, Aschaffenburg, Germany) was used as stationary phase at a flow rate of 20 mL/min using mixtures of 0.1% aqueous TFA and acetonitrile as the mobile phase. A detection wavelength of 220 nm was used throughout. Collected fractions were lyophilized using a Scanvac CoolSafe 100–9 freeze-dryer (Labogene, Allerød, Denmark) equipped with a RZ 6 rotary vane vacuum pump (Vacuubrand, Wertheim, Germany).

### Analytical HPLC

Analytical HPLC analysis was performed with a system from Agilent Technologies composed of a 1290 Infinity binary pump equipped with a degasser, a 1290 Infinity autosampler, a 1290 Infinity thermostated column compartment, a 1260 Infinity diode array detector, and a 1260 Infinity fluorescence detector. A Kinetex-XB C18, 2.6 μm, 100 mm × 3 mm (Phenomenex) served as stationary phase at a flow rate of 0.6 mL/min. Detection was performed at 220 nm and the temperature of the column compartment was set to 25 °C. Mixtures of acetonitrile (A) and 0.04% aqueous TFA (B) were used as mobile phase. The following linear gradients were applied: compounds **6**–**9**, **10a**, **10b**, **19**, and **20**: 0–14 min: A/B 10:90–30:70, 14–15 min: 30:70–95:5, 15–18 min: 95:5 (isocratic); compounds **13**–**16** and **18**: 0–14 min: A/B 20:80–40:60, 14–15 min: 40:60–95:5, 15–18 min: 95:5 (isocratic); compound **17**: 0–14 min: A/B 25:75–45:55, 14–15 min: 45:55–95:5, 15–18 min: 95:5 (isocratic). The injection volume was 20 μL. Retention (capacity) factors *k* were calculated from the retention times *t*_R_ according to *k* = (*t*_R_ – *t*_0_)/*t*_0_ (*t*_0_ = dead time, 0.76 min for the used system and column).

### General Procedure for Solid Phase Peptide Synthesis

Peptides were synthesized manually by SPPS according to the Fmoc strategy. A H-Leu-2-ClTrt resin was used as solid phase and DMF/NMP 4:1 v/v was used as solvent. 5 mL NORM-JECT syringes (B. Braun-Melsungen, Melsungen, Germany), equipped with a 35-μm polypropylene frit (Roland Vetter Laborbedarf, Ammerbuch, Germany), were used as reaction vessels. The resin was allowed to swell in solvent for 30 min at rt. Fmoc-amino acids (except for **1**–**4**), used in 5-fold excess, were preactivated with HOBt/HBTU/DIPEA (5/4.9/10 equiv) in solvent (about 2.2 mL/mmol amino acid) for at least 5 min before addition to the resin. The Fmoc-protected unnatural amino acids **1**–**4** were used in 3-fold excess and were preactivated with HBTU/HOBt/DIPEA (3/3/6 equiv) in anhydrous solvent (about 1.6 mL/mmol amino acid) for 5–10 min prior to addition to the resin. Amino acid coupling was carried out on a shaker (Heidolph Multi Reax; Heidolph Instruments, Schwabach, Germany) covered with a thermostat controlled (35 °C) box. In the case of standard amino acids, “double” coupling (2 × 45 min) was performed. **1**–**4** were attached by a single coupling procedure (35 °C, 16 h). After coupling of an Fmoc-amino acid, the resin was washed with DMF/NMP 4:1 v/v (4 ×) followed by Fmoc deprotection using 20% piperidine in DMF/NMP 4:1 v/v (2 × 10 min at rt) and subsequent washing of the resin with solvent (6 × ca. 1 mL). After coupling of the last amino acid, final Fmoc deprotection and, in the case of **6**–**9**, **10a**, and **10b**, subsequent treatment with 10 equiv of succinimidyl 4-pentynoate (**5**) (peptides **6**–**8**, **10a**, and **10b**) or with 10 equiv of *N*-Boc-γ-aminobutyric acid succinimidyl ester (peptide **9**) in DMF/NMP 4:1 v/v in the presence of DIPEA (10 equiv) at 35 °C for 30 min, the resin was washed with DMF/NMP 8:2 v/v (6 ×) and CH_2_Cl_2_ (3 ×) (treated with potassium carbonate). Peptides were cleaved off the resin using CH_2_Cl_2_/TFA 3:1 v/v (2 × 20 min at rt). The liquids (2 × ca. 2 mL) were collected in a 100 mL round-bottom flask and the resin was washed once with CH_2_Cl_2_/TFA 3:1 v/v (2 mL). The volatiles of the combined liquids were removed by evaporation, TFA/H_2_O 95:5 v/v (2 mL per 100 mg resin) was added to the residue and the mixture was stirred at rt for 5 h. The volatiles were removed by evaporation followed by the addition of water (ca. 50 mL) and lyophilization to obtain the crude peptide, which was subjected to purification by preparative HPLC.

### Compound Characterization

Peptides **6**–**9** and **10a** were characterized by HRMS, ^1^H-, ^13^C-, and 2D-NMR spectroscopy (2D: ^1^H-COSY, HSQC, HMBC), and RP-HPLC. Compounds **13** and **18** were characterized by HRMS, ^1^H NMR spectroscopy, and RP-HPLC. **10b**, **14**–**17**, **19**, and **20** were characterized by HRMS and RP-HPLC. HPLC purities of all target compounds were ≥97% (UV detection, 220 nm).

### Experimental Protocols and Analytical Data

#### *N*^α^-(Pent-4-ynoyl)-2-(piperidin-4-yl)-Gly-Pro-*N*^ω^-[(4-aminobutyl)aminocarbonyl]Arg-Arg-Pro-Tyr-Ile-Leu Tetrakis(hydrotrifluoroacetate) (**6**)

Peptide **6** was synthesized on a H-Leu-2-ClTrt resin (60 mg, 0.79 mmol/g) according to the general procedure. Purification by preparative HPLC (gradient: 0–30 min: acetonitrile/0.1% aqueous TFA 15:85–35:65, *t*_R_ = 9 min) yielded **6** as a white fluffy solid (8.8 mg, 11%). ^1^H NMR (600 MHz, DMSO-*d*_6_): δ (ppm) 0.80 (t, 3H, *J* 7.6 Hz), 0.84 (d, 6H, *J* 6.5 Hz), 0.90 (d, 3H, *J* 6.7 Hz), 1.00–1.09 (m, 1H), 1.29–1.44 (m, 3H), 1.44–1.59 (m, 12H), 1.59–1.74 (m, 5H), 1.74–1.97 (m, 9H), 2.02–2.11 (m, 1H), 2.32–2.37 (m, 4H), 2.65–2.72 (m, 1H), 2.74–2.77 (t, 1H, 2.3 Hz), 2.77–2.90 (m, 5H), 3.01–3.16 (m, 4H), 3.18–3.33 (m, 4H), 3.46–3.60 (m, 3H), 3.68–3.74 (m, 1H), 4.17–4.26 (m, 3H, quantified in the spectrum acquired after the addition of D_2_O), 4.29–4.36 (m, 2H, quantified in the spectrum acquired after the addition of D_2_O), 4.37–4.50 (m, 3H, quantified in the spectrum acquired after the addition of D_2_O), 6.58–6.63 (m, 2H), 6.72–7.20 (br s, 2H, interfering with next listed signal), 6.97–7.01 (m, 2H), 7.20–7.56 (br s, 2H), 7.58–7.65 (br s, 1H), 7.67 (t, 1H, *J* 5.3 Hz), 7.73 (d, 1H, *J* 8.9 Hz), 7.76–7.85 (br s, 3H), 7.89 (d, 1H, *J* 7.9 Hz), 7.95 (d, 1H, *J* 7.5 Hz), 8.08 (d, 1H, *J* 7.3 Hz), 8.19 (t, 2H, *J* 7.2 Hz), 8.29–8.41 (m, 1H), 8.47 (s, 2H), 8.72–8.85 (d, 1H, *J* 8.8 Hz), 9.06 (s, 1H), 9.20 (br s, 1H), 10.67 (s, 1H), 12.51 (s, 1H). ^13^C NMR (150 MHz, DMSO-*d*_6_): δ (ppm) 10.91, 14.23, 15.12, 21.22, 22.84, 24.10, 24.16, 24.26, 24.37, 24.40, 24.49, 24.55, 25.04, 25.96 (2 carbon atoms), 28.39, 28.77, 29.06, 29.36, 33.74, 35.49, 36.37, 37.12, 38.47, 38.64, 39.73, 40.49 (2 carbon atoms), 42.74, 42.99, 46.73, 47.28, 49.86, 50.14, 51.99, 53.67, 54.12, 56.36, 59.18, 59.45, 71.37, 83.67,113.80 (TFA), 114.77 (2 carbon atoms), 115.77 (TFA), 117.74 (TFA), 119.71 (TFA), 127.61, 130.12 (2 carbon atoms), 153.73, 153.91, 155.76, 156.81, 158.70 (q, *J* 32 Hz) (TFA), 168.75, 169.46, 170.48, 170.62, 170.90, 171.22, 171.26, 171.44, 173.82. HRMS (ESI): *m*/*z* [M + 4H]^4+^ calcd for [C_60_H_101_N_17_O_12_]^4+^ 312.9449, found: 312.9459. RP-HPLC (220 nm): >99% (*t*_R_ = 8.1 min, *k* = 9.7). C_60_H_97_N_17_O_12_·C_8_H_4_F_12_O_8_ (1248.54 + 456.09).

#### *N*^α^-(Pent-4-ynoyl)-Lys-Pro-Arg-Arg-Pro-Tyr-Ile-Leu Tris(hydrotrifluoroacetate) (**7**)

Peptide **7** was synthesized on a H-Leu-2-ClTrt resin (60 mg, 0.79 mmol/g) according to the general procedure. Purification by preparative HPLC (gradient: 0–30 min: acetonitrile/0.1% aqueous TFA 15:85–35:65, *t*_R_ = 11 min) yielded **7** as a white fluffy solid (26.1 mg, 38%). ^1^H NMR (600 MHz, DMSO-*d*_6_): δ (ppm) 0.80 (t, 3H, *J* 7.5 Hz), 0.83 (d, 6H, *J* 6.5 Hz), 0.89 (d, 3H, *J* 6.6 Hz), 1.01–1.09 (m, 1H), 1.30–1.72 (m, 19H), 1.73–1.93 (m, 6H), 1.93–2.07 (m, 2H), 2.24–2.40 (m, 4H), 2.65–2.71 (m, 1H), 2.71–2.79 (m, 3H), 2.82–2.89 (m, 1H), 3.01–3.15 (m, 4H), 3.46–3.54 (m, 2H), 3.55–3.61 (m, 1H), 3.63–3.69 (m, 1H), 4.18–4.24 (m, 3H), 4.30–4.36 (m, 2H), 4.38–4.43 (m, 1H), 4.43–4.51 (m, 2H), 6.58–6.63 (m, 2H), 6.68–7.20 (br s, 4H, interfering with next listed signal), 6.98–7.01 (m, 2H), 7.20–7.59 (br s, 4H), 7.64 (t, 1H, *J* 5.5 Hz), 7.68 (t, 1H, *J* 5.7 Hz), 7.71–7.83 (m, 4H), 7.89 (d, 1H, *J* 8.0 Hz), 7.95 (d, 1H, *J* 7.5 Hz), 8.06 (d, 1H, *J* 8.0 Hz), 8.14 (d, 1H, *J* 8.0 Hz), 8.16–8.21 (d, 1H, *J* 7.9 Hz), 9.20 (s, 1H), 12.52 (s, 1H). ^13^C NMR (150 MHz, DMSO-*d*_6_): δ (ppm) 10.91, 14.14, 15.12, 21.21, 21.96, 22.84, 24.10, 24.17, 24.26, 24.46, 25.03, 26.65, 28.38, 28.92, 29.07, 29.12, 30.56 (2 carbon atoms), 33.75, 36.37, 37.12, 38.67, 39.78, 40.37, 40.49, 46.73, 46.87, 49.84, 50.03, 50.13, 52.00, 54.13, 56.35, 59.16, 59.24, 71.29, 83.72, 113.86 (TFA), 114.77 (2 carbon atoms), 115.83 (TFA), 117.81 (TFA), 119.78 (TFA), 127.62, 130.12 (2 carbon atoms), 155.75, 156.78 (2 carbon atoms), 158.51 (q, *J* 32 Hz) (TFA), 169.46, 170.04, 170.22, 170.64, 170.90, 171.19, 171.28, 171.55, 173.82. HRMS (ESI): *m*/*z* [M + 3H]^3+^ calcd for [C_54_H_90_N_15_O_11_]^3+^ 374.8976, found: 374.8982. RP-HPLC (220 nm): >99% (*t*_R_ = 8.8 min, *k* = 10.6). C_54_H_87_N_15_O_11_·C_6_H_3_F_9_O_6_ (1122.38 + 342.07).

#### *N*^α^-(Pent-4-ynoyl)-d-Lys-Pro-Arg-Arg-Pro-Tyr-Ile-Leu Tris(hydrotrifluoroacetate) (**8**)

Peptide **8** was synthesized on a H-Leu-2-ClTrt resin (60 mg, 0.79 mmol/g) according to the general procedure. Purification by preparative HPLC (gradient: 0–30: min acetonitrile/0.1% aqueous TFA 15:85–35:65, *t*_R_ = 11 min) yielded **8** as a white fluffy solid (7.1 mg, 10%). ^1^H NMR (600 MHz, DMSO-*d*_6_): δ (ppm) 0.80 (t, 3H, *J* 7.5 Hz), 0.84 (d, 6H, *J* 6.5 Hz), 0.90 (d, 3H, *J* 6.6 Hz), 1.01–1.09 (m, 1H), 1.21–1.31 (m, 1H), 1.31–1.38 (m, 1H), 1.38–1.59 (m, 13H), 1.59–1.72 (m, 5H), 1.75–1.90 (m, 5H), 1.93–2.06 (m, 2H), 2.26–2.42 (m, 4H), 2.65–2.72 (m, 1H), 2.72–2.79 (m, 3H), 2.84–2.90 (m, 1H), 3.01–3.14 (m, 4H), 3.28–3.41 (m, 1H), 3.46–3.54 (m, 1H, overlaying with the water signal, identified in the spectrum acquired after the addition of D_2_O), 3.54–3.61 (m, 1H, overlaying with the water signal, identified in the spectrum acquired after the addition of D_2_O), 3.69–3.75 (m, 1H, overlaying with the water signal, identified in the spectrum acquired after the addition of D_2_O), 4.15–4.24 (m, 3H), 4.28–4.36 (m, 2H), 4.36–4.51 (m, 3H), 6.59–6.63 (m, 2H), 6.68–7.17 (br s, 4H, interfering with next listed signal), 6.97–7.00 (m, 2H), 7.17–7.54 (br s, 4H), 7.56–7.62 (m, 1H), 7.67 (t, 1H, *J* 5.8 Hz), 7.69–7.79 (m, 4H), 7.82 (d, 1H, *J* 8.0 Hz), 7.85–7.93 (m, 2H), 8.16–8.21 (m, 1H), 8.24 (d, 1H, *J* 7.2 Hz), 9.20 (s, 1H), 12.51 (s, 1H). ^13^C NMR (150 MHz, DMSO-*d*_6_): δ (ppm) 10.91, 14.17, 15.12, 21.22, 22.03, 22.84, 24.09, 24.17, 24.26, 24.47, 25.13, 26.75, 28.24, 28.60, 29.05, 29.09, 30.56 (2 carbon atoms), 33.73, 36.37, 37.11, 38.64, 39.78, 40.38, 40.51, 46.70, 46.85, 49.96, 50.13, 50.42, 52.08, 54.12, 56.36, 59.21, 59.59, 71.33, 83.68, 113.96 (TFA), 114.78 (2 carbon atoms), 115.94 (TFA), 117.91 (TFA), 119.89 (TFA), 127.63, 130.11 (2 carbon atoms), 155.75, 156.76 (2 carbon atoms), 158.21 (q, *J* 32 Hz) (TFA), 169.64, 170.34, 170.64, 170.69, 170.90, 171.22, 171.28, 171.32, 172.08. HRMS (ESI): *m*/*z* [M + 3H]^3+^ calcd for [C_54_H_90_N_15_O_11_]^3+^ 374.8976, found: 374.8986. RP-HPLC (220 nm): 99% (*t*_R_ = 8.9 min, *k* = 10.7). C_54_H_87_N_15_O_11_·C_6_H_3_F_9_O_6_ (1122.38 + 342.07).

#### *N*^α^-(4-Aminobutanoyl)-2-(prop-2-ynyl)-Gly-2-(piperidin-4-yl)-Gly-Pro-Arg-Arg-Pro-Tyr-Ile-Leu Tetrakis(hydrotrifluoroacetate) (**9**)

Peptide **9** was synthesized on a H-Leu-2-ClTrt resin (60 mg, 0.79 mmol/g) according to the general procedure. Purification by preparative HPLC (gradient: 0–30 min: acetonitrile/0.1% aqueous TFA 18:82–38:62, *t*_R_ = 10 min) yielded **9** as a white fluffy solid (16.1 mg, 20%). ^1^H NMR (600 MHz, DMSO-*d*_6_): δ (ppm) 0.73–0.90 (m, 12H), 1.02–1.12 (m, 1H), 1.19–1.65 (m, 13H), 1.65–1.92 (m, 13H), 1.92–1.98 (m, 1H), 1.98–2.09 (m, 1H), 2.18–2.31 (m, 2H), 2.37–2.58 (m, 2H, interfering with the solvent signal, identified via HSQC and HMBC), 2.72–2.84 (m, 5H), 2.88–3.15 (m, 6H), 3.17–3.28 (m, 2H, interfering with the water signal, quantified in the spectrum acquired after the addition of D_2_O), 3.47–3.55 (m, 2H), 3.55–3.62 (m, 1H), 3.62–3.70 (m, 1H), 3.97–4.11 (m, 1H), 4.11–4.31 (m, 3H), 4.31–4.43 (m, 3H), 4.43–4.55 (m, 2H), 5.90–7.55 (br s, 8H, interfering with next two listed signals), 6.57–6.66 (m, 2H), 6.96–7.02 (m, 2H), 7.55–8.99 (m, 14H), 9.23 (br s, 1H). The proton signal of the carboxylic acid group was not apparent. ^13^C NMR (150 MHz, DMSO-*d*_6_): δ (ppm) 10.98, 15.27, 21.88, 22.24, 22.94, 23.34, 24.06, 24.27, 24.39, 24.46 (2 carbon atoms), 24.59, 24.72, 25.42, 28.25, 28.43, 28.78, 29.28, 31.83, 35.70, 36.59 (2 carbon atoms), 38.46, 40.13 (interfering with the solvent signal, identified by HSQC), 40.42, 41.98 (identified by HSQC), 42.91, 43.13, 46.76, 47.18, 49.89, 51.31, 52.09, 52.69, 53.89, 57.35, 59.45 (2 carbon atoms), 59.65, 72.86, 80.48, 114.75 (2 carbon atoms), 116.22 (TFA), 118.22 (TFA), 127.98, 129.99 (2 carbon atoms), 155.75, 157.02, 157.08, 158.25 (TFA), 158.43 (TFA), 168.46, 169.87, 169.98, 170.05, 170.88, 171.01, 171.30, 171.36 (2 carbon atoms), 176.22. HRMS (ESI): *m*/*z* [M + 4H]^4+^ calcd for [C_59_H_99_N_17_O_12_]^4+^ 309.4409, found: 309.4420. RP-HPLC (220 nm): 98% (*t*_R_ = 7.7 min, *k* = 9.1). C_59_H_95_N_17_O_12_·C_8_H_4_F_12_O_8_ (1234.52 + 456.09).

#### *N*^α^-(Pent-4-ynoyl)-2-(piperidin-4-yl)-Gly-Pro-*N*^ω^-[(4-aminobutyl)aminocarbonyl]Arg-Arg-Pro-β,β-dimethyl-l-Tyr-Ile-Leu Tetrakis(hydrotrifluoroacetate) (**10a**) and *N*^α^-(Pent-4-ynoyl)-2-(piperidin-4-yl)-Gly-Pro-*N*^ω^-[(4-aminobutyl)aminocarbonyl]Arg-Arg-Pro-β,β-dimethyl-d-Tyr-Ile-Leu Tetrakis(hydrotrifluoroacetate) (**10b**)

Peptides **10a** and **10b**, representing diastereomers, were synthesized on a H-Leu-2-ClTrt resin (130 mg, 0.79 mmol/g) according to the general procedure. The unavailability of enantiomerically pure Fmoc-β,β-dimethyl-l-Tyr(tBu)–OH (this Fmoc amino acid was only available as racemic mixture) necessitated the synthesis of both diastereomers, which were separated by preparative HPLC (gradient: 0–40 min: acetonitrile/0.1% aqueous TFA 15:85–40:60, *t*_R_ (**10a**) = 17 min, *t*_R_ (**10b**) = 20 min). Lyophilization of the eluates yielded **10a** and **10b** as white fluffy solids (**10a**: 60.3 mg, 34%; **10b**: 37.2 mg, 21%). ^1^H NMR of **10a** (600 MHz, DMSO-*d*_6_): δ (ppm) 0.78 (t, 3H, *J* 7.3 Hz), 0.82 (t, 6H, *J* 6.9 Hz), 0.90 (d, 3H, *J* 6.6 Hz), 0.96–1.05 (m, 1H), 1.23 (s, 3H), 1.25 (s, 3H), 1.31–1.44 (m, 3H), 1.44–1.60 (m, 12H), 1.60–1.99 (m, 14H), 2.02–2.11 (m, 1H), 2.28–2.40 (m, 4H), 2.73–2.89 (m, 5H), 3.03–3.14 (m, 4H), 3.19–3.27 (m, 3H), 3.27–3.32 (m, 1H), 3.38–3.46 (br s, 1H, quantified in the spectrum acquired after the addition of D_2_O), 3.51–3.57 (m, 2H, quantified in the spectrum acquired after the addition of D_2_O), 3.67–3.74 (m, 1H), 4.08–4.14 (m, 1H), 4.19–4.27 (m, 2H), 4.29–4.34 (m, 1H), 4.35–4.40 (m, 1H), 4.40–4.48 (m, 2H), 4.60–4.65 (m, 1H), 6.58–6.65 (m, 2H), 6.85–7.20 (br s, 2H, interfering with next listed signal), 7.09–7.14 (m, 2H), 7.21–7.50 (m, 3H), 7.55–7.66 (m, 2H), 7.68 (t, 1H, *J* 5.4 Hz), 7.80 (s, 3H), 7.98 (d, 1H, *J* 7.4 Hz), 8.04–8.15 (m, 2H), 8.19 (d, 1H, *J* 8.7 Hz), 8.33–8.42 (m, 1H), 8.42–8.52 (br s, 2H), 8.70–8.81 (m, 1H), 9.06 (s, 1H), 9.17 (s, 1H), 10.67 (s, 1H), 12.49 (s, 1H). ^13^C NMR of **10a** (150 MHz, DMSO-*d*_6_): δ (ppm) 10.85, 14.22, 15.11, 21.09, 22.95, 24.16–24.55 (signal cluster of 8 carbon atoms, identified via HSQC), 25.04, 25.95, 26.97, 28.41 (2 carbon atoms), 28.52, 28.76, 29.35, 33.73, 35.49, 36.67, 38.47, 38.64, 39.60 (interfering with the solvent signal, identified by HSQC), 40.17, 40.46, 40.51, 42.74, 43.00, 46.72, 47.27, 49.80, 49.97, 51.98, 53.65, 56.60, 59.25, 59.43, 59.77, 71.36, 83.67, 114.05 (TFA), 114.26 (2 carbon atoms), 116.03 (TFA), 118.00 (TFA), 119.99 (TFA), 127.44 (2 carbon atoms), 136.22, 153.74, 153.90, 155.27, 156.82, 158.71 (q, *J* 32 Hz) (TFA), 168.72, 169.35, 169.84, 170.47, 170.59, 170.74, 171.26, 171.44, 173.85. HRMS (ESI): *m*/*z* [M + 4H]^4+^ calcd for [C_62_H_105_N_17_O_12_]^4+^ 319.9527, found: 319.9537 (**10a**); 319.9542 (**10b**). RP-HPLC (220 nm): **10a**: 97% (*t*_R_ = 8.9 min, *k* = 10.7), **10b**: 98% (*t*_R_ = 10.4 min, *k* = 12.7). C_62_H_101_N_17_O_12_·C_8_H_4_F_12_O_8_ (1276.60 + 456.09).

#### *N*^α^-(3-(1-*N*-((1-Carboxylato-2-(6-(dimethylamino)-3-(dimethyliminio)-3*H*-xanthen-9-yl)phen-5-yl)1-oxomethyl)aminoprop-3-yl-1*H*-1,2,3-triazol-4-yl)propanoyl)-2-(piperidin-4-yl)-Gly-Pro-*N*^ω^-[(4-(*N*-propanoyl)aminobutyl)aminocarbonyl]Arg-Arg-Pro-Tyr-Ile-Leu Tris(hydrotrifluoroacetate) (**13**)

Peptide **6** (6.2 mg, 3.9 μmol) was dissolved in anhydrous DMF (100 μL) and a solution of succinimidyl propionate (**11**) (0.40 mg, 2.3 μmol) and DIPEA (35 μmol, 6.1 μL) in anhydrous DMF (30 μL) was added. The mixture was stirred at rt for 2 h. 10% aqueous TFA (30 μL) and 0.1% aqueous TFA/acetonitrile 85:15 v/v (1000 μL) were added and the product of the propionylation reaction was isolated by preparative RP-HPLC (gradient: 0–30 min: acetonitrile/0.1% aqueous TFA 20:80–40:60, *t*_R_ = 14 min). Lyophilization of the eluate yielded a white fluffy solid (2.9 mg, 45%). The propionylation product (2.9 mg, 1.8 μmol) and azide **12** (1.3 mg, 2.5 μmol) were dissolved in NMP/PBS 1:1 v/v (180 μL). A solution of CuSO_4_·5H_2_O (0.77 mg, 3.1 μmol) in NMP/PBS 1:1 v/v (60 μL) (note: CuSO_4_ was first dissolved in 30 μL of PBS and then 30 μL of NMP were added) and a solution of sodium ascorbate (1.8 mg, 8.9 μmol) in PBS (30 μL) were added. After stirring at rt in the dark for 2 h, the mixture was diluted with 800 μL of 0.1% aqueous TFA/acetonitrile 85:15 v/v followed by isolation of the product by preparative HPLC (gradient: 0–40 min: acetonitrile/0.1% aqueous TFA 25:75–55:45, *t*_R_ = 11 min). Lyophilization of the eluate afforded **13** as a purple fluffy solid (2.95 mg, 77%). ^1^H NMR (600 MHz, DMSO-*d*_6_): δ (ppm) 0.79 (t, 3H, *J* 7.4 Hz), 0.85 (d, 6H, *J* 6.6 Hz), 0.90 (d, 3H, *J* 6.6 Hz), 0.97 (t, 3H, *J* 7.5 Hz), 1.01–1.08 (m, 1H), 1.20–1.44 (m, 7H), 1.44–1.58 (m, 8H), 1.58–1.74 (m, 5H), 1.74–1.85 (m, 5H), 1.85–2.01 (m, 4H), 2.02–2.08 (m, 3H), 2.08–2.15 (m, 2H), 2.45–2.57 (m, 3H, overlaying with the solvent signal, identified by ^1^H-COSY), 2.63–2.70 (m, 1H, D_2_O), 2.70–2.89 (m, 5H, D_2_O), 2.95–3.08 (m, 6H, D_2_O), 3.11–3.28 (m, 15H, D_2_O), 3.29–3.37 (m, 2H, D_2_O), 3.40–3.48 (m, 1H, D_2_O), 3.48–3.58 (m, 2H, D_2_O), 3.64–3.72 (m, 1H, D_2_O), 4.18–4.27 (m, 3H), 4.30–4.36 (m, 2H), 4.34–4.50 (m, 5H), 6.39–6.57 (br s, 1H), 6.58–6.63 (m, 2H),6.63–6.88 (br s, 2H), 6.89–7.13 (m, 7H), 7.16–7.43, (br s, 2H), 7.14–7.66 (m, 3H), 7.66–7.79 (m, 2H), 7.84–7.93 (m, 2H), 7.97 (d, 1H, *J* 7.4 Hz), 8.07 (d, 1H, *J* 7.5 Hz), 8.15–8.22 (m, 2H), 8.22–8.32 (br s, 2H), 8.32–8.50 (br s, 2H), 8.54–8.78 (m, 2H), 8.88–9.12 (m, 2H), 9.17 (s, 1H), 10.10 (s, 1H), 12.51 (s, 1H), 13.41 (s, 1H). HRMS (ESI): *m*/*z* [M + 3H]^3+^ calcd for [C_91_H_132_N_23_O_17_]^3+^ 606.3385, found: 606.3392. RP-HPLC (220 nm): 99% (*t*_R_ = 7.0 min, *k* = 8.2). C_91_H_129_N_23_O_17_·C_6_H_3_F_9_O_6_ (1817.18 + 342.07).

#### *N*^α^-(3-(1-*N*-((1-Carboxylato-2-(6-(dimethylamino)-3-(dimethyliminio)-3*H*-xanthen-9-yl)phen-5-yl)1-oxomethyl)aminoprop-3-yl-1*H*-1,2,3-triazol-4-yl)propanoyl)-*N*^ω^-(propanoyl)-Lys-Pro-Arg-Arg-Pro-Tyr-Ile-Leu Bis(hydrotrifluoroacetate) (**14**)

Peptide **7** (8.9 mg, 6.1 μmol) was dissolved in anhydrous DMF (100 μL) and a solution of **11** (0.62 mg, 3.7 μmol) and DIPEA (55 μmol, 10.5 μL) in anhydrous DMF (30 μL) was added. The mixture was stirred at rt for 2 h. 10% aqueous TFA (30 μL) and 0.1% aqueous TFA/acetonitrile 85:15 v/v (1000 μL) were added and the product of the propionylation reaction was isolated by preparative RP-HPLC (gradient: 0–30 min: acetonitrile/0.1% aqueous TFA 18:82–38:62, *t*_R_ = 15 min). Lyophilization of the eluate afforded a white fluffy solid (4.2 mg, 49%). The propionylation product (2.1 mg, 1.5 μmol) and azide **12** (1.0 mg, 2.0 μmol) were dissolved in NMP/PBS 1:1 v/v (180 μL). A solution of CuSO_4_·5H_2_O (0.62 mg, 2.5 μmol) in NMP/PBS 1:1 v/v (60 μL) (note: CuSO_4_ was first dissolved in 30 μL of PBS and then 30 μL of NMP were added) and a solution of sodium ascorbate (1.4 mg, 7.1 μmol) in PBS (30 μL) were added. After stirring at rt in the dark for 2 h, the mixture was diluted with 800 μL of 0.1% aqueous TFA/acetonitrile 85:15 v/v followed by isolation of the product by preparative HPLC (gradient: 0–40 min: acetonitrile/0.1% aqueous TFA 25:75–55:45, *t*_R_ = 13 min). Lyophilization of the eluate afforded **14** as a purple fluffy solid (2.9 mg, 99%). HRMS (ESI): *m*/*z* [M + 2H]^2+^ calcd for [C_85_H_121_N_21_O_16_]^2+^ 845.9645, found: 845.9653. RP-HPLC (220 nm): >99% (*t*_R_ = 7.9 min, *k* = 9.4). C_85_H_119_N_21_O_16_·C_4_H_2_F_6_O_4_ (1691.02 + 228.05).

#### *N*^α^-(3-(1-*N*-((1-Carboxylato-2-(6-(dimethylamino)-3-(dimethyliminio)-3*H*-xanthen-9-yl)phen-5-yl)1-oxomethyl)aminoprop-3-yl-1*H*-1,2,3-triazol-4-yl)propanoyl)-*N*^ω^-(propanoyl)-d-Lys-Pro-Arg-Arg-Pro-Tyr-Ile-Leu Bis(hydrotrifluoroacetate) (**15**)

Compound **15** was prepared from peptide **8** (4.5 mg, 3.1 μmol), **11** (0.32 mg, 1.8 μmol) and **12** (1.5 mg, 2.9 μmol) according to the procedure used for the synthesis of **14**. The product of the propionylation reaction, resulting from the treatment of **8** with **11**, was obtained as a white fluffy solid (3.1 mg, 2.2 μmol, 71%). Peptide **15**, resulting from the “click” reaction between the propionylation product and **12**, was obtained as a purple fluffy solid (2.61 mg, 61%). HRMS (ESI): *m*/*z* [M + 2H]^2+^ calcd for [C_85_H_121_N_21_O_16_]^2+^ 845.9645, found: 845.9655. RP-HPLC (220 nm): >99% (*t*_R_ = 7.8 min, *k* = 9.3). C_85_H_119_N_21_O_16_·C_4_H_2_F_6_O_4_ (1691.02 + 228.05).

#### *N*^α^-[(4-(*N*-Propanoyl)aminobutanoyl)]-2-(1-(1-*N*-((1-carboxylato-2-(6-(dimethylamino)-3-(dimethyliminio)-3*H*-xanthen-9-yl)phen-5-yl)1-oxomethyl)aminoprop-3-yl-1*H*-1,2,3-triazol-4-yl)methyl)-Gly-2-(piperidin-4-yl)-Gly-Pro-Arg-Arg-Pro-Tyr-Ile-Leu Tris(hydrotrifluoroacetate) (**16**)

Peptide **9** (5.0 mg, 3.0 μmol) was dissolved in anhydrous DMF (100 μL) and a solution of **11** (0.36 mg, 2.1 μmol) and DIPEA (32 μmol, 5.5 μL) in anhydrous DMF (30 μL) was added. The mixture was stirred at rt for 2 h. 10% aqueous TFA (30 μL) and 0.1% aqueous TFA/acetonitrile 85:15 v/v (1000 μL) were added and the product of the propionylation reaction was isolated by preparative RP-HPLC (gradient: 0–30 min: acetonitrile/0.1% aqueous TFA 20:80–40:60, *t*_R_ = 14 min). Lyophilization of the eluate yielded a white fluffy (1.5 mg, 31%). The propionylation product (1.5 mg, 0.92 μmol) and **12** (0.66 mg, 1.3 μmol) were dissolved in NMP/PBS 1:1 v/v (180 μL). A solution of CuSO_4_·5H_2_O (0.41 mg, 1.6 μmol) in NMP/PBS 1:1 v/v (60 μL) (note: CuSO_4_ was first dissolved in 30 μL of PBS and then 30 μL of NMP were added) and a solution of sodium ascorbate (0.92 mg, 4.6 μmol) in PBS (30 μL) were added. After stirring at rt in the dark for 2 h, the mixture was diluted with 800 μL of 0.1% aqueous TFA/acetonitrile 85:15 v/v and the product was isolated by preparative HPLC (gradient: 0–40 min: acetonitrile/0.1% aqueous TFA 25:75–55:45, *t*_R_ = 10 min). Lyophilization of the eluate afforded **16** as a purple fluffy solid (0.78 mg, 40%). HRMS (ESI): *m*/*z* [M + 3H]^3+^ calcd for [C_90_H_130_N_23_O_17_]^3+^ 601.6666, found: 601.6675. RP-HPLC (220 nm): >99% (*t*_R_ = 6.4 min, *k* = 7.4). C_90_H_127_N_23_O_17_·C_6_H_3_F_9_O_6_ (1803.15 + 342.07).

#### 24-(5-(3-Azidopropyl)amino-5-oxopent-1-yn-1-yl)-5,5,27,27-tetramethyl-16-oxa-20-aza-12-azoniaheptacyclo-[15.11.0.03,15.04,12.06,11.020,28.021,26]octacosa-1(28),2,4(12),6-(11),7,9,21(26),22,24-nonaene-8-sulfonate (**17**)

Compound **21** (15.0 mg, 19.2 μmol) was dissolved in anhydrous DMF (600 μL). DIPEA (10 μL, 58 μmol) and a solution of 3-azidopropylamine (6.2 mg, 29 μmol) in anhydrous DMF (80 μL) were added and the mixture was stirred at room temperature in the dark for 1 h. 10% aqueous TFA (30 μL) and 0.1% aqueous TFA/acetonitrile 85:15 v/v (700 μL) were added, and the solution was subjected to preparative RP-HPLC (Kinetex-XB C18 column, mobile phase: 0–40 min: 0.1% aqueous TFA/acetonitrile 80:20 to 50:50, *t*_R_ = 24 min), yielding **17** as a purple fluffy solid (5.8 mg, 54%). HRMS (ESI): *m*/*z* [M + 3H]^3+^ calcd for [C_37_H_41_N_6_O_5_S]^+^ 681.2854, found: 681.2867. RP-HPLC (220 nm): >98% (*t*_R_ = 5.7 min, *k* = 6.5). C_37_H_40_N_6_O_5_S (680.82).

#### *N*^α^-(3-(*N*-(1-(8-Sulfonato-5,5,27,27-tetramethyl-16-oxa-20-aza-12-azoniaheptacyclo[15.11.0.03,15.04,12.06,11.020,28.021,26]octacosa-1(28),2,4(12),6(11),7,9,21(26),22,24-nonaen-24-yl)-5-oxopentyn-5-yl)-1-aminoprop-3-yl-1*H*-1,2,3-triazol-4-yl)propanoyl)-2-(piperidin-4-yl)-Gly-Pro-*N*^ω^-[(4-(*N*-propanoyl)aminobutyl)aminocarbonyl]Arg-Arg-Pro-β,β-dimethyl-l-Tyr-Ile-Leu Tris(hydrotrifluoroacetate) (**18**)

Peptide **10a** (16.3 mg, 9.4 μmol) was dissolved in anhydrous DMF (170 μL) and a solution of **11** (1.0 mg, 6.0 μmol) and DIPEA (91 μmol, 15.8 μL) in anhydrous DMF (30 μL) was added. The mixture was stirred at rt for 2 h. 10% aqueous TFA (30 μL) and 0.1% aqueous TFA/acetonitrile 85:15 v/v (1000 μL) were added and the product of the propionylation reaction was isolated by preparative RP-HPLC (gradient: 0–40 min: acetonitrile/0.1% aqueous TFA 18:82–43:57, *t*_R_ = 17 min). Lyophilization of the eluate yielded a white fluffy solid (6.0 mg, 38%). The propionylation product (2.3 mg, 1.4 μmol) and azide **17** (1.2 mg, 1.7 μmol) were dissolved in NMP/PBS 1:1 v/v (180 μL). A solution of CuSO_4_·5H_2_O (0.60 mg, 2.4 μmol) in NMP/PBS 1:1 v/v (60 μL) (note: CuSO_4_ was first dissolved in 30 μL of PBS and then 30 μL of NMP were added) and a solution of sodium ascorbate (1.4 mg, 6.9 μmol) in PBS (30 μL) were added. After stirring at rt in the dark for 2 h, the mixture was diluted with 800 μL of 0.1% aqueous TFA/acetonitrile 85:15 v/v followed by purification of the product by preparative HPLC (gradient: 0–40 min: acetonitrile/0.1% aqueous TFA 20:80–50:50, *t*_R_ = 19 min) affording **18** as a purple fluffy solid (1.3 mg, 39%). ^1^H NMR (600 MHz, DMSO-*d*_6_): δ (ppm) 0.78 (t, 3H, *J* 7.4 Hz), 0.83 (t, 6H, *J* 7.7 Hz), 0.90 (d, 3H, *J* 6.6 Hz), 0.97 (t, 3H, *J* 7.7 Hz), 0.99–1.05 (m, 1H), 1.19–1.32 (m, 7H), 1.32–1.45 (m, 6H), 1.45–1.61 (m, 15H), 1.61–1.82 (m, 17H), 1.82–1.99 (m, 8H), 1.99–2.07 (m, 3H), 2.37–2.39 (m, 2H), 2.52–2.58 (m, 2H), 2.68 (t, 2H, *J* 7.1 Hz), 2.72–2.91 (m, 5H), 2.99–3.04 (m, 2H), 3.04–3.17 (m, 7H), 3.20–3.45 (3H, overlaying with the water signal, identified and quantified in the spectrum acquired after the addition of D_2_O), 3.53–3.60 (m, 2H), 3.64–3.72 (m, 1H), 3.83–3.97 (m, 2H), 4.09–4.14 (m, 1H), 4.19–4.27 (m, 2H), 4.27–4.40 (m, 6H), 4.44–4.54 (m, 2H), 4.60–4.68 (m, 3H), 6.52 (s, 2H), 6.58–6.64 (m, 2H), 6.68–7.04 (br s, 2H), 7.09–7.15 (m, 2H), 7.15–7.41 (m, 5H), 7.41–7.47 (m, 2H), 7.48–7.54 (m, 2H), 7.58–7.63 (d, 1H, *J* 8.3 Hz), 7.70–7.77 (m, 2H), 7.85 (s, 1H), 7.88 (s, 1H), 7.95–8.02 (m, 2H), 8.06–8.14 (m, 3H), 8.15–8.27 (m, 2H), 8.32–8.45 (br s, 1H), 8.52–8.63 (br s, 1H), 8.93–9.04 (br s, 1H), 9.13 (s, 1H), 9.84 (br s, 1H), 12.47 (br s, 1H). HRMS (ESI): *m*/*z* [M + 3H]^3+^ calcd for [C_102_H_148_N_23_O_18_S]^3+^ 672.0370, found: 672.0381. RP-HPLC (220 nm): >99% (*t*_R_ = 7.7 min, *k* = 9.1). C_102_H_145_N_23_O_18_S·C_6_H_3_F_9_O_6_ (2013.49 + 342.07).

#### *N*^α^-(3-(1-*N*-((1-Carboxy-2-(6-(dimethylamino)-3-(dimethyliminio)-3*H*-xanthen-9-yl)phen-5-yl)1-oxomethyl)aminoprop-3-yl-1*H*-1,2,3-triazol-4-yl)propanoyl)-2-(piperidin-4-yl)-Gly-Pro-*N*^ω^-[(4-aminobutyl)aminocarbonyl]Arg-Arg-Pro-Tyr-Ile-Leu Tetrakis(hydrotrifluoroacetate) (**19**)

Peptide **6** (1.5 mg, 0.88 μmol) and azide **12** (0.68 mg, 1.3 μmol) were dissolved in NMP/PBS 1:1 v/v (140 μL). A solution of CuSO_4_·5H_2_O (0.42 mg, 1.7 μmol) in NMP/PBS 1:1 v/v (50 μL) (note: CuSO_4_ was first dissolved in 25 μL of PBS and then 25 μL of NMP were added) and a solution of sodium ascorbate (0.95 mg, 4.8 μmol) in PBS (25 μL) were added. After stirring at rt in the dark for 2 h, the mixture was diluted with 0.1% aqueous TFA/acetonitrile 85:15 v/v (800 μL) followed by isolation of the product by preparative HPLC (gradient: 0–40 min: acetonitrile/0.1% aqueous TFA 18:82–48:52, *t*_R_ = 17 min). Lyophilization of the eluate afforded **19** as a purple fluffy solid (1.2 mg, 61%). HRMS (ESI): *m*/*z* [M + 4H]^4+^ calcd for [C_88_H_129_N_23_O_16_]^4+^ 440.9992, found: 441.0005. RP-HPLC (220 nm): 97% (*t*_R_ = 12.5 min, *k* = 15.4). C_88_H_125_N_23_O_16_·C_8_H_4_F_12_O_8_ (1765.14 + 456.09).

#### *N*^α^-(3-(*N*-(1-(8-Sulfonato-5,5,27,27-tetramethyl-16-oxa-20-aza-12-azoniaheptacyclo[15.11.0.03,15.04,12.06,11.020,28.021,26]octacosa-1(28),2,4(12),6(11),7,9,21(26),22,24-nonaen-24-yl)-5-oxopentyn-5-yl)-1-aminoprop-3-yl-1*H*-1,2,3-triazol-4-yl)propanoyl)-2-(piperidin-4-yl)-Gly-Pro-*N*^ω^-[(4-aminobutyl)aminocarbonyl]Arg-Arg-Pro-β,β-dimethyl-l-Tyr-Ile-Leu-OH Tetrakis(hydrotrifluoroacetate) (**20**)

Peptide **10a** (6.7 mg, 3.9 μmol) and azide **17** (3.4 mg, 5.0 μmol) were dissolved in NMP/PBS 1:1 v/v (300 μL). A solution of CuSO_4_·5H_2_O (1.8 mg, 7.4 μmol) in NMP/PBS 1:1 v/v (100 μL) (note: CuSO_4_ was first dissolved in 50 μL of PBS and then 50 μL of NMP were added) and a solution of sodium ascorbate (4.2 mg, 21 μmol) in PBS (50 μL) were added. After stirring at rt in the dark for 2 h, the mixture was diluted with 0.1% aqueous TFA/acetonitrile 85:15 v/v (800 μL) followed by isolation of the product by preparative HPLC (gradient: 0–40 min: acetonitrile/0.1% aqueous TFA 18:82–48:52, *t*_R_ = 18 min). Lyophilization of the eluate afforded **20** as a purple fluffy solid (3.2 mg, 34%). HRMS (ESI): *m*/*z* [M + 4H]^4+^ calcd for [C_99_H_145_N_23_O_17_S]^3+^ 490.2730, found: 490.2745. RP-HPLC (220 nm): 99% (*t*_R_ = 13.3 min, *k* = 16.5). C_99_H_141_N_23_O_17_S·C_8_H_4_F_12_O_8_ (1957.42 + 456.09).

### Synthesis of [^3^H]**13** and [^3^H]**18**

A solution of succinimidyl [^3^H]propionate (molar activity: 3.89 TBq/mmol, purchased from Novandi, Södertälje, Sweden) (55.5 MBq, 0.75 mL, 14.3 nmol) in *n*-heptane/EtOAc 3:2 v/v was transferred from the delivered ampule into a 1.5 mL polypropylene reaction vessel with screw cap, and the solvent was removed in a vacuum concentrator (30 °C, ca. 30 min). A solution of the precursor peptide **19** (0.35 mg, 166.4 nmol) or **20** (0.35 mg, 152.2 nmol) and DIPEA (1 μL, 5.73 μmol) in DMF/NMP 75:25 v/v (55 μL) was added, immediately followed by vortexing for 20 s. Subsequently, the vessel was shaken at rt in the dark for 2 h. The mixture was acidified by the addition of 2% aqueous TFA (32 μL), followed by the addition of acetonitrile/H_2_O 1:9 v/v (280 μL). [^3^H]**13** and [^3^H]**18** were isolated using a HPLC system from Waters (Eschborn, Germany) consisting of two pumps 510, a pump control module, a manual injector (loop size: 200 μL), a 486 UV/vis detector, and a Flow-one Beta A-500 radiodetector (Packard, Meriden) (the latter was disconnected during the purification process). A Luna C18(2) column (3 μm, 150 mm × 4.6 mm, Phenomenex, Aschaffenburg, Germany) was used as stationary phase at a flow rate of 0.8 mL/min. Mixtures of acetonitrile supplemented with 0.04% TFA (A) and 0.05% aqueous TFA (B) were used as mobile phase. The following linear gradient was applied: 0–20 min: A/B 10:90–44:56, 20–25 min: 44:56–95:5, 25–30 min: 95:5 (isocratic). Four ([^3^H]**13**) and three ([^3^H]**18**) injections were applied. The fractions containing [^3^H]**13** (*t*_R_ = 17.7 min) or [^3^H]**18** (*t*_R_ = 18.0 min) were collected and combined in a 2 mL polypropylene reaction vessel with screw cap. The volume of the combined eluates was reduced by evaporation in a vacuum concentrator to approximately 300 μL. Ethanol (450 μL) was added resulting in a mixing ratio of EtOH/H_2_O 6:4 v/v and a total volume of 750 μL (preliminary stock solution). To determine the radiochemical purity and to prove the identity of [^3^H]**13** and [^3^H]**18**, 2 μL of the preliminary stock solution were added to 100 μL of a 15 μM solution of **13** (“cold” analogue of [^3^H]**13**) or **18** (“cold” analogue of [^3^H]**18**) in A/B 1:9 affording the sample to be analyzed using the aforementioned HPLC system, column and solvents. The following linear gradient was applied: 0–20 min: A/B 10:90–45:55, 20–30 min: 45:55–95:5, 30–38 min: 95:5 (isocratic). The radiochemical purity was >99% in both cases (*t*_R_ (**13**/[^3^H]**13**) = 18.0 min; *t*_R_ (**18**/[^3^H]**18**) = 18.5 min). To quantify the activity of the two radioligands and to determine the molar concentration, 2 × 2 μL of the preliminary stock solution were added to 998 μL of DMSO/H_2_O 3:7 v/v, and 4 × 10 μL of these dilutions were counted in 3 mL of liquid scintillator (Rotiscint Eco Plus, Carl Roth, Karlsruhe, Germany) with a Tri-Carb 3100TR liquid scintillation counter (PerkinElmer). The activity concentration was adjusted to 18.5 MBq/mL by the addition of ethanol/H_2_O 6:4 v/v ([^3^H]**13**: 296 μL; [^3^H]**18**: 604 μL), resulting in a final concentration of 4.76 μM for both stock solutions. Radiochemical yields [^3^H]**13**, 19.2 MBq (0.519 mCi), 35%; [^3^H]**18**, 24.9 MBq (0.674 mCi), 45%. Molar activity: As the supplier of the labeling reagent succinimidyl [^3^H]propionate (Novandi, Södertälje, Sweden) provides a precisely determined molar activity and due the fact that [^3^H]**13** and [^3^H]**18** each bear exactly one tritiated propionyl residue originating from the labeling reagent, the molar activities of [^3^H]**13** and [^3^H]**18** were defined to be equal to the molar activity of the labeling reagent, amounting to 3.89 TBq/mmol (it is assumed that under the mild reaction conditions the carbon-tritium bonds remained intact). The molar activity of the labeling reagent succinimidyl [^3^H]propionate was determined by liquid chromatography–mass spectrometry (LC-MS) analysis. Coinjection of “cold” succinimidyl propionate allowed the quantification of the incorporated tritium (Novandi, Södertälje, Sweden).

#### Chemical Stability

The chemical stability of peptides **13** and **18** was investigated in PBS (adjusted to pH 7.4) at 22 °C in the dark. The incubation was started by the addition of 15 μL of a 1 mM stock solution (solvent: DMSO) to 135 μL of PBS to yield a concentration of 100 μM. After periods of 0, 6, 24, and 48 h, an aliquot (25 μL) was removed and added to 25 μL of acetonitrile/0.04% aqueous TFA 1:9 v/v to obtain a peptide solution with a concentration of 50 μM. 20 μL of this solution were subjected to analytical RP-HPLC analysis using the same system and conditions as described under *Analytical HPLC* with the following gradient: 0–14 min: acetonitrile/0.04% aqueous TFA 10:90–30:70, 14–15 min: 30:70–95:5, 15–20 min: 95:5 (isocratic). A 1:1 mixture of PBS and acetonitrile/0.04% aqueous TFA 1:9 v/v (20 μL) was analyzed to obtain the blank chromatogram.

#### Stability in Human Plasma

The proteolytic stabilities of **13** and **18** were investigated in human blood plasma/PBS (136.9 mM NaCl, 2.68 mM KCl, 5.62 mM Na_2_HPO_4_, 1.09 mM NaH_2_PO_4_, and 1.47 mM KH_2_PO_4_, pH 7.4) 1:2 v/v according to a described procedure^38^ with the following modifications: 5 mM stock solutions in EtOH/10 mM aqueous HCl 1:1 v/v were used for the addition of the peptides to plasma/PBS 1:2 v/v. As the RP-HPLC purity of 1-methyl-d-Trp, used as internal standard (IS), was <95%, the compound was purified by preparative HPLC to give a purity of >99%. The concentration of the peptides in plasma/PBS 1:2 v/v was 40 μM (recovery determination) or 80 μM (stability tests). Samples were analyzed using the same RP-HPLC system and conditions as described under *Analytical HPLC* with the following gradient: 0–6 min: acetonitrile/0.04% aqueous TFA 10:90–21:79, 6–12 min: 21:79–40:60, 12–13 min: 40:60–95:5, 13–16 min: 95:5 (isocratic). Data analysis was based on UV detection at 220 nm. Reference samples, representing 100% recovery, were prepared in quadruplicate. Recovery ratios were obtained by dividing the recovery of the peptide by the recovery of IS for each individual sample (*n* = 4). The obtained recoveries and the recovery ratios are summarized in Table S2, Supporting Information.

#### Excitation Spectra, Emission Spectra, and Fluorescence Quantum Yields

Excitation and emission spectra of **13** and **18** were recorded with a Cary Eclipse spectrofluorimeter (Varian, Mulgrave, Victoria, Australia) using polystyrene cuvettes (10 mm × 10 mm, reference 1961, Kartell, Noviglio, Italy). Sample solutions (solvent: PBS (pH 7.4) and PBS supplemented with 1% BSA, concentration of **13** and **18**: 1 μM, sample volume: 2 mL) were prepared in the cuvettes. Excitation spectra (shown in Figure S6, Supporting Information) were recorded with spectral bandwidths of 5 nm (excitation slit) and 10 nm (emission slit). The spectral bandwidth applied for the emission spectra was 10 nm (excitation slit) and 5 nm (emission slit). Net spectra were obtained by subtracting the respective reference spectrum of a vehicle sample.

The fluorescence quantum yields of compounds **13** and **18** were determined in PBS and PBS supplemented with 1% BSA via an absolute method using an Ulbricht sphere with an inaccuracy of ca. 3% (Hamamatsu C9920-02 system equipped with a Spectralon integrating sphere) at room temperature (23 ± 1 °C). The optical density at the excitation wavelength of the sample was <0.1 (optical path length: 1 cm). Samples were measured in a 10 mm × 10 mm quartz cuvette.

#### Cell Culture

Mammalian cells were cultured in T75 or T175 culture flasks (Sarstedt). Chinese hamster ovary (CHO) cells stably expressing hNTS_1_R^49^ were cultured in DMEM/HAM’s F12 (Sigma, Taufkirchen, Germany) medium (1:1) supplemented with 7.5% FBS, l-glutamine (Sigma) (630 μg/mL) and hygromycin B (Carl Roth, Karlsruhe, Germany) (250 μg/mL). HT-29 colon carcinoma cells (DSMZ-no. ACC 299) were grown in antibiotic-free RPMI-1640 medium (Sigma) supplemented with 7.5% FBS. Both cell lines were cultured in a humidified atmosphere (95% air and 5% CO_2_) at 37 °C. Insect *Spodoptera frugiperda* (*Sf9*) cells (Invitrogen Life Technologies, Schwerte, Germany) were maintained as a suspension culture in serum-free insect cell growth medium EX-CELL 420 (Sigma-Aldrich) at 27 °C in a nonhumidified environment. Cell viability was assessed by the exclusion of 0.2% trypan blue (Sigma-Aldrich), and the cell density was determined with a TC10 Automated Cell Counter (Bio-Rad Laboratories, Sundyberg, Sweden).

#### Molecular Cloning and Baculovirus Generation

All enzymes and reagents used for cloning were obtained from Thermo Fisher Scientific (Vilnius, Lithuania) unless stated otherwise. Plasmids used to prepare baculoviruses, were generated using standard restriction cloning techniques. The hNTS_1_R-Nluc sequence from pcDNA3.1-hNTS1-NlucC (note: NlucC, fused to the C-terminus of hNTS_1_R, stands for the C-terminal fragment of Nluc consisting of 11 amino acids; the original plasmid pcDNA3.1-hNTS1 was obtained from Missouri cDNA Research Center, Rolla, MO) was digested with *Hind*III and *Mss*I to insert into the pIMACE-CMVintP10 vector^39^ that was digested with *Hind*III and *Sca*I. Restriction digests were gel purified using the FavorPrep Gel/PCR Purification Kit (Favorgen, Vienna, Austria), ligated by T4 DNA ligase (Thermo Fisher Scientific) at 22 °C for 1 h to obtain the plasmid pIMACE-CMVintP10-hNTS_1_R-Nluc, and transformed into DH5α *Escherichia coli* (made competent in house). After selection with gentamycin (MP Biomedicals, Eschwege, Germany), plasmids were extracted from overnight bacterial cultures (FavorPrep Plasmid DNA Extraction Kit). To create NTS_1_R baculoviruses pIMACE-CMVintP10-NTS_1_R-Nluc was directly used for transposition into DH10MultiBac (Geneva Biotech, Pregny-Chambésy, Switzerland) according to described standard procedures.^58^

#### CHO-hNTS_1_R Cell Membrane Preparations

CHO-hNTS_1_R cells were grown on 175 cm^2^ cell culture dishes (ref 83.3903, Sarstedt) to ca. 90% confluency. After washing two times with cold PBS (4 °C), the cells were scraped off in Tris buffer (50 mM Tris HCl, 1 mM EDTA, pH 7.4) and centrifuged at 200*g* at rt for 5 min. The cell pellet was resuspended in the aforementioned Tris buffer supplemented with SIGMAFAST protein inhibitor cocktail (Sigma) (1 tablet in 100 mL buffer). Under cooling in ice–water, cells were disrupted with an Ultraturrax (IKA, Staufen, Germany) applying six 5-s treatments with breaks of 30 s in between. The resulting homogenate was centrifuged at 1500*g* at 4 °C for 15 min. The supernatant was then centrifuged with an Optima XPN-80 ultracentrifuge (Beckmann Coulter, Brea, CA) at 119,000*g* at 4 °C for 30 min. The pellet was resuspended in Tris buffer (50 mM Tris HCl, 1 mM EDTA, pH 7.4) followed by aliquoting and storing at −80 °C. The protein concentration was determined according to the Bradford method^59^ in transparent 96-well plates (Greiner Cellstar 655180, Greiner Bio-One, Frickenhausen, Germany) using bovine serum albumin as standard. The protein concentration amounted to 1.3 ± 0.1 mg/mL (mean value ± SEM from three different sample dilutions).

### Buffers Used for Binding and Functional Assays

#### *DPBS1* (High Content Imaging, Flow Cytometry, Radiochemical Assays)

Dulbecco’s phosphate-buffered saline with calcium and magnesium (1.8 mM CaCl_2_, 2.68 mM KCl, 1.47 mM KH_2_PO_4_, 3.98 mM MgSO_4_, 137 mM NaCl, 8.06 mM Na_2_HPO_4_, pH 7.4) supplemented with 0.1% BSA (HCI, FC) or 1% BSA (radiochemical assays) and 0.1 mg/mL bacitracin. For high-content imaging and flow cytometric binding studies, *DPBS* was filtrated using 0.2 μm NY syringe filters (Phenomenex).

#### *DPBS2* (Fluorescence Anisotropy)

Dulbecco’s phosphate-buffered saline with calcium and magnesium supplemented with 0.1% Pluronic F-127 and cOmplete EDTA-free Protease Inhibitor Cocktail (Roche).

#### NTS_2_R Binding Buffer

Tris buffer (50 mM Tris HCl, 1 mM EDTA, pH 7.4) supplemented with 0.1% BSA.

#### Fura-2 Assay Buffer

HEPES buffer (120 mM NaCl, 5 mM KCl, 2 mM MgCl_2_, 1.5 mM CaCl_2_, 25 mM HEPES and 10 mM glucose at pH 7.4) supplemented with 2% BSA and 2.5 mM Probenecid (Sigma).

### Radiochemical Competition Binding Assay with [^3^H]UR-MK300

NTS_1_R radioligand competition binding experiments with [^3^H]UR-MK300 (molar activity: 2.41 TBq/mmol) and the peptides **6**–**9**, **10a**, **10b**, **13**–**16**, and **18** were performed with intact hNTS_1_R-expressing human HT-29 colon carcinoma cells at 23 ± 1 °C using a previously reported protocol.^13^ The *K*_d_ value of [^3^H]UR-MK300 amounted to 0.41 ± 0.08 nM (mean value ± SEM from two independent saturation binding experiments, each performed in triplicate). Cells were seeded one day before the assay, yielding a confluency of at least 90% on the day of the experiment. Total binding data (including total binding in the absence of competitor) were plotted as dpm values against log(concentration competitor) and analyzed by a four-parameter logistic equation (log(inhibitor) vs response–variable slope, GraphPad Prism 5, GraphPad Software, San Diego, CA) to obtain pIC_50_ values. Individual pIC_50_ values were converted to p*K*_i_ values according to the Cheng–Prusoff equation^60^ (logarithmic form). To plot average data from individual binding experiments, data were normalized (100% = “top” of the four-parameter logistic fit, 0% = unspecifically bound radioligand).

NTS_2_R radioligand competition binding experiments were performed at homogenates of HEK293T cells transiently transfected with the hNTS_2_R according to a reported procedure,^49^ but using [^3^H]UR-MK300 as radioligand instead of [^3^H]NT(8–13). Samples contained 6–10 μg of total soluble protein. Unspecific binding was determined in the presence of NT(8–13) (10 μM). Experiments were performed in triplicate and data were processed as reported.^49^ The *K*_d_ value of [^3^H]UR-MK300 amounted to 0.75 ± 0.05 nM at a receptor density of *B*_max_ = 1000 ± 60 fmol/mg protein (mean value ± SEM from four independent saturation binding experiments, each performed in triplicate).

### Fura-2 Ca^2+^ Assay

CHO-hNTS_1_R cells were seeded in T175 culture flasks 3–4 days prior to the experiment. On the day of the experiment, cells of a confluent T175 flask were detached by trypsinization and centrifuged at rt at 200*g* for 5 min. After discarding the medium, cells were resuspended in 10 mL of assay buffer followed by adjustment of the cell concentration to 1.3 × 10^6^ cells per mL. Per 1 × 10^6^ cells (corresponds to 0.77 mL of the cell suspension), 0.25 mL of loading suspension (assay buffer (1 mL), Pluronic F-127 (20% in DMSO, 5 μL), and Fura-2 AM (1 mM in DMSO, 4 μL)) were added and the cell suspension was gently stirred at rt in the dark for 30 min. After centrifugation at rt at 200*g* for 5 min, the supernatant was discarded, the cells were resuspended in assay buffer (10 mL), and the suspension was gently stirred at rt in the dark for further 30 min. This process was repeated once. Afterward, the cell density was adjusted to 1 × 10^6^ cells per mL by the addition of assay buffer to obtain the cellsuspension to be used for the assay. For the Fura-2 assay (performed in agonist mode), a 10-fold concentrated (relative to the final concentration) solution of the compound of interest (20 μL) in assay buffer was added to the empty wells of a white 96-well plate (Brand 781605, Wertheim, Germany). To determine the maximal response, NT(8–13) was used instead (final concentration: 10 nM). To obtain blank controls, neat assay buffer (20 μL) was filled into the wells. After prefilling the plate, it was subjected to a preheated (37 °C) CLARIOstar Plus Microplatereader (BMG Labtech, Ortenberg, Germany) equipped with two reagent injectors. In a single well, fluorescence (excitation = 335/380 nm, emission = 510 nm, gain = 1300/1100) was recorded for two read cycles (1.5 s). Using one of the injectors, 180 μL of the cell suspension (gently stirred using a magnetic stirrer) were injected (flow rate: 100 μL/s) into the well immediately followed by continued measurement of the fluorescence for 44 cycles (66 s). This process was conducted for all sample wells. Fluorescence signals were converted into ratios (signal_excitation 335 nm_ divided by signal_excitation 380 nm_) and the highest ratio of each well (ratio_max_) was determined. Triplicate mean values of ratio_max_ were normalized based on the response (ratio_max_) obtained for 10 nM NT(8–13) (100% = response elicited by 10 nM NT(8–13), 0% = response in the absence of agonist (blank control)) and the normalized data were plotted in % against log(concentration of agonist, M) followed by analysis according to a four-parameter logistic fit (log(agonist) vs response—variable slope, GraphPad Prism 5) to obtain pEC_50_ and *E*_max_ values (the latter correspond to the upper curve plateau). All experiments were performed in triplicate.

### High-Content Imaging Binding Experiments

CHO-hNTS_1_R or HT-29 cells were seeded one day prior to the experiment into the inner 60 wells of a black clear-bottom 96-well plate (Greiner 655090, Greiner Bio-One) at a concentration of 25,000 cells/well and 35,000 cells/well, respectively. On the day of the assay, the medium was removed by suction and the cells were washed with *DPBS1* (50 μL). After this step, *DPBS1* (180 or 200 μL) containing the nuclear dye Hoechst H33342 (2 μg/mL) was added. This solution also contained the fluorescent ligand (kinetic studies, competition binding experiments), or the fluorescent ligand was added separately (saturation binding experiments). In the case of competition binding experiments, the nonlabeled receptor ligands were also added separately (details given below). All samples were incubated at 23 ± 1 °C in the dark under gentle shaking (incubation times are specified below). The final volume per well was 200 μL. Shortly before subjecting the plate to the plate reader for image acquisition, the liquid was removed by suction and *DPBS1* (100 μL) was added. Images were acquired using an IX Micro plate reader (Molecular Devices, Sunnyvale, CA) using a 20× Plan Fluor ELWD objective, a xenon lamp light source and DAPI and TRITC excitation/emission filter cubes for imaging nuclear and fluorescent ligand staining, respectively. 2 × 2 pixel binning from the CCD camera acquisition was used to generate the images for analysis (0.645 mm/image pixel: 449 × 335 mm image size). Two sites/well were measured throughout. Images were analyzed by granularity analysis (2–3 μm diameter granules; MetaXpress 5.3, Molecular Devices, San Diego, CA) and the integrated intensity (pixel intensity × granule area) per cell was used as the raw measurement of fluorescent ligand binding. Saturation binding experiments were performed with HT-29 and CHO-hNTS_1_R cells, kinetic and competition experiments only with CHO-hNTS_1_R cells. All experiments were performed in triplicate. For all kind of experiments, unspecific binding was determined in the presence of 1 μM NT(8–13).

#### Saturation Binding Experiments

The wells were prefilled with *DPBS1* (180 μL) containing DAPI (determination of total binding) or DAPI and NT(8–13) (determination of unspecific binding), followed by the addition of 20 μL of a 10-fold concentrated (relative to the final concentration) solution of **13** or **18** in *DPBS1*. The samples were incubated for 2 h, followed by the washing step and image acquisition. A concentration range of 0.07–100 nM (**13**) or 0.15–150 nM (**18**) was used. Specific binding data, obtained by subtracting triplicate mean values of unspecific binding from triplicate mean values of total binding, were plotted against the fluorescent ligand concentration and analyzed by a two-parameter equation describing hyperbolic binding (one site, specific binding, GraphPad Prism 5) to obtain *K*_d_ values.

#### Association Experiments

After the initial washing step, 200 μL of *DPBS1* containing the fluorescent ligand **13** or **18** (2.5 nM) and DAPI were added to determine total binding. To determine unspecific binding, the same solution, but additionally containing NT(8–13), was added. Samples were incubated for different periods of time. The samples of the different time points were prepared in reversed order (longest incubation time first, shortest incubation time last) so that the cells of all samples could be washed and measured approximately at the same time (note: the measurement time for 60 wells was <5 min). The studied time span was 1–180 min for both fluorescent ligands. Specific binding data, obtained by subtracting triplicate mean values of unspecific binding from triplicate mean values of total binding, were plotted against the time and analyzed by a three-parameter equation describing an exponential rise to a maximum (one-phase association, *Y*_0_ constrained to zero, GraphPad Prism 5) to yield the observed association rate constant *k*_obs_. To calculate mean values in %, specific binding data were normalized based on the corresponding *B*_eq_ value.

#### Dissociation Experiments

After initial washing of the cells, 200 μL of *DPBS1* containing the fluorescent ligand **13** or **18** (2.5 nM) and DAPI were added to the wells (determination of total binding). To determine unspecific binding, the same solution, but additionally containing NT(8–13), was added. All samples were preincubated for 90 min, followed by removal of the liquid by suction and addition of 200 μL of *DPBS1* containing NT(8–13) (1 μM) to initiate the dissociation process. The samples of the different time points were prepared at different times in reversed order (longest incubation time first, shortest incubation time last) so that the cells of all samples could be measured approximately at the same time. The studied dissociation times were 1–240 min. The plate was directly subjected to the plate reader (no washing step). Specific binding data, obtained by subtracting triplicate mean values of unspecific binding from triplicate mean values of total binding, were plotted against the time and analyzed by a three-parameter equation describing an incomplete monophasic exponential decline (one phase decay, GraphPad Prism 5) to obtain *k*_off_ values. The mean ± SEM of the plateau values from individual experiments proved to be throughout significantly different from zero (one-tailed *t* test, *P* < 0.05). To calculate mean values in %, binding data were normalized based on specifically bound ligand (*B*) at *t* = 0 (*B*_0_).

#### Calculation of Association Rate Constants (*k*_on_) and Kinetically Derived Dissociation Constants *K*_d_(kin)

The association rate constants were calculated from *k*_obs_ mean values, *k*_off_ mean values, and the fluorescent ligand concentration used for the association experiments ([FL]) according to the equation *k*_on_ = (*k*_obs_ – *k*_off_)/[FL]. The kinetically derived dissociation constants *K*_d_(kin) were calculated from *k*_on_ and *k*_off_ mean values according to *K*_d_(kin) = *k*_off_/*k*_on_.

#### Competition Binding Experiments

After initial washing of the cells, 180 μL of *DPBS1* containing the fluorescent ligand (used final concentrations corresponded to the *K*_d_ values obtained from equilibrium saturation binding experiments: 1.1 nM (**13**) or 1.3 nM (**18**)) and DAPI (determination of total binding) or fluorescent ligand (**13**: 1.1 nM, **18**: 1.3 nM), DAPI and NT(8–13) (determination of unspecific binding) were added, followed by the addition of 20 μL of a 10-fold concentrated (relative to the final concentration) solution of the compound of interest (NT(8–13) or SR142948). Samples were incubated for 90 min, followed by the washing step and image acquisition. Total binding fluorescence intensities (including total binding in the absence of competitor) were plotted against log(concentration competitor) and analyzed by a four-parameter logistic equation (log(inhibitor) vs response–variable slope, GraphPad Prism 5, GraphPad Software, San Diego, CA) to obtain pIC_50_ values. Individual pIC_50_ values were converted to p*K*_i_ values according to the Cheng–Prusoff equation^60^ (logarithmic form). To plot average data from individual binding experiments, data were normalized (100% = “top” of the four-parameter logistic fit, 0% = unspecifically bound fluorescent ligand).

### Flow Cytometric Binding Experiments

Flow cytometry-based NTS_1_R binding studies were performed with intact CHO-hNTS_1_R (all kind of experiments) and HT-29 cells (only saturation binding) using a BD FACSCanto II (Becton Dickinson, Heidelberg, Germany), equipped with an argon laser (488 nm) and a red diode laser (640 nm), and a BD High Throughput Sampler (HTS unit) for microtiter plates. Saturation and competition binding experiments were performed in triplicate in 96-well polypropylene plates (Brand 701330, Wertheim, Germany) using the HTS unit for sample injection. Association and dissociation experiments were performed in duplicate in 5 mL polypropylene tubes (VWR, Radnor) using the standard injection port of the flow cytometer. The following gain settings for forward and sideward scatter were applied: FSC, 0 V; SSC, 252 V. Fluorescence was recorded using the PE-channel (excitation: 488 nm, emission: 585 ± 21) nm with a PMT gain of 330–400 V. For measurements using the HTS unit, 45 μL of the sample were injected with a speed of 1.5 μL/s. For measurements, requiring the use of the standard injection port, the medium flow rate (60 mL/min) was used. Measurements were stopped after 30 s (HTS unit) or after counting of 500–3000 gated events (standard injection port).

Cells were seeded in T75 culture flasks 3–4 days (CHO-hNTS_1_R cells) or 5–6 days (HT-29 cells) prior to the experiment. On the day of the experiment, cells were detached by trypsinization, suspended in *DPBS1* and centrifuged at 200*g* at rt for 5 min. The cell pellet was resuspended in *DPBS1* and the cell density was adjusted to 1.5 × 10^5^ to 2.5 × 10^5^ cells/mL (CHO-hNTS1R cells) or 1.0 × 10^6^ cells/mL (HT-29 cells). Unspecific binding was determined in the presence of 1 μM NT(8–13).

#### Saturation Binding Experiments

A 96-well polypropylene plate was prefilled with 200 μL of cell suspension. For total binding, DMSO/H_2_O 2:8 v/v (2 μL) and DMSO/H_2_O 2:8 v/v (2 μL) containing the fluorescent ligand (100-fold concentrated compared to the final concentration), were added. To determine unspecific binding, a 100 μM solution of NT(8–13) in water (2 μL) and DMSO/H_2_O 2:8 v/v (2 μL) containing the fluorescent ligand (100-fold concentrated), were added. Samples were incubated at 23 ± 1 °C in the dark under gentle shaking for 2 h followed by measurement via the HTS unit. Specific binding data, obtained by subtracting triplicate mean values of unspecific binding from triplicate mean values of total binding, were plotted against the fluorescent ligand concentration and analyzed by a two-parameter equation describing hyperbolic single-site binding (one site, specific binding, GraphPad Prism 5) to obtain *K*_d_ values.

#### Association Experiments

5 mL polypropylene tubes were prefilled with 2000 μL of cell suspension and DMSO/H_2_O 2:8 v/v (20 μL) were added (determination of total binding). To start the association, a 100 nM solution of the fluorescent ligand in DMSO/H_2_O 2:8 v/v (20 μL) was added (final concentration of **13** and **18**: 1 nM). After short mixing, the tube was placed in the injection port of the flow cytometer and the data of the first (20 s), second (40 s) and third (60 s) time point were acquired. Additional measurements were conducted within different periods of time (2–180 min). When not placed in the injection port, the sample tube was gently shaken in the dark at 23 ± 1 °C. To determine unspecific binding, a 100 μM solution of NT(8–13) in water (20 μL) and a 100 nM solution of the fluorescent ligand in DMSO/H_2_O 2:8 v/v (20 μL) were added. Specific binding data, obtained by subtracting triplicate mean values of unspecific binding from triplicate mean values of total binding, were plotted against the time and analyzed by a three-parameter equation describing an exponential rise to a maximum (one-phase association, *Y*_0_ constrained to zero, GraphPad Prism 5) to yield the observed association rate constant *k*_obs_. To calculate mean values in %, specific binding data were normalized based on the corresponding *B*_eq_ value.

#### Dissociation Experiments

5 mL polypropylene tubes were prefilled with 2000 μL of cell suspension. For the determination of total binding, the preincubation was started by the addition of DMSO/H_2_O 2:8 v/v (20 μL) and a 1 μM solution of **13** or **18** in DMSO/H_2_O 2:8 v/v (20 μL) (final fluorescent ligand concentration: 10 nM). To determine unspecific binding, a 100 μM solution of NT(8–13) in water (20 μL) and a 1 μM solution of **13** or **18** in DMSO/H_2_O 2:8 v/v were added. The samples were gently shaken in the dark at 23 ± 1 °C for 90 min. The dissociation process was initiated by the addition of a 2.5 mM solution of NT(8–13) in water (20 μL) (final concentration: approximately 25 μM). After different periods of time (1–240 min), sample aliquots were measured by placing the tube in the injection port of the flow cytometer. Specific binding data, obtained by subtracting triplicate mean values of unspecific binding from triplicate mean values of total binding, were plotted against the time and analyzed by a three-parameter equation describing an incomplete monophasic exponential decline (one phase decay, GraphPad Prism 5) to obtain *k*_off_ values. The mean ± SEM of the plateau values from individual experiments proved to be throughout significantly different from zero (one-tailed *t* test, *P* < 0.05). To calculate mean values in %, binding data were normalized based on the specifically bound ligand (*B*) at *t* = 0 (*B*_0_).

#### Calculation of Association Rate Constants (*k*_on_) and Kinetically Derived Dissociation Constants *K*_d_(kin)

The *k*_on_ and *K*_d_(kin) values were calculated as described for high-content imaging binding experiments.

#### Competition Binding Experiments

A 96-well polypropylene plate was prefilled with 200 μL of cell suspension. Two μL of DMSO/H_2_O 2:8 v/v (for the determination of total binding in the absence of competitor), 2 μL of a 100 μM solution of NT(8–13) in water (determination of unspecific binding) or 2 μL of a 100-fold concentrated solution (compared to the final concentration) of the compound of interest (NT(8–13) or SR142948; used at varying concentrations) in DMSO/H_2_O 2:8 v/v, were added and the plate was shortly shaken. Subsequently, 2 μL of a 260 nM solution of **13** or a 310 nM solution of **18** in DMSO/H_2_O 2:8 v/v were added to each well and the plate was gently shaken in the dark at 23 ± 1 °C for 90 min followed by measurement via the HTS unit. The final concentrations of **13** and **18** corresponded to their *K*_d_ values determined by equilibrium saturation binding: 2.6 nM (**13**) or 3.1 nM (**18**). Data were analyzed as described for HCI competition binding experiments.

### Fluorescence Anisotropy Binding Experiments

FA-based NTS_1_R binding studies with **13** and **18** were performed at hNTS_1_R displaying budded baculovirus particles (termed BBVs below), which were obtained from Sf9 insect cells following the procedure reported for the preparation of neuropeptide Y Y_1_R displaying BBVs.^61^ The obtained viruses were collected by centrifugation at 1600*g* for 10 min, and the virus titer was determined with an image-based cell-size estimation assay as described elsewhere.^62^ The viruses were amplified using a multiplicity of infection (MOI) between 0.01 and 0.1 until a high-titer baculovirus stock (virus concentration of at least 1.0 × 10^8^ infectious viral particles/mL) was acquired. To produce BBV preparations with a high receptor density, Sf9 cells with a density of 1.9 × 10^6^ cells/mL were infected with an MOI of 3 and the BBVs were collected by centrifugation 63 h after the infection, when the cell viability was <30%. The BBVs were concentrated 40-fold by centrifugation at 48,000*g* at 4 °C for 40 min, washed with ice-cold DPBS (without supplements), resuspended, and homogenized using a 0.3 mm diameter needle (Sterican, B. Braun, Melsungen, Germany). Aliquots of the BBV preparations were stored at −90 °C until use.

FA measurements were performed with a Synergy NEO multimode plate reader (BioTek, Winooski, VT) and black, half area, flat bottom polystyrene nonbinding surface (NBS) 96-well plates (product no. 3993; Corning, Corning, NY). Polarizing excitation (530 ± 15 nm) and dual emission (590 ± 17.5 nm) filters with a dichroic mirror were used, allowing the simultaneous detection of parallelly and perpendicularly polarized fluorescence emission after polarizing beam splitter. All measurements were performed at 27 °C. For all experiments, *DPBS2* was used as binding buffer. Saturation binding, association and dissociation experiments were performed in duplicate (total binding) and singlet (unspecific binding). Competition binding experiments were carried out in triplicate. The final total volume per well was 100 μL. On the day of the experiment, frozen preparations of hNTS_1_R displaying BBVs were thawed and thoroughly resuspended, followed by further dilution required for the respective experiment.

#### Saturation Binding, Association, and Dissociation Experiments

25 μL of a 4-fold concentrated (compared to the final concentration) solution of **13** or **18** (final concentrations were 0.3 and 1 nM for both ligands) and 25 μL of *DPBS2* were premixed in the 96-well plate followed by the addition of 50 μL of BBV suspension (used in six different concentrations; each was combined with the two ligand concentrations) to start the association. To determine unspecific binding, 25 μL of a 4 μM solution of SR142948 in *DPBS2* was added instead of neat *DPBS2*. For blank measurements, 50 μL of *DPBS2* were mixed with 50 μL of BBV suspension. Due to the very fast association of both fluorescent ligands, separate measurements were performed for the individual combinations of fluorescent ligand and BBV concentrations (each measurement comprised 4 wells: 2 × total binding, 1 × unspecific binding, 1 × blank). This enabled shorter time intervals between the read cycles (the measuring time for one cycle, comprising 4 wells, was 6 s; 100 read cycles were carried out). The time span between the addition of the ligands to the wells using a multichannel pipet and the start of the measurement was 7 s (no shaking carried out). It was taken into account for data analysis. After 10 min of reading (within this time period a stable signal was reached for all combinations of ligand and BBV concentrations), the plate was removed and 5 μL of a 21 μM solution of SR142948 in *DPBS2* (final concentration: 1 μM) were added to start the dissociation. Immediately after the addition of SR142948, FA signals were measured for 10 min (within this time period, the dissociation was complete and a plateau was reached, respectively). Equilibrium binding was analyzed using the FA signals at 10 min. Fluorescence intensities were processed as previously reported^61^ using the in-house developed software Aparecium 2.0.20 (http://www.gpcr.ut.ee/software.html).^63^ Prior to the calculation of fluorescence anisotropy values, the parallel and perpendicular fluorescence intensities were blank-corrected based on the data obtained from wells containing BBVs, but no fluorescent ligand. The FA signals at 10 min were used to analyze equilibrium saturation binding using a previously presented user-defined GraphPad Prism-compatible binding model (https://github.com/laasfeld/FPoLi-GPCR)^63^ to obtain *K*_d_ values. The association rate constant *k*_on_ and the *K*_d(kin)_ values were obtained by fitting the binding data with a reported model^63^ with *k*_off_ constrained to the value derived from the dissociation section of the respective experiment. Mean *K*_d(kin)_ values ± SEM were calculated from individual *K*_d(kin)_ values. The NTS_1_R concentrations of the BBV stocks (two batches were used), obtained from the global data analysis, amounted to 1.4 and 10.0 nM.

#### Competition Binding Experiments

25 μL of a 1.2 nM solution of **13** or **18** in *DPBS2* and 25 μL of a 4-fold concentrated (compared to the final concentration) solution of the compound of interest (used at varying concentrations) in *DPBS2* were premixed in a 96-well plate. To determine total binding, 50 μL of the BBV suspension (used at one fixed dilution corresponding to a receptor concentration of 1.0 nM) were added. To determine total binding in the absence of competitor, 25 μL of neat *DPBS2* were added instead of *DPBS2* containing the competing ligand. Unspecific binding was determined by the addition of 25 μL of a 4 μM solution of SR142948 in *DPBS2* (final concentration: 1 μM). For blank measurements, 50 μL of *DPBS2* were mixed with 50 μL of BBV suspension. The incubation time was 90 min. Anisotropy data of total binding (including total binding in the absence of competitor) were plotted against log(concentration competitor) and analyzed by a four-parameter logistic equation (log(inhibitor) vs response–variable slope, GraphPad Prism 5, GraphPad Software, San Diego, CA) to obtain pIC_50_ values. Individual pIC_50_ values were converted to p*K*_i_ values according to the Cheng–Prusoff equation^60^ (logarithmic form). To plot average data from individual binding experiments, data were normalized (100% = “top” of the four-parameter logistic fit, 0% = unspecifically bound fluorescent ligand). It should be noted that in the case of FA, data fitting by a four-parameter logistic equation and conversion of the resulting pIC_50_ values to p*K*_i_ values according to the Cheng–Prusoff equation represents an approximation since it assumes abundance of the ligands relative to the amount of receptor, which may be violated in the case of FA assay, especially when measuring high-affinity unlabeled ligands.

### Radiochemical NTS_1_R Binding Assays with [^3^H]**13** and [^3^H]**18**

Binding of both ligands, [^3^H]**13** and [^3^H]**18**, was studied at adherent and suspended intact CHO-hNTS_1_R cells, and binding of [^3^H]**13** was additionally studied using CHO-hNTS_1_R cell membrane preparations (saturation and competition binding, kinetic studies). Furthermore, saturation binding experiments were performed with both ligands at intact adherent HT-29 cells. All experiments were performed in triplicate except for dissociation experiments performed with suspended cells in 50 mL flacon tubes (performed in duplicate). To reduce radioligand consumption in the aforementioned experiments, [^3^H]**13** and [^3^H]**18** were 1:1 diluted with **13** and **18**, respectively (for the purpose of easier reading these mixtures are denoted "[^3^H]**13**" and "[^3^H]**18**"in the following). All samples were incubated at 23 ± 1 °C in the dark under gentle shaking (incubation times are outlined below). *DPBS1* served as binding buffer throughout. In the case of experiments performed with cell suspensions, CHO-hNTS_1_R cells were seeded in T75 culture flasks 3–4 days prior to the experiment. On the day of the experiment, cells were detached by trypsinization, suspended in *DPBS1* and centrifuged at 200*g* at rt for 5 min. Cells were resuspended in binding buffer and the cell density was adjusted to 1.5 × 10^5^ to 2.5 × 10^5^ cells/mL. Samples involving suspended cells or membrane preparations were prepared and incubated in 96-well polypropylene plates (Brand 701330, Wertheim, Germany) or in 50 mL polypropylene falcon tubes (Sarstedt), and the same filtration procedure for separating free radioligand from cell- or membrane bound radioligand was applied for all kind of binding experiments: after completed incubation, cells or cell membranes were collected on GF/C filter mats (0.26 mm; Whatman, Maidstone, U.K.) (pretreated with 0.3% polyethylenimine for 30 min) using an in-house manufactured harvester for 96-well plates (precision engineering workshop of the University of Regensburg, Regensburg, Germany), and the wells of the plate and the cells on the filter mat were immediately washed twice with ice-cold PBS (2 × 200 μL) (note: when samples were incubated in falcon tubes, 200-μL aliquots were transferred to the wells of a 96-well polypropylene plate after the incubation period, immediately followed by the filtration procedure). Filter pieces for each well were punched out and transferred into 1450–401 96-well sample plates (PerkinElmer, Rodgau, Germany), followed by the addition of Rotiscint Routine (Carl Roth, Karlsruhe, Germany) (200 μL). The plates were sealed with transparent sealing tape (Greiner EASYseal, part no. 676001, Greiner Bio-One) and shaken in the dark overnight before measurement of the radioactivity (dpm) using a MicroBeta2 plate counter equipped with six pairs of photomultiplier tubes (PerkinElmer).

For experiments involving adherent cells, CHO-hNTS_1_R (25,000 cells per well) or HT-29 (35,000 cells per well) cells were seeded one day prior to the experiment in white 96-well plates with clear bottom (VWR 732–3740). On the day of the experiment, the medium was aspirated and the cells (confluency: 70–90%) were washed with PBS (200 μL) followed by covering with *DPBS1* (160 μL) and the addition of the ligands as outlined below. After completed incubation, the liquid was aspirated and the wells were washed twice with ice-cold PBS (200 μL) followed by the addition of lysis solution (urea (8 M), acetic acid (3 M) and Triton-X (1%) in water) (25 μL). After shaking for at least 20 min, liquid scintillator (UltimaGold, PerkinElmer) (200 μL) was added and the plates were sealed with a transparent sealing tape (permanent seal for microplates, PerkinElmer, prod. no. 1450–461). The sealed plates were turned upside down several times to achieve complete mixing of scintillator and lysis solution and were kept in the dark for at least 3 h before measurement of the radioactivity (dpm) with the MicroBeta2 plate counter.

#### Saturation Binding Experiments

A 96-well polypropylene plate was prefilled with cell suspension (160 μL) or 96-well white/clear bottom plates with adherent cells were prefilled with *DPBS1* (160 μL) after initial washing of the cells. For the determination of total binding, *DPBS1* (20 μL) and a 10-fold concentrated (relative to the final concentration) solution of [^3^H]**13** or [^3^H]**18** in *DPBS1* (20 μL) were added. To determine unspecific binding, a 10 μM solution of NT(8–13) (final concentration: 1 μM) *in DPBS1* (20 μL) was added instead of neat *DPBS1*. Samples were incubated for 2 h followed by cell harvesting (cell suspensions) or cell lysis (adherent cells) and further processing as described afore. Specific binding data, obtained by subtracting triplicate dpm mean values of unspecific binding from triplicate dpm mean values of total binding, were plotted against the free radioligand concentration and analyzed by a two-parameter equation describing hyperbolic single-site binding (one site, specific binding, GraphPad Prism 5) to obtain *K*_d_ values. The free concentration of [^3^H]**13** or [^3^H]**18** (nM) was calculated by subtracting the amount of specifically bound [^3^H]**13** or [^3^H]**18** (nM) (calculated from specifically bound radioligand in dpm, the molar activity and the volume per well) from the total concentration of [^3^H]**13** or [^3^H]**18**.

#### Association Experiments

##### Cell Suspensions

After prefilling a 96-well polypropylene plate with cell suspension (160 μL), 20 μL of *DPBS1* (determination of total binding) or 20 μL of a 10 μM solution of NT(8–13) (final concentration: 1 μM) in *DPBS1* (determination of unspecific binding) were added. To start the association, 20 μL of an 18 nM solution of [^3^H]**13** or 20 μL of a 12 nM solution of [^3^H]**18** in *DPBS1* (final concentrations: 1.8 or 1.2 nM) were added. The samples of the different time points were prepared in reversed order (longest incubation time first, shortest incubation time last) so that the cells of all samples could be harvested simultaneously. The time span for sample incubation was 1–180 min. **Adherent cells:** 96-well white/clear bottom plates with adherent cells were prefilled with *DPBS1* (160 μL) after initial washing of the cells. 20 μL of *DPBS1* (determination of total binding) or 20 μL of a 10 μM solution of NT(8–13) in *DPBS1* (final concentration: 1 μM) (determination of unspecific binding) were added. To start the association, 20 μL of a 13 nM solution of [^3^H]**13** or 20 μL of an 11 nM solution of [^3^H]**18** in *DPBS1* (final concentrations: 1.3 or 1.1 nM) were added. When the incubation time was reached, all liquid was removed by suction followed by washing (2 × ice-cold PBS) and cell lysis. The samples of the different time points were prepared in reversed order (longest incubation time first, shortest incubation time last) so that the cells of all samples could be washed and lysed simultaneously. This did not apply for the first four time points (1, 3, 5, 8 min). These samples were prepared and processed individually during the incubation of the samples of the longer time points (note: lysis solution was not directly added after washing of the cells; it was added when the incubation of all samples had been finished). The time span for the association was 1–180 min. **Data processing (cell suspensions and adherent cells):** Specific binding data, obtained by subtracting triplicate dpm mean values of unspecific binding from triplicate dpm mean values of total binding, were plotted against the time. Data describing the first association phase were fitted by a one phase association fit (*Y*_0_ constrained to zero, GraphPad Prism 5) followed by a biphasic fit (two phase association, *Y*_0_ constrained to zero, *k*_obs(fast)_ constrained to the *k*_obs_ value to the initial monophasic fit, GraphPad Prism 5) affording *k*_obs(slow)_ values. To calculate mean values in %, specific binding data were normalized based on the corresponding plateau value (set to 100%) obtained for the biphasic fit.

#### Dissociation Experiments

##### Cell Suspensions

Initially, dissociation experiments with [^3^H]**13** and [^3^H]**18** using cell suspensions were performed in 96-well polypropylene plates. The plates were prefilled with cell suspension (160 μL) followed by the addition of 20 μL of *DPBS1* (determination of total binding) or 20 μL of a 10 μM solution of NT(8–13) (final concentration: 1 μM) in *DPBS1* (determination of unspecific binding) to start the preincubation period of 90 min. The dissociation process was initiated by the addition of a 250 μM solution of NT(8–13) in *DPBS1* (20 μL) (final concentration: approximately 25 μM). The samples of the different time points were prepared at different times in reversed order (longest incubation time first, shortest incubation time last) so that the cells of all samples could be harvested simultaneously. The studied dissociation times were 1–240 min. As these dissociation experiments did not yield useful (reproducible) results, dissociation experiments were performed using 50 mL falcon tubes as sample container. These were prefilled with 5000 μL of cell suspension followed by the addition of *DPBS1* (50 μL) and a 500 nM solution of [^3^H]**13** or [^3^H]**18** (final concentration: 5 nM) in *DPBS1* (50 μL). To determine unspecific binding, a 100 μM solution of NT(8–13) (final concentration: 1 μM) in *DPBS1* (50 μL) was added instead of neat *DPBS1*. The samples were preincubated for 90 min. The dissociation was initiated by the addition of 50 μL a 2.5 mM solution of NT(8–13) in *DPBS1* (final concentration: 25 μM). As this kind of dissociation experiment is laborious with respect to the separation of bound from free radioligand, only a few time points (10, 30, 60, 90, 120, 150, and 180 min) were studied. After the indicated incubation periods, three 200-μL aliquots were removed and transferred into a 96-well polypropylene plate immediately followed by cell harvesting and further processing as described afore (filter pieces were collected in one 1450–401 96-well sample plate). Note that the experimental setup necessitated an individual filtration process for each incubation time. **Adherent cells:** The inner 60 wells of two white 96-well plates with clear bottom were prefilled with *DPBS1* (160 μL) after initial washing of the cells. The preincubation was started by the addition of *DPBS1* (20 μL) and a 65 nM solution of [^3^H]**13** or a 55 nM solution of [^3^H]**18** (final concentrations 6.5 nM and 5.5 nM, respectively) in *DPBS1* (20 μL). To determine unspecific binding, a 10 μM solution of NT(8–13) (final concentration: 1 μM) in *DPBS1* (20 μL) was added instead of neat *DPBS1*. The samples were incubated for 90 min followed by removal of all liquid by suction and the addition of a 1 μM solution of NT(8–13) in *DPBS1* (200 μL) to initiate the dissociation. The samples of the different time points were prepared at different times in reversed order (longest incubation time first, shortest incubation time last) so that the cells of all samples could be washed and lysed at approximately the same time. This did not apply for the four shortest time points (1, 3, 5, and 8 min). These samples were prepared and processed individually during the incubation of the samples of the longer time points (note: lysis solution was not directly added after washing of the cells; it was added when the incubation of all samples had been finished). The time span for the dissociation was 1–180 min.

Specific binding data, obtained by subtracting triplicate mean values of unspecific binding from triplicate mean values of total binding, were plotted against the time and analyzed by a three-parameter equation, describing an incomplete monophasic exponential decline (one phase decay, GraphPad Prism 5) to obtain *k*_off_ values. The mean ± SEM of the plateau values from individual experiments proved to be throughout significantly different from zero (one-tailed *t* test, *P* < 0.05). To calculate mean values in %, binding data were normalized based on the specifically bound ligand (*B*) at t = 0 (*B*_0_).

#### Calculation of Association Rate Constants (*k*_on_) and Kinetically Derived Dissociation Constants *K*_d_(kin)

The association rate constants *k*_on(fast)_ were calculated from *k*_obs(fast)_ mean values, *k*_off_ mean values, and the radioligand concentration used for the association experiments ([RL]) according to the equation *k*_on(fast)_ = (*k*_obs(fast)_ – *k*_off_)/[RL]. To note, the calculation of *k*_on(slow)_ from *k*_obs(slow)_ gave negative values since the *k*_off_ mean value was higher than the individual *k*_obs(slow)_ values. The kinetically derived dissociation constants *K*_d_(kin) were calculated from *k*_on(fast)_ and *k*_off_ mean values according to *K*_d_(kin) = *k*_off_/*k*_on(fast)_.

#### Competition Binding Experiments

A 10-fold concentrated solution (compared to the final concentration) of [^3^H]**13** or [^3^H]**18** in *DPBS1* (20 μL) was added to 160 μL of cell suspension (final radioligand concentrations: 1.8 and 1.2 nM, respectively) or to the adherent cells covered with 160 μL of *DPBS1* (final radioligand concentrations: 1.3 and 1.1 nM, respectively), followed by the addition of a 10-fold concentrated solution (compared to the final concentration) of the compound of interest in *DPBS1* (20 μL). Total binding in the absence of competitor was determined by adding *DPBS1* (20 μL) instead of competitor, and unspecific binding was determined by the addition of a 10 μM solution of NT(8–13) (final concentration: 1 μM) in *DPBS1* (20 μL) instead of competitor. Samples were incubated for 90 min followed by cell harvesting or cell lysis and further processing as described afore. Data were analyzed as described for HCI competition binding experiments.

#### Saturation Binding, Association, and Dissociation Experiments with [^3^H]**13** at Membranes of CHO-hNTS_1_R Cells

On the day of the experiment, membrane aliquots (150 μL) were thawn and *DPBS1* (300 μL) was added. The suspension was centrifuged at 9000*g* at 4 °C for 5 min followed by discarding the supernatant and resuspension in *DPBS1* supplemented with saponin (100 μg/mL) and GppNHp (50 μM) (approximately 2 mL). The membrane dilution was homogenized using a 0.4 mm diameter needle (Sterican, B. Braun, Melsungen, Germany). Experiments were performed as the saturation binding, association, and dissociation experiments with suspended CHO-hNTS_1_R cells, using 20 μL of the membrane dilution in *DPBS1* (soluble protein content: 98 μg/mL) instead of 160 μL of the cell suspension. To reach a volume of 160 μL, 140 μL of *DPBS1* with saponin and GppNHp were added to each well prior to addition of the ligands. Data were processed and analyzed as described for HCI binding assays. As the plateau mean value from individual dissociation experiments (one phase decay fit, GraphPad Prism 5) was not significantly different from zero (*P* > 0.05, unpaired one-tailed *t* test), data were reanalyzed with the plateau value constrained to zero to obtain *k*_off_ values.

#### Confocal Microscopy

Confocal microscopy was performed with a Celldiscoverer 7 (Zeiss). The used objective was 50× magnification with water (1.2 NA) (Zeiss). Experiments were performed at 23 ± 1 °C. One day prior to the experiment, CHO-hNTS_1_R cells were seeded into a black clear-bottom 96-well plate (Greiner 655090) at a concentration of 25,000 cells/well. Shortly before image acquisition, the medium of one well was removed by suction and the cells were washed with *DPBS1* (50 μL), followed by the addition of *DPBS1* (200 μL) containing Hoechst H33342 (2 μg/mL) and **13** or **18** (each 5 nM). The plate was immediately transferred to the instrument and a series of 30 images (1–30 min) was acquired using the following settings: sequential laser excitation (405 nm nuclei, 561 nm **13**/**18**), emission (410–540 nm band-pass, nuclei, 565–700 nm long pass **13**/**18**), with a pinhole set to 1 Airy unit for the longer wavelength. For the determination of unspecific binding, NT(8–13) (1 μM) was added to the solution containing H33342 and **13** or **18**.

#### Calculation of Propagated Errors

Propagated errors (applying to specifically bound fluorescent ligand or radioligand (saturation binding)), association rate constants *k*_on_ (except for FA assays), and kinetically derived dissociation constants *K*_d_(kin) (except for FA assays) were calculated as described elsewhere.^56^

## Supporting Information Available

Structures and NTS_1_R binding affinities of reported NT(8–13) derivatives containing β,β-dimethyl-l-tyrosine or β,β-dimethyl-d-tyrosine in position 11 (Figure S1); investigation of the chemical stability of **13** and **18**: chromatograms of the HPLC analyses (Figures S2 and S3); radioligand displacement curves from competition binding experiments with [^3^H]UR-MK300 (Figure S4); concentration response curves of NT(8–13), **6**, **10a**, **13** and **18** obtained from a Fura-2 Ca^2+^ assay (Figure S5); excitation and corrected emission spectra of **13** and **18** (Figure S6); NTS_1_R equilibrium binding of **18** and [^3^H]**18** studied by different methods (Figure S7); binding kinetics of **18** and [^3^H]**18** studied by different methods (Figure S8); displacement curves and corresponding p*K*_i_ values from competition binding studies performed with **18** or [^3^H]**18** and NT(8–13) and SR142948 (Figure S9); fluorescence images acquired with a Celldiscoverer 7 showing binding of **13** to CHO-hNTS_1_R cells (Figures S10–S12); fluorescence images acquired with a Celldiscoverer 7 showing binding of **18** to CHO-hNTS_1_R cells (Figures S13–S15); representative fluorescence images acquired with an IX Micro Confocal plate reader of a high-content imaging dissociation experiment performed with **13** at intact adherent CHO-hNTS_1_R cells (Figure S16); data of dissociation experiments performed with [^3^H]**13**, [^3^H]**18** and [^3^H]UR-MK300 in 96-well PP plates at suspended CHO-hNTS_1_R cells (Figure S17); synthesis of the azido-functionalized fluorescent dye **17** (Scheme S1, Supporting Information); stabilities of **13** and **18** in human plasma/PBS 1:2 v/v (Table S1); recoveries of **13** and **18** from human plasma/PBS 1:2 v/v and ratios of compound-recovery over recovery of internal standard (Table S2); RP-HPLC chromatograms of **6**–**9**, **10a**, **10b** and **13**–**20** (purity controls); ^1^H NMR spectra of **6**–**9**, **10a**, **13** and **18**, and ^13^C NMR spectra of **6**–**9** and **10a** in DMSO-*d*_6_ and DMSO-*d*_6_/D_2_O 4:1 (PDF)
Molecular formula strings and NTS_1_R receptor affinities (CSV)

## Abbreviations Used

Arg(carb): *N*^ω^-carbamoylated arginine
BBV: budded baculovirus
BRET: bioluminescence resonance energy transfer
CH_2_Cl_2_: dichloromethane
CHO cells: Chinese hamster ovary cells
2-ClTrt: 2-chlorotrityl
DPBS: Dulbecco′s phosphate buffered saline
DIPEA: diisopropylethylamine
FA: fluorescence anisotropy
FBS: fetal bovine serum
FC: flow cytometry
GAB: γ-aminobutyryl
GppNHp: Guanosine-5′-[(β,γ)-imido]triphosphate
HBTU: 3-[bis(dimethylamino)methyliumyl]-3*H*-benzotriazol-1-oxide hexafluorophosphate
HCI: high content imaging
HEPES: 2-[4-(2-hydroxyethyl)piperazin-1-yl]ethane-1-sulfonic acid
HOBt: hydroxybenzotriazole
*K*_d_: dissociation constant from a saturation binding experiment
*k*_obs_: observed association rate constant
*k*_off_: dissociation rate constant
*k*_on_: association rate constant
NTS_1_R: neurotensin receptor type 1
NTS_2_R: neurotensin receptor type 2
pEC_50_: negative decadic logarithm of the half maximal effective concentration (functional assays)
pipGly: piperidinylglycine
p*K*_i_: negative decadic logarithm of the dissociation constant *K*_i_ (in M) obtained from a competition binding experiment
Pra: propargylglycine
SEM: standard error of the mean
SPPS: solid phase peptide synthesis
