# Supplementary material for: Dually Labeled Neurotensin NTS1R Ligands for Probing Radiochemical and Fluorescence-Based Binding Assays
Source: J Med Chem. 2024 Sep 11;67(18):16664–91. doi: 10.1021/acs.jmedchem.4c01470 (PMC11440508; doi:10.1021/acs.jmedchem.4c01470)
Supplement: Supplementary file 1 — jm4c01470_si_001.pdf [file jm4c01470_si_001.pdf]

Supporting Information

Dually labeled neurotensin NTS_1R ligands for probing radiochemical and fluorescence-based binding assays

Fabian J. Ertl,[†] *Sergei Kopanchuk,*[‡] *Nicola C. Dijon,*[#] *Santa Veikšina,*[‡] *Maris-Johanna Tahk,*[‡] *Tõnis Laasfeld,*[‡] *Franziska Schettler,*[†] *Albert O. Gattor,*[†] *Harald Hübner,*[§] *Nataliya Archipowa,*[||] *Johannes Köckenberger,*[§] *Markus R. Heinrich,*[§] *Peter Gmeiner,*[§] *Roger J. Kutta,*[⊥] *Nicholas D. Holliday,*[#,*] *Ago Rinken,*[‡,*] *and Max Keller*[†,*]

[†]Institute of Pharmacy, Faculty of Chemistry and Pharmacy, University of Regensburg, Universitätsstraße 31, D-93053 Regensburg, Germany

[‡]Institute of Chemistry, University of Tartu, Ravila 14a, 50411 Tartu, Estonia

[#]School of Life Sciences, University of Nottingham, Queen's Medical Centre, Nottingham NG7 2UH, United Kingdom

[§]Department of Chemistry and Pharmacy, Medicinal Chemistry, Friedrich Alexander University, Nikolaus-Fiebiger-Straße 10, D-91058 Erlangen, Germany

[||]Institute of Biophysics and Physical Biochemistry, Faculty of Biology and Preclinical Medicine, University of Regensburg, Universitätsstraße 31, D-93053 Regensburg, Germany

[⊥]Institute of Physical and Theoretical Chemistry, Faculty of Chemistry and Pharmacy, University of Regensburg, Universitätsstraße 31, D-93053 Regensburg, Germany

*e-mail addresses: ago.rinken@ut.ee
max.keller@ur.de
nicholas.holliday@nottingham.ac.uk

Content Page

1. Figures S1-S17 S3

2. Scheme S1 S18

3. Tables S1 and S2 S18

4. RP-HPLC chromatograms of **6-9**, **10a**, **10b** and **13-20** (purity controls) S19

5. ^{1}H-NMR spectra of compounds **6-9**, **10a**, **13** and **18**, and ^{13}C-NMR spectra of compounds **6-9** and **10a** in DMSO-d_6 and DMSO-d_6/D_2O 4:1 S24

6. References S36

1. Figures S1-S17

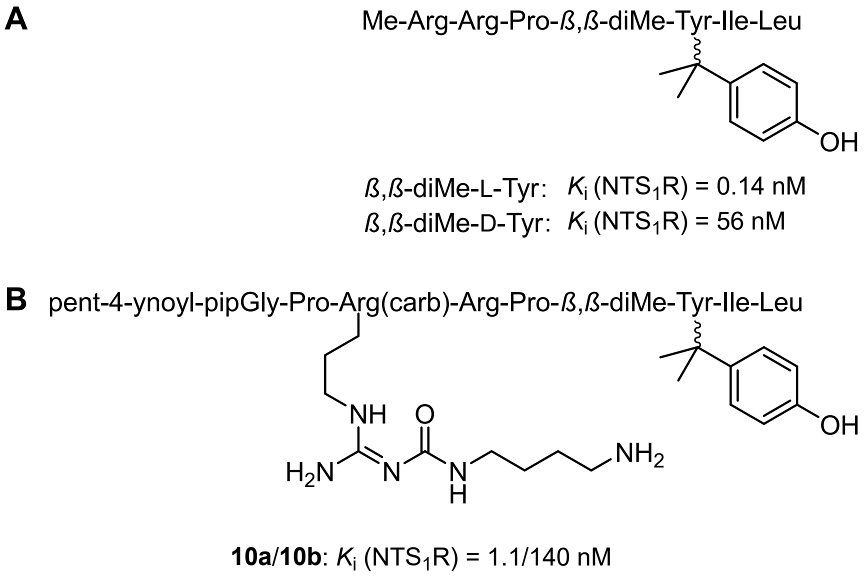

Figure S1: (A) Structures and NTS_1R binding affinities of reported NT(8-13) derivatives containing either β,β-dimethyl-L-tyrosine or β,β-dimethyl-D-tyrosine in position 11 (compound **48** and **49**, respectively, in Schindler et al.[1]). (B) Structures and NTS_1R binding affinities of **10a** and **10b**.

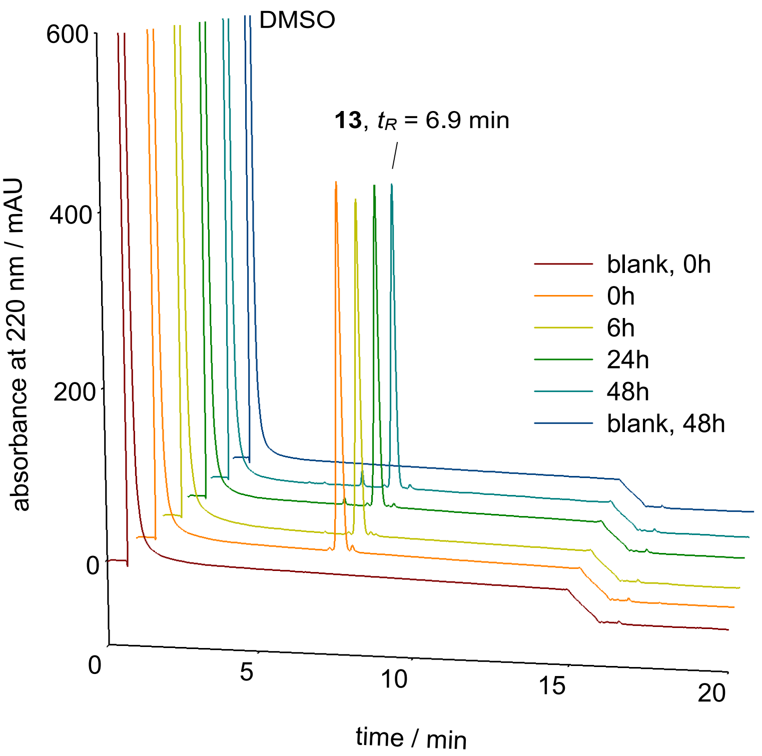

Figure S2. Investigation of the chemical stability of **13** in PBS (pH 7.4). Shown are the chromatograms of the RP-HPLC after incubation for up to 48 hours. **13** exhibited high stability. After 48 h, a minor decomposition product (t_R = 5.7 min) was observed.

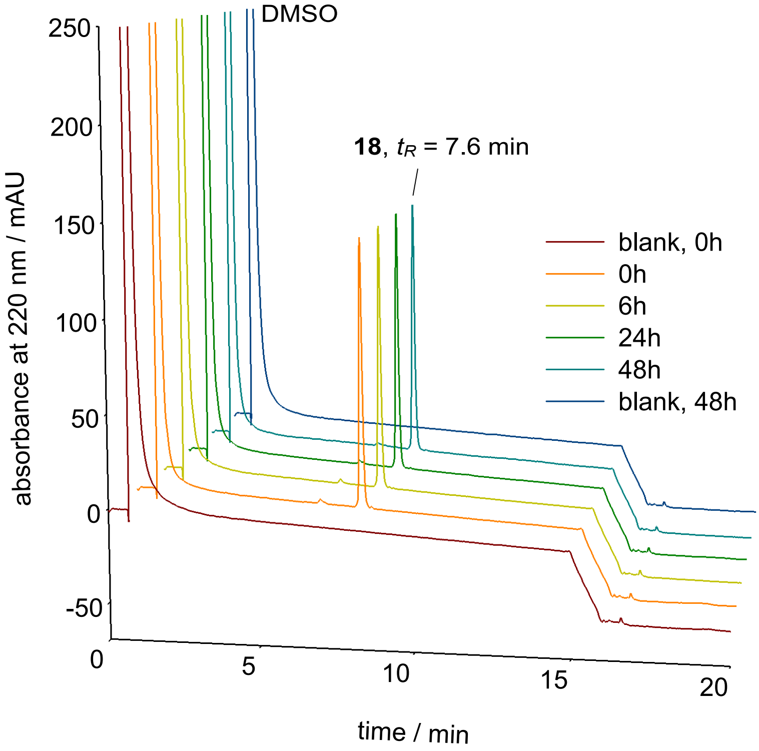

Figure S3. Investigation of the chemical stability of **18** in PBS (pH 7.4). Shown are the chromatograms of the RP-HPLC after incubation for up to 48 hours. **18** showed no decomposition.

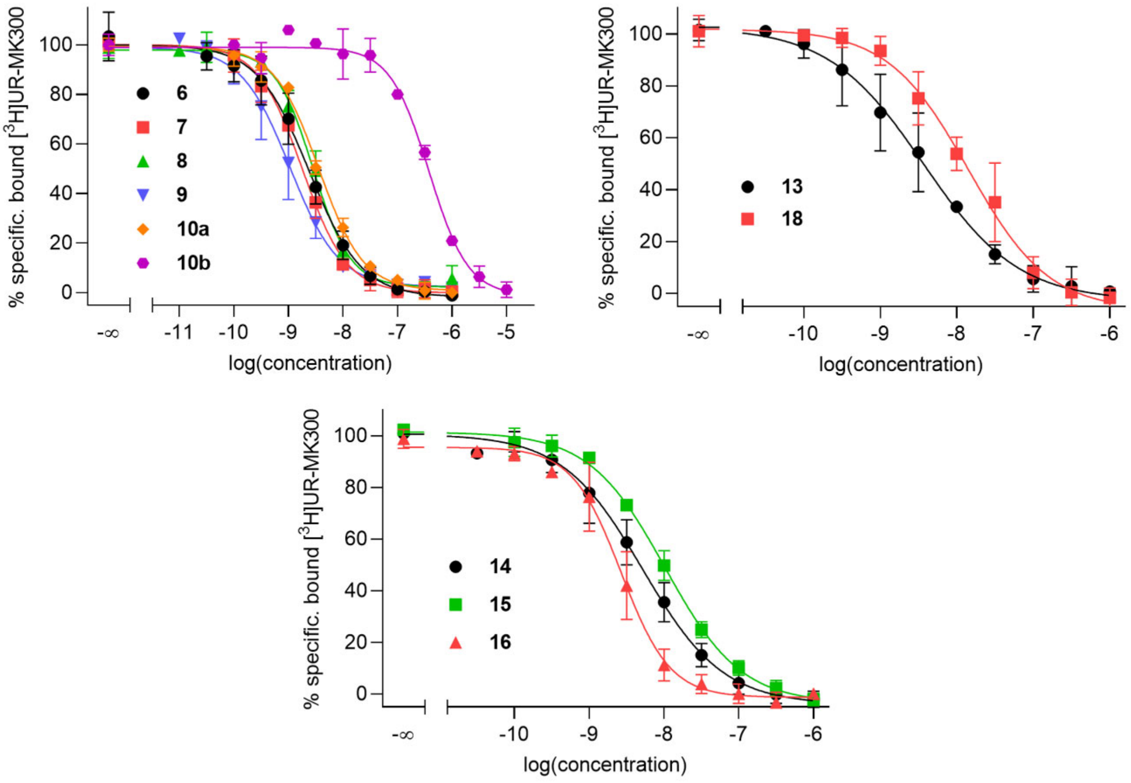

Figure S4. Radioligand displacement curves from competition binding experiments with [^{3}H]UR-MK300 (K_d = 0.41 nM,[1] c = 1 nM) and **6-9**, **10a**, **10b**, **13-16** or **18** at intact HT-29 cells. Data represent mean values ± SEM from at least three independent experiments performed in triplicate. For pK_i values, see Table 1 (main article).

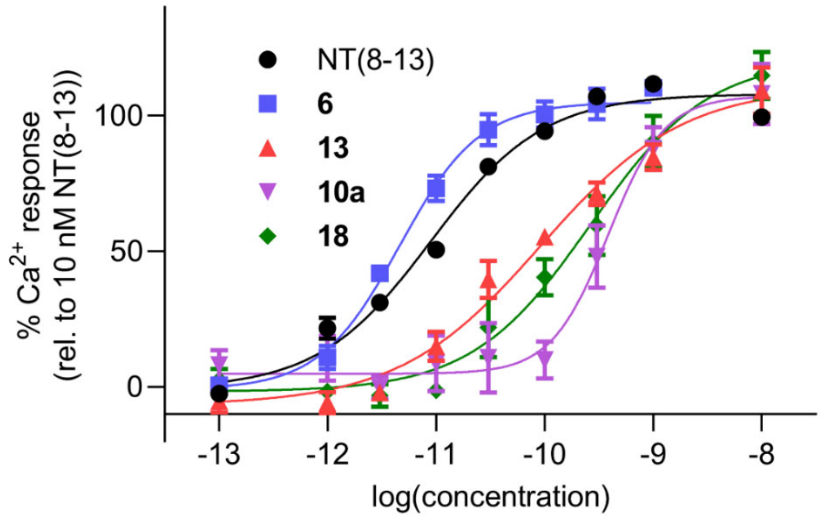

Figure S5. Concentration response curves of NT(8-13), **6**, **10a**, **13**, and **18**, obtained from a Fura-2 Ca^{2+} assay performed with CHO-hNTS$_1$R cells. Mean values ± SEM from at least three independent experiments (performed in triplicate).

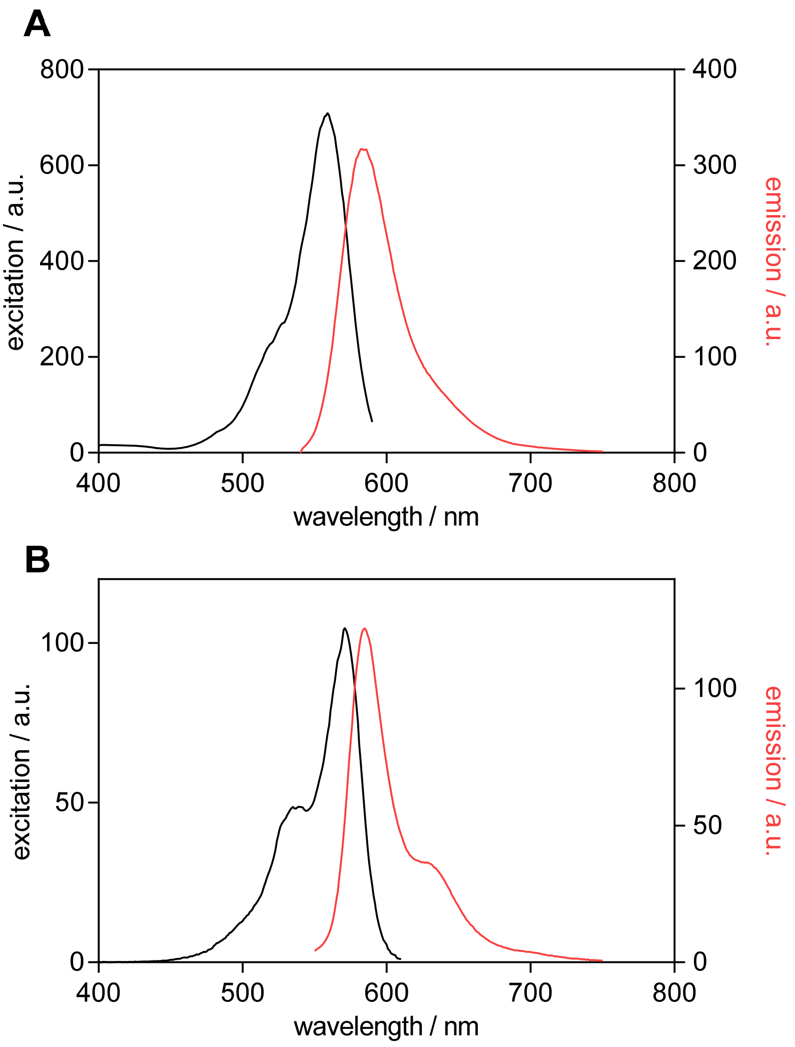

Figure S6. Excitation and corrected emission spectra of **13** (A) and **18** (B) recorded in PBS (pH 7.4) supplemented with 1% BSA, at 22 °C. The concentration of both ligands was 1 μM.

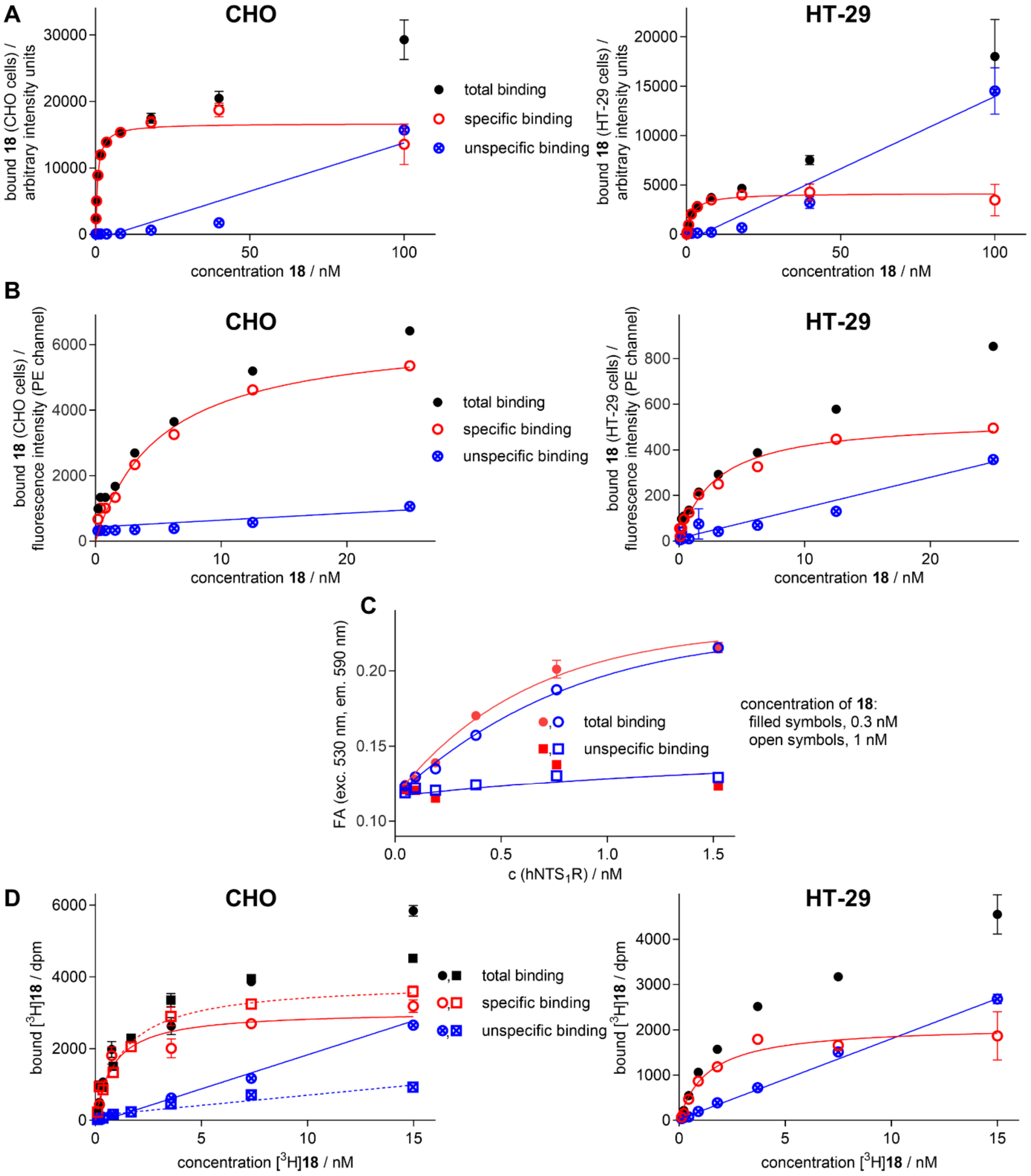

Figure S7. NTS_1R equilibrium binding of **18** and [^{3}H]**18** studied by different methods. (A) Binding isotherms (specific binding, open symbols) of **18** obtained from high-content imaging (HCI) binding experiments performed at intact CHO-hNTS_1R and HT-29 cells (incubation: 90 min at 23 °C). (B) Binding isotherms (specific binding, open symbols) of **18** obtained from flow cytometric (FC) saturation binding experiments performed at intact CHO-hNTS_1R and HT-29 cells (incubation: 90 min at 23 °C). (C) Binding isotherms (total binding, circles) of **18** obtained from FA-based binding experiments using fixed concentrations of **18** (0.3 or 1 nM) and increasing amounts of NTS_1R-displaying BBVs (depicted data represent snapshots at 10 min incubation at 27 °C). (D) Binding isotherms of [^{3}H]**18** from radiochemical saturation binding experiments performed at intact CHO-hNTS_1R cells (adherent and in suspension) and at adherent HT-29 cells (incubation: 90 min at 23 °C). Circles represent adherent cells, squares represent suspended cells. Unspecific binding was determined in the presence of 1 µM NT(8-13) (A, B and D) or 1 µM SR142948 (C). K_d values are presented in Table 3. Data represent mean values ± SEM (total and unspecific binding) or calculated values ± propagated error (specific binding) from representative experiments performed in triplicate (A, B and D) or duplicate (C).

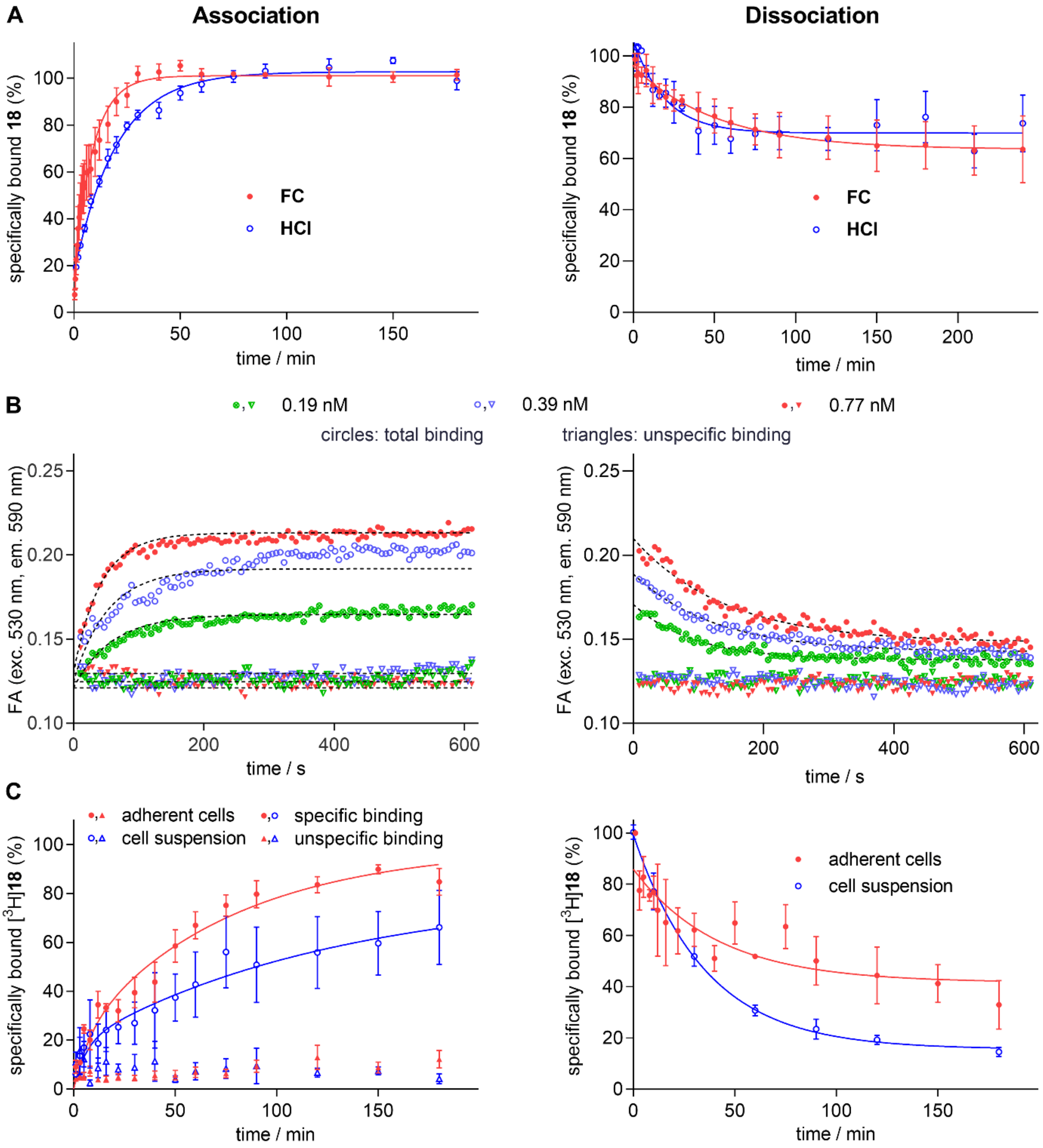

Figure S8. Binding kinetics of **18** and [^{3}H]**18** at NTS_1R studied by different methods. (A) Association and dissociation of **18** studied by HCI and FC at live adherent CHO-hNTS_1R cells (HCI) or suspended CHO-hNTS_1R cells (FC) at 23 °C. Concentrations of **18** used for the association: 2.5 nM (HCI) and 1 nM (FC); concentrations of **18** used during the preincubation period (90 min) of dissociation experiments: 2.5 nM (HCI) and 10 nM (FC). (B) Association and dissociation of **18** (0.3 nM) determined in an FA-based assay for three different NTS_1R concentrations (green, blue, and red symbols) at 27 °C using NTS_1R-displaying BBVs. Total binding is represented by circles and unspecific binding is represented by triangles. Following the association for up to 180 min did not reveal a second association phase (data not shown). (C) Association and dissociation of [^{3}H]**18** studied at live adherent CHO-hNTS_1R cells and suspended CHO-hNTS_1R cells at 23 °C. Concentrations of [^{3}H]**18** used for the association: 1.1 nM (adherent cells) and 1.2 nM (cell suspension); concentrations of [^{3}H]**18** used during the preincubation period (90 min) of dissociation experiments: 5.5 nM (adherent cells) and 5 nM (cell suspension). For the biphasic association, unspecific binding is shown to demonstrate that both association phases account for binding to NTS_1R. Proportion of fast/slow kinetic components (association): 16%/84% (adherent cells), 19%/81% (suspended cells). In the case of dissociation experiments performed with suspended cells, less times were studied because these experiments were laborious due to the use of 50 mL falcon tubes instead of 96-well plates (in this case, a separate work-up process with the cell harvester had to be carried out for each timepoint).
Dissociation and association rate constants are presented in Table 3. Data represent mean values ± SEM from at least three independent experiments performed in triplicate (A (HCI), C (association, dissociation at adherent cells)) or duplicate (A (FC), B, C (dissociation with cell suspensions)).

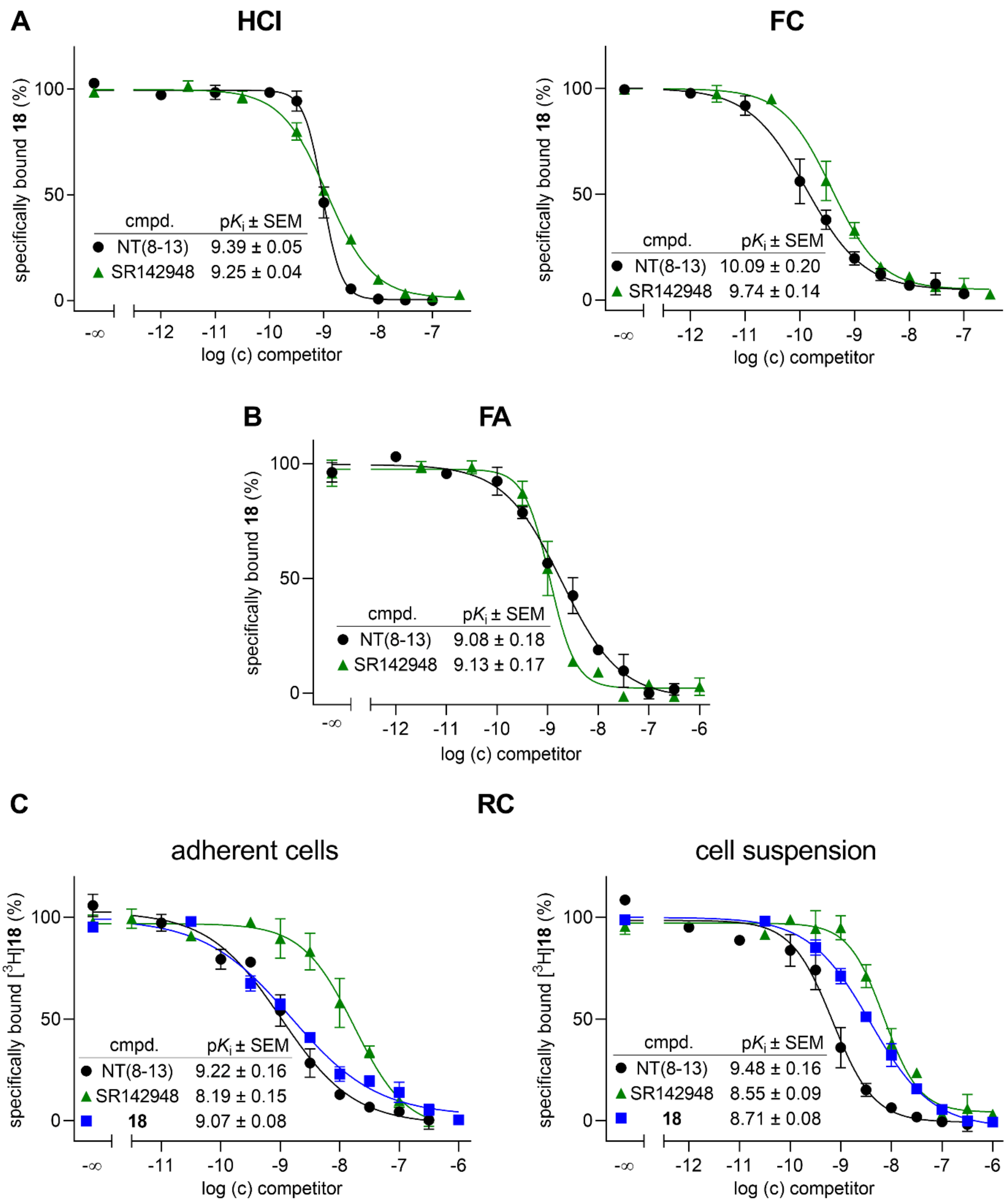

Figure S9. Displacement curves and corresponding pK_i values from competition binding studies performed with **18** or [^{3}H]**18** and NT(8-13) and SR142948 using different types of binding assays (A: high-content imaging and flow cytometry; B: fluorescence anisotropy; C: radiochemical binding assay). Used concentrations of **18**: 1.3 nM (A (HCI)), 3.1 nM (A (FC)), 0.3 nM (B). Used concentrations of [^{3}H]**18** (C): 1.1 nM (adherent cells), 1.2 nM (cell suspension). Incubation times: 90 min (A, B, C). Incubation temperatures: 23 °C (A, C), 27 °C (B). Data represent mean value ± SEM from at least three individual experiments performed in triplicate.

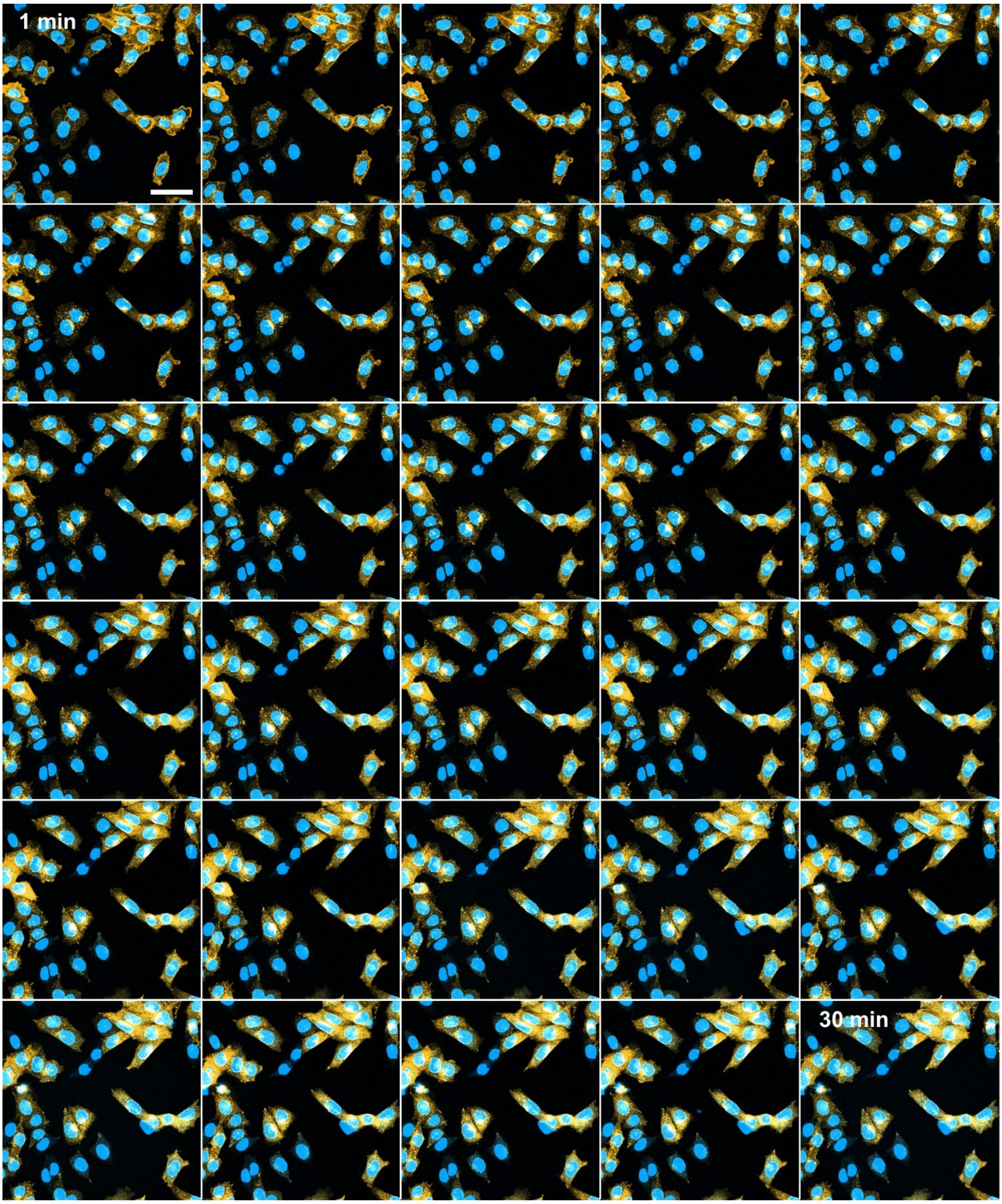

Figure S10. Fluorescence images (acquired with a Zeiss Celldiscoverer 7) of a binding experiment performed with the fluorescent NT(6-13) derivative **13** (5 nM) at intact CHO-hNTS$_1$R cells (temperature: 23 °C). 30 images were acquired over a period of 30 min. Shown are merge fluorescence of **13** (orange) and nuclei (blue). Scale bar: 50 µm. Unspecific binding of this experiment is shown in Figure S12.

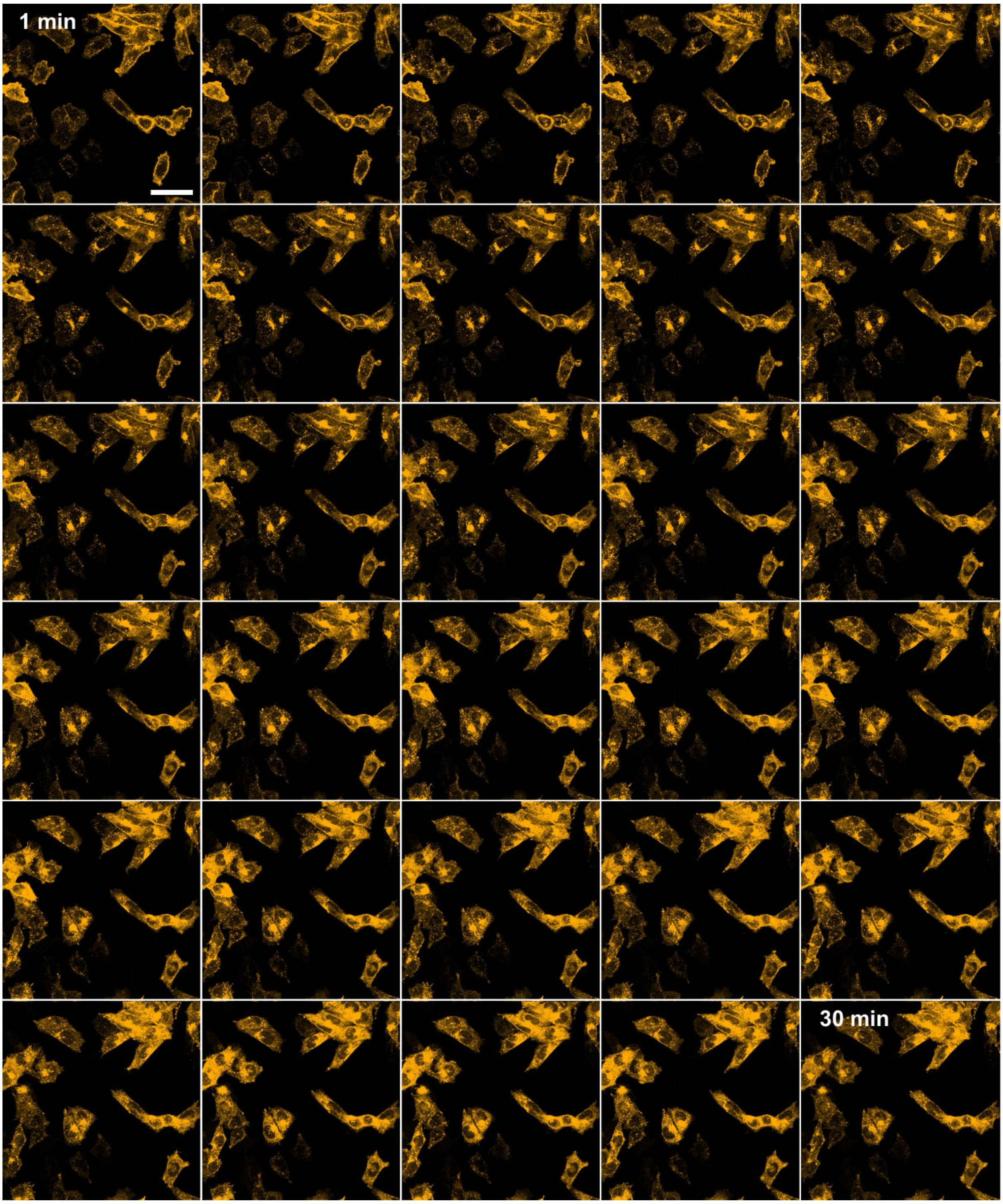

Figure S11. Same images as shown in Figure S10, but without nuclei fluorescence.

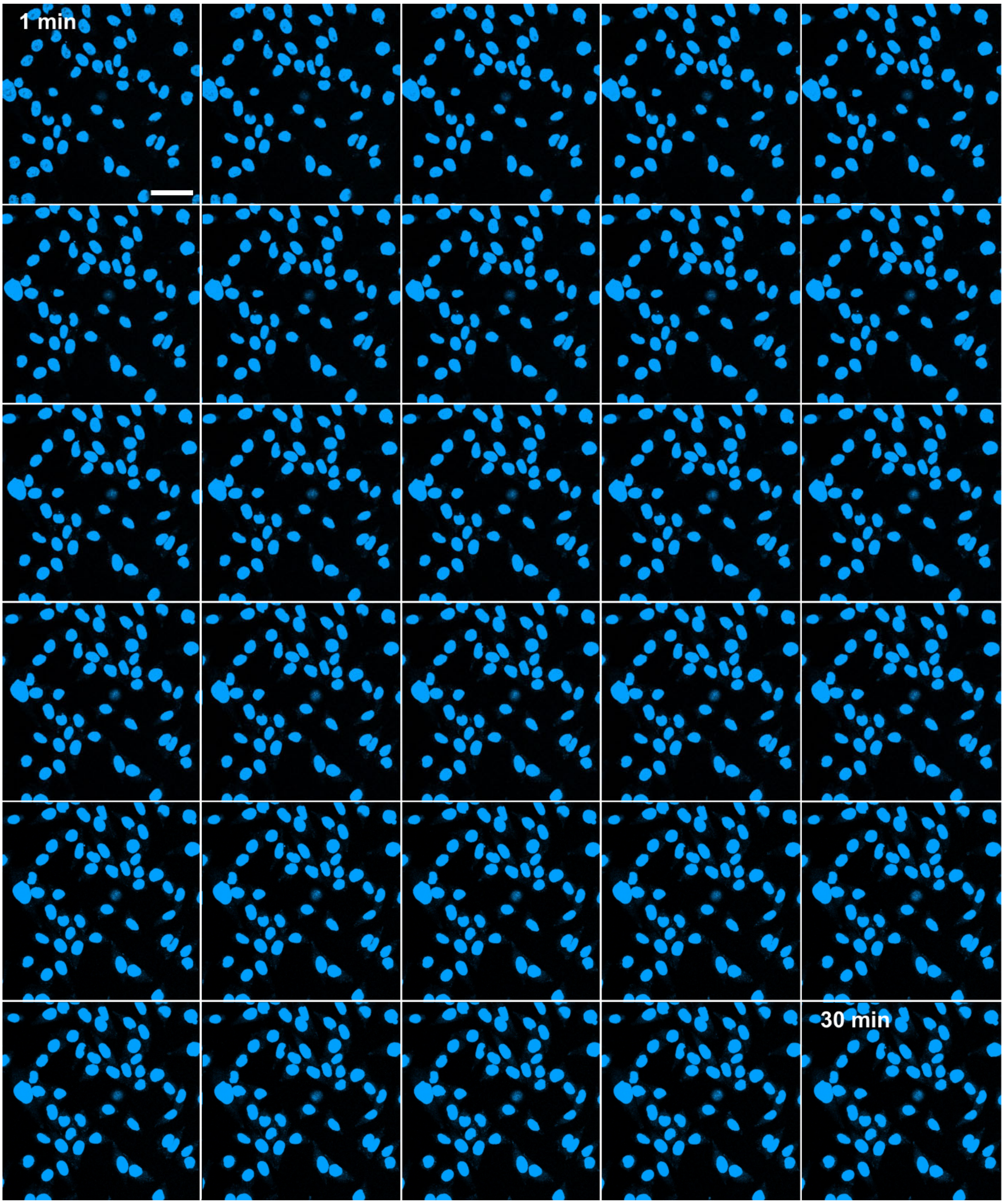

Figure S12. Fluorescence images (acquired with a Zeiss Celldiscoverer 7) of adherent CHO-hNTS$_1$R cells, which were incubated with **13** (5 nM) in the presence of 1 µM NT(8-13) at 23 °C (determination of unspecific binding). 30 images were acquired over a period of 30 min. Shown are merge fluorescence of **13** (orange) and nuclei (blue). Scale bar: 50 µm. Total binding of this experiment is shown in Figures S10 and S11.

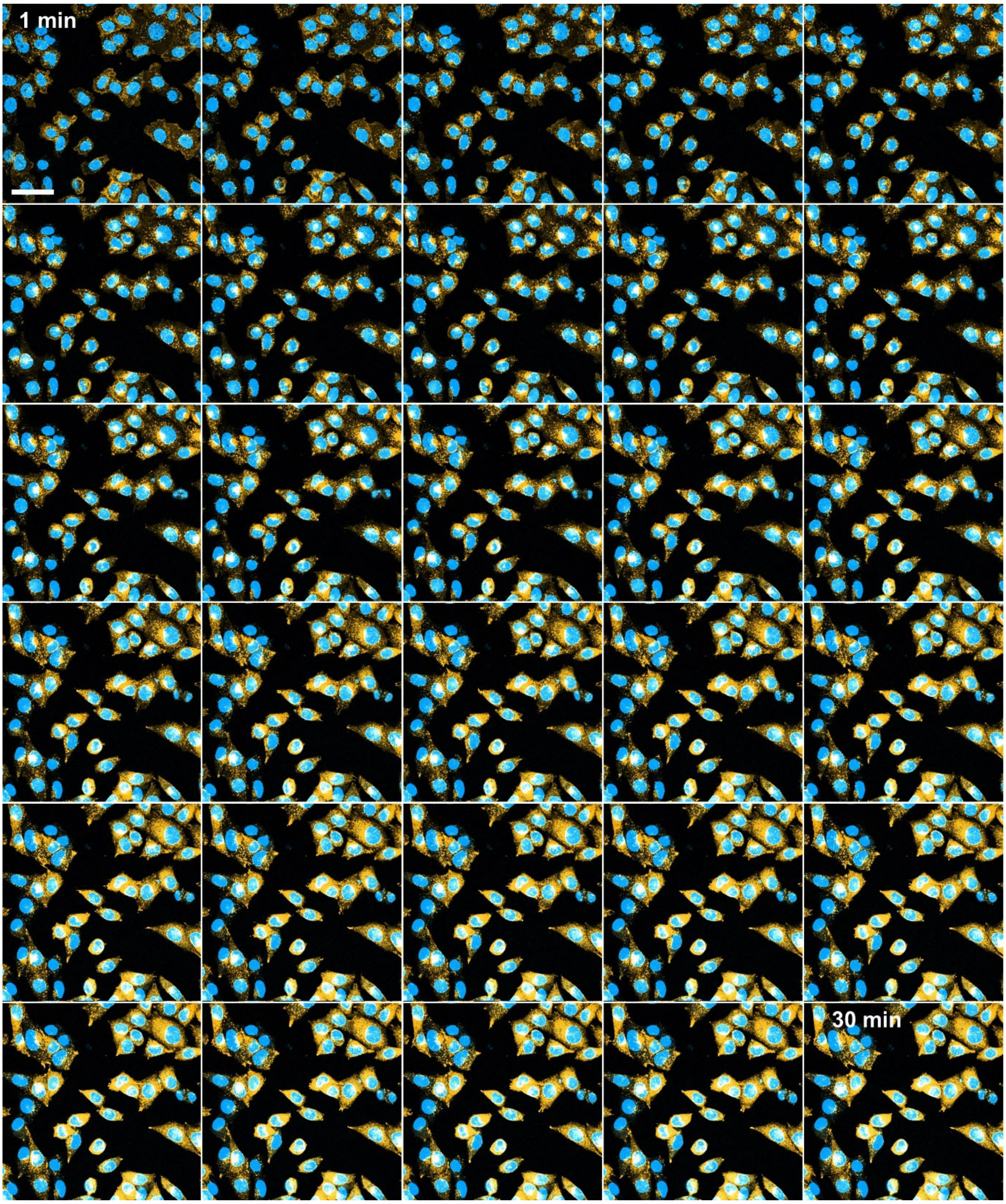

Figure S13. Fluorescence images (acquired with a Zeiss Celldiscoverer 7) of a binding experiment performed with the fluorescent NT(6-13) derivative **18** (5 nM) at intact CHO-hNTS$_1$R cells (temperature: 23 °C). 30 images were acquired over a period of 30 min. Shown are merge fluorescence of **18** (orange) and nuclei (blue). Scale bar: 50 µm. Unspecific binding of this experiment is shown in Figure S15.

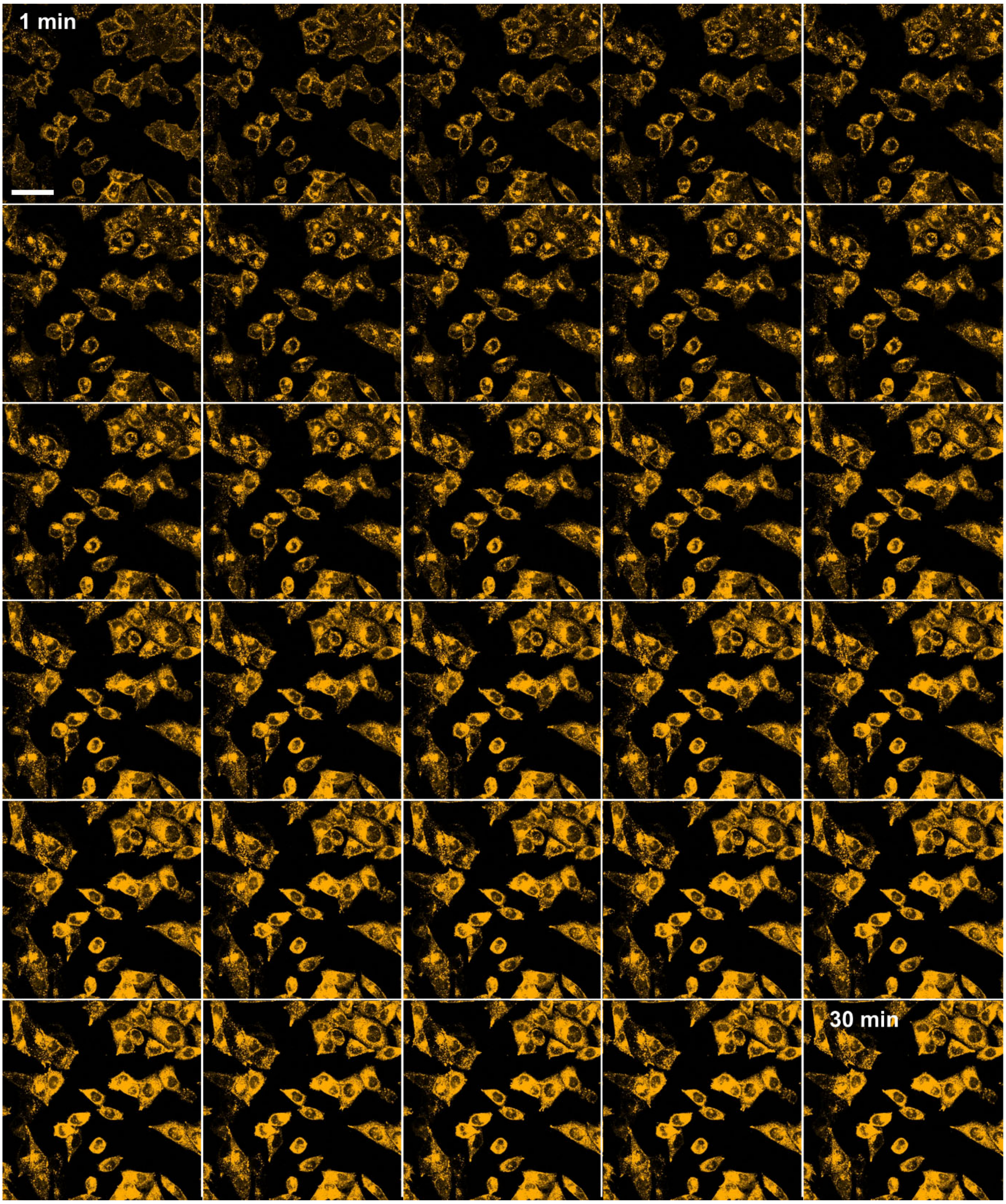

Figure S14. Same images as shown in Figure S13, but without nuclei fluorescence.

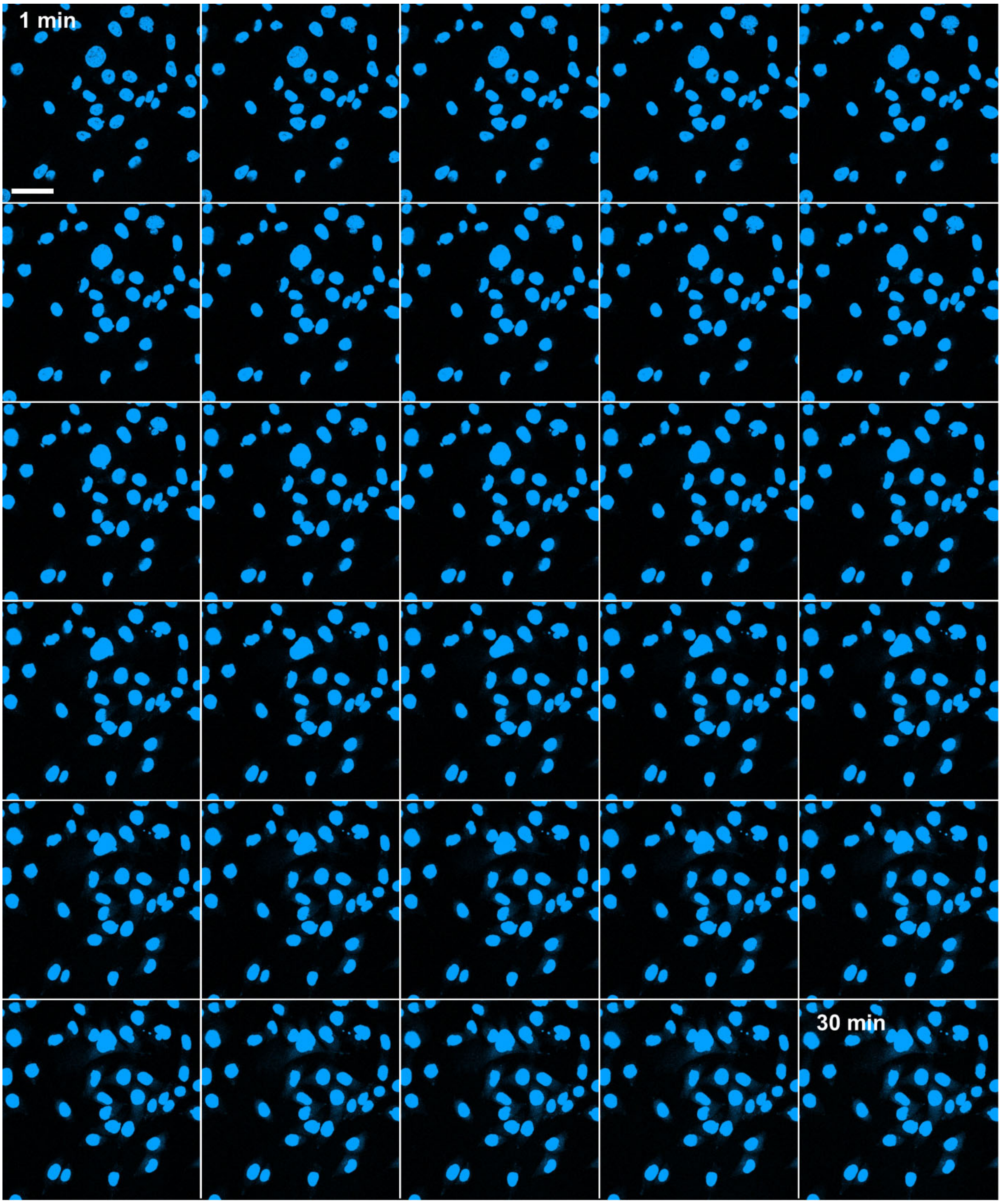

Figure S15. Fluorescence images (acquired with a Zeiss Celldiscoverer 7) of adherent CHO-hNTS$_1$R cells, which were incubated with **18** (5 nM) in the presence of 1 µM NT(8-13) at 23 °C (determination of unspecific binding). 30 images were acquired over a period of 30 min. Shown are merge fluorescence of **18** (orange) and nuclei (blue). Scale bar: 50 µm. Total binding of this experiment is shown in Figures S13 and S14.

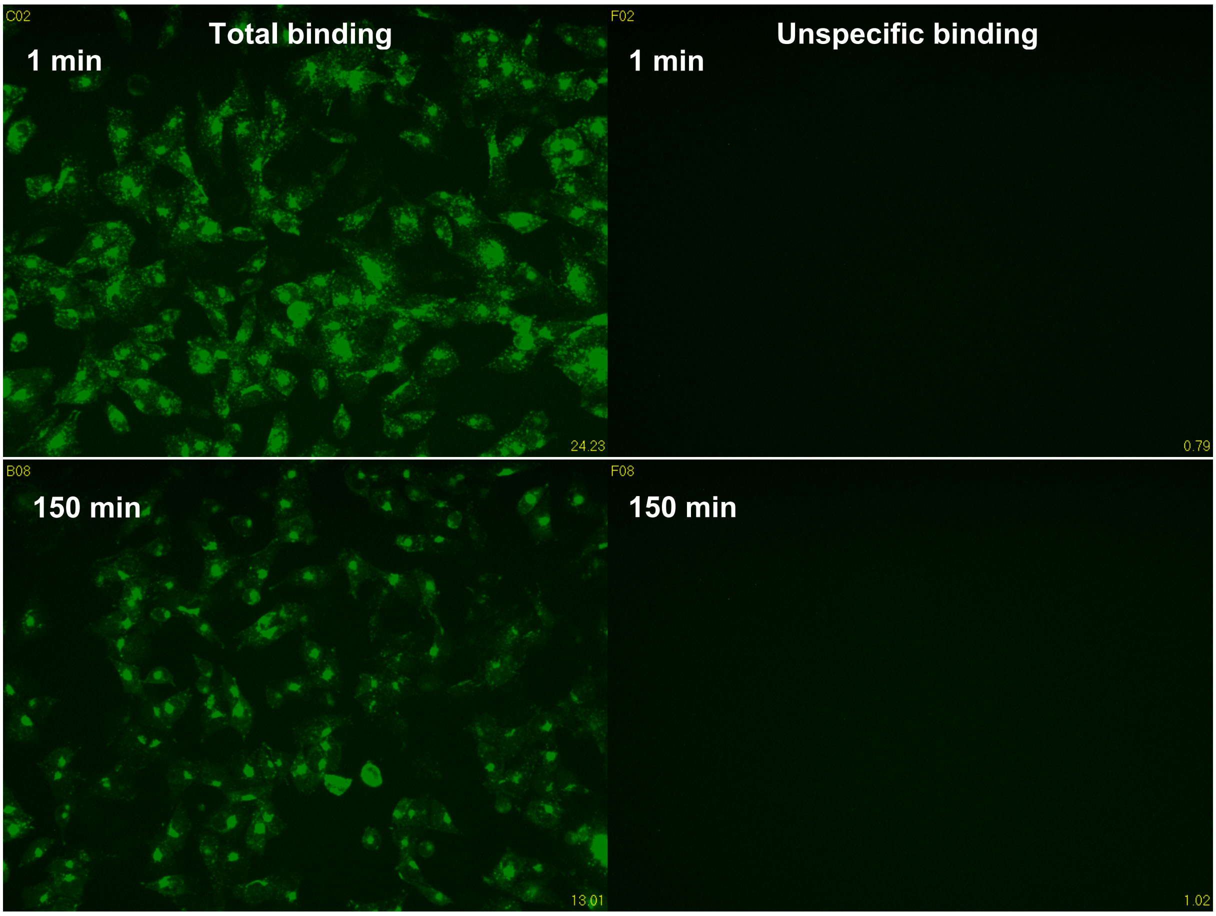

Figure S16. Representative fluorescence images acquired with an IX Micro Confocal plate reader of a high-content imaging dissociation experiment performed with **13** at intact adherent CHO-hNTS$_1$R cells in a 96-well plate at 22 ± 1 °C. Unspecific binding was determined in the presence of 1 µM NT(8-13). Shown are representative wells after 1 min and 150 min of dissociation. Fluorescence detected at 150 min represents the plateau of the dissociation curve shown in Figure 4A (main article).

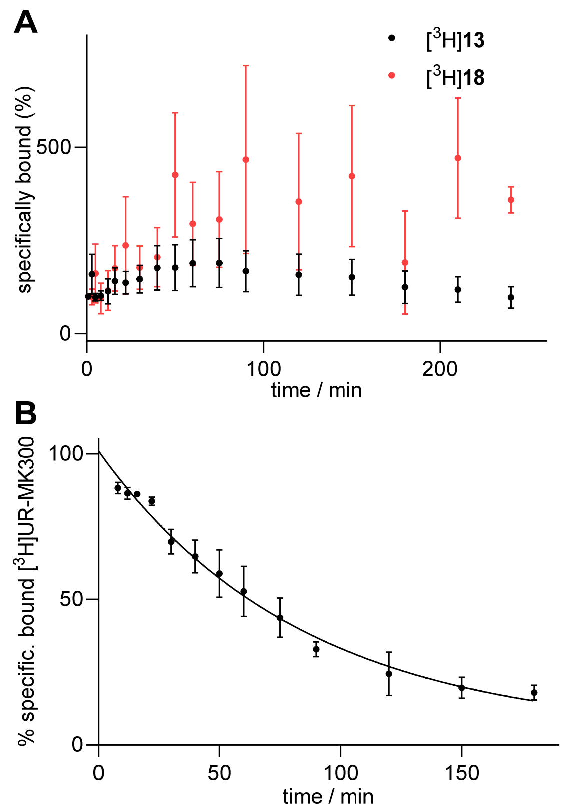

Figure S17. Data obtained from dissociation experiments performed with [^{3}H]**13**, [^{3}H]**18** and [^{3}H]UR-MK300 in 96-well PP plates at CHO-hNTS$_1$R cells suspended in D-PBS with 1% BSA. All experiments were performed at 23 ± 1 °C. The dissociation was initiated by the addition of NT(8-13) (final concentration: 25 µM). Unspecific binding (not shown) was determined by the addition of 1 µM NT(8-13) during the preincubation. (A) Data obtained for [^{3}H]**13** and [^{3}H]**18**. Used radioligand concentrations during the preincubation period (90 min): 5 or 9 nM ([^{3}H]**13**) and 5 or 6 nM ([^{3}H]**18**). Data represent mean values ± SEM from nine independent experiments performed in triplicate (carried out by three different operators). The first timepoint of each experiment (1 min) was set to 100 %. The results of the individual experiments were not reproducible and gave no trend. (B) Dissociation of [^{3}H]UR-MK300. The radioligand concentration used for the preincubation period (90 min) was 2 nM. Data represent mean values ± SEM from three independent experiments performed in triplicate. The fit (one phase decay, GraphPad Prism 5) was extrapolated to t = 0 min.

2. Scheme S1

Scheme S1. Synthesis of the azido-functionalized fluorescent dye **17**.

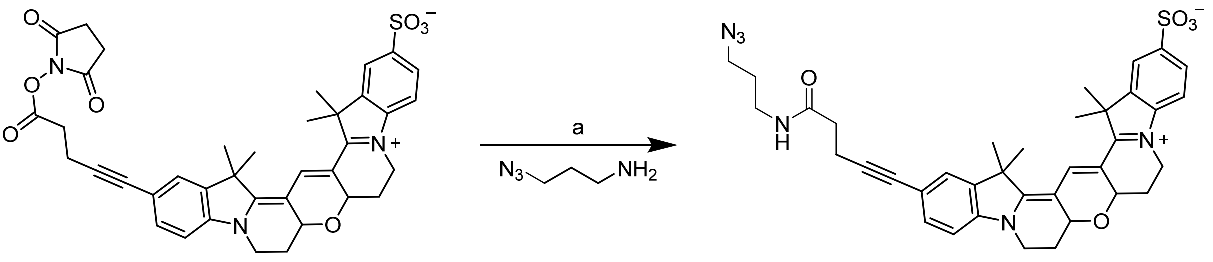

21 **17**

Reagents and conditions: (a) DIPEA, DMF, room temperature (rt), 1 h, 54%.

3. Tables S1 and S2

Table S1. Stabilities of **13** and **18** in human plasma/PBS 1:2 v/v (37 °C).

	% intact compound in plasma after the specified incubation times				
compd.	30 min	2 h	6 h	24 h	48 h
**13**	> 99	> 99	> 99	86 ± 4	58 ± 5
**18**	n.d.	95 ± 6	90 ± 6	82 ± 4	52 ± 4

Table S2. Recoveries of **13** and **18** from human plasma/PBS 1:2 v/v and ratios of compound-recovery over recovery of internal standard (1-methyl-D-tryptophan).

compd.	recovery compd. (%)[a]	recovery 1-methyl-D-tryptophan (%)[a]	ratio[b]
	75	80	0.93
	102	98	1.04
**13**	109	91	1.11
	104	98	1.06
			(1.03 ± 0.03)
	98	108	0.91
	72	71	1.01
**18**	65	63	1.03
	70	69	1.01
			(0.99 ± 0.02)

[a]Recoveries of the peptides and of the internal standard from human plasma/PBS 1:2 v/v using a peptide concentration of 80 μM and an internal standard concentration of 10 μM (four independent experiments). [b]Ratios of peptide recovery over recovery of internal standard calculated for individual experiments, as well as mean recovery ratios ± SEM (given in parenthesis).

4. RP-HPLC chromatograms of 6-9, 10a, 10b and 13-20 (purity controls)

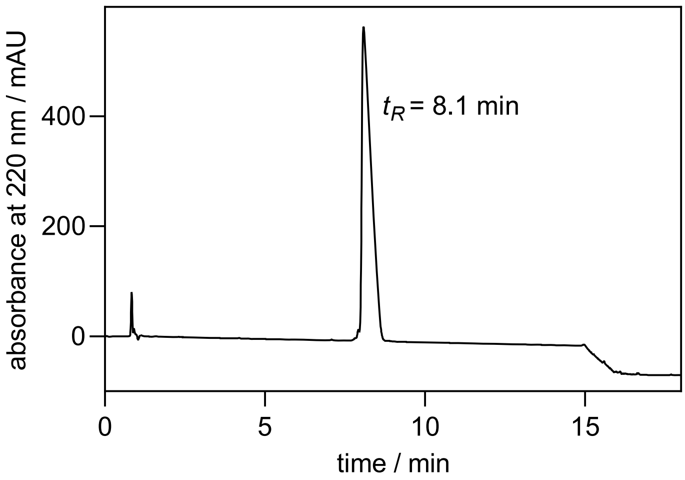

RP-HPLC analysis of compound **6**

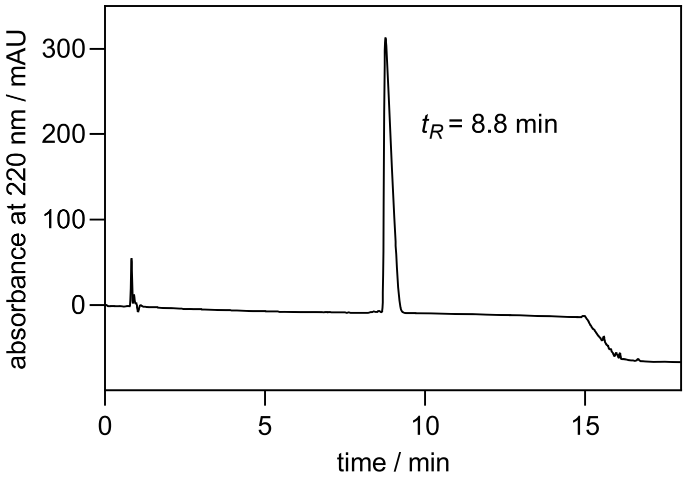

RP-HPLC analysis of compound **7**

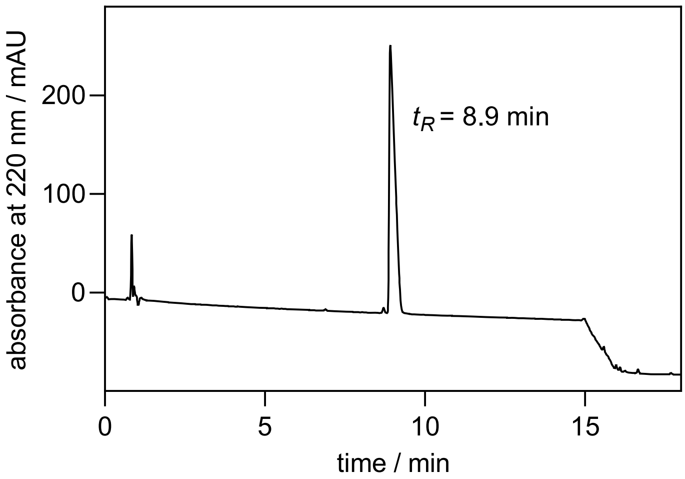

RP-HPLC analysis of compound **8**

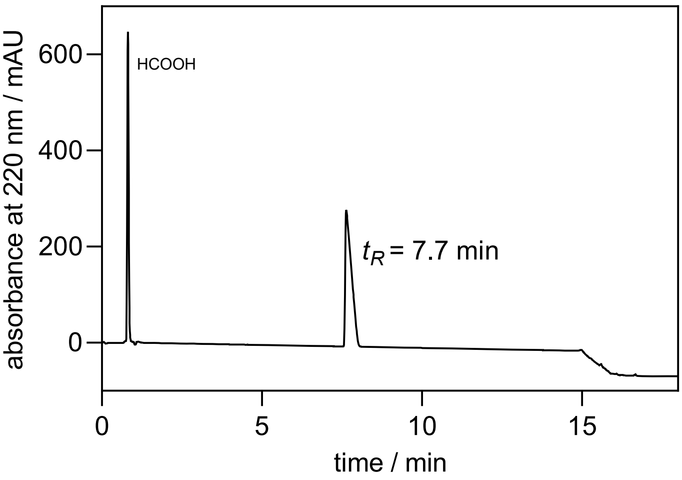

RP-HPLC analysis of compound **9**

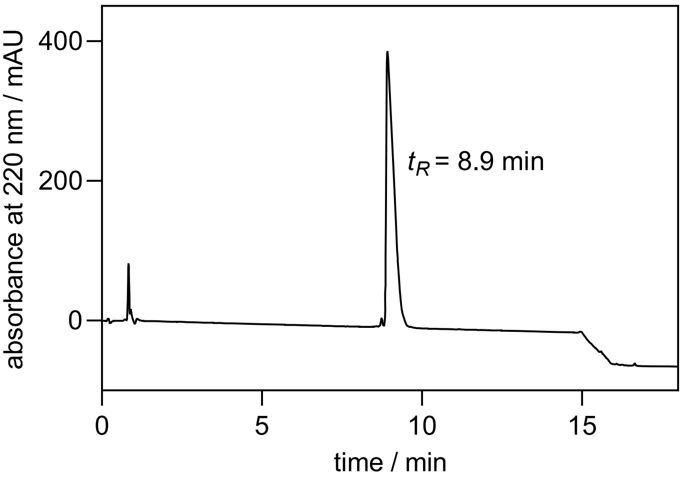

RP-HPLC analysis of compound **10a**

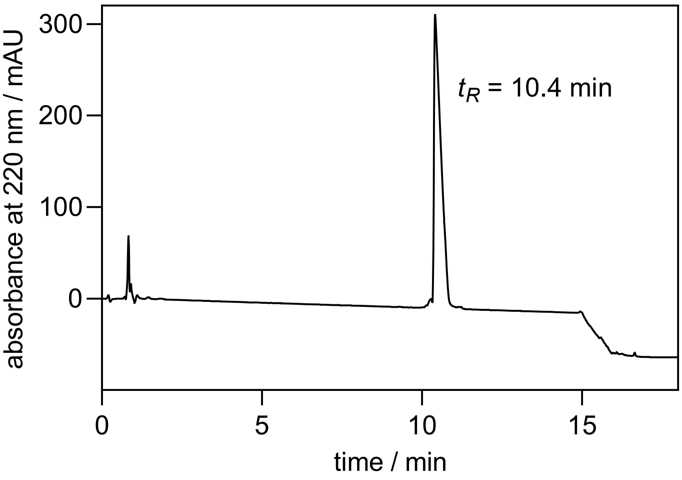

RP-HPLC analysis of compound **10b**

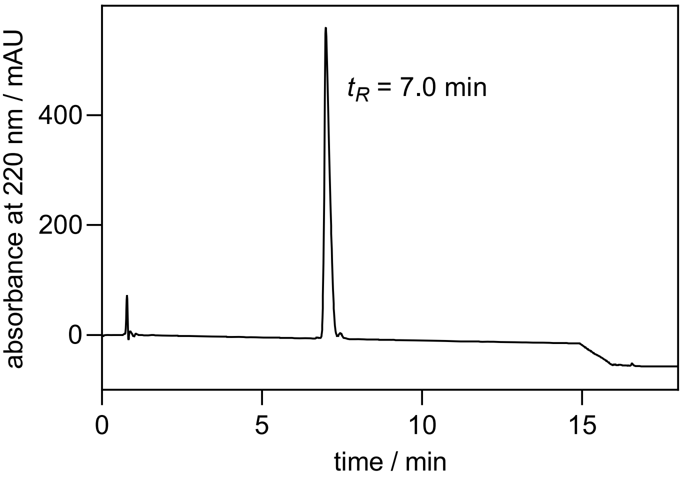

RP-HPLC analysis of compound **13**

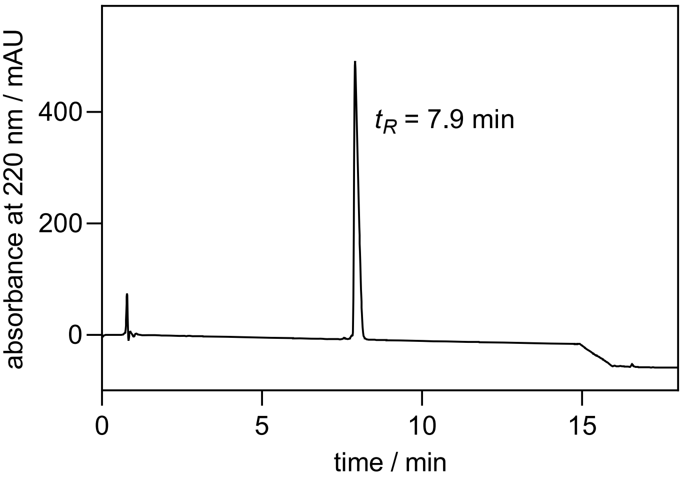

RP-HPLC analysis of compound **14**

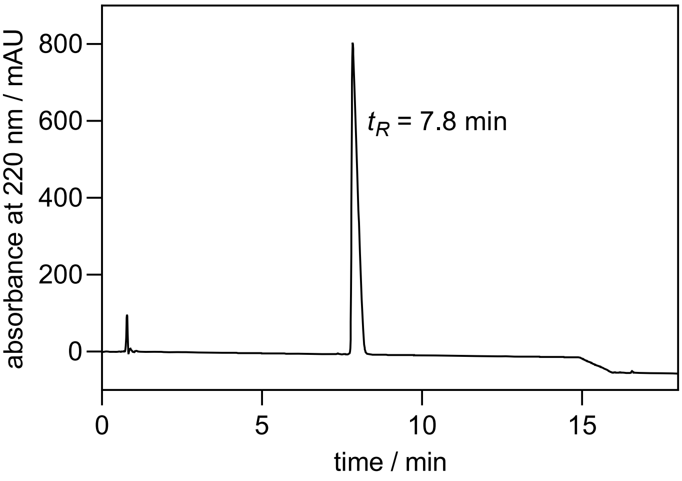

RP-HPLC analysis of compound **15**

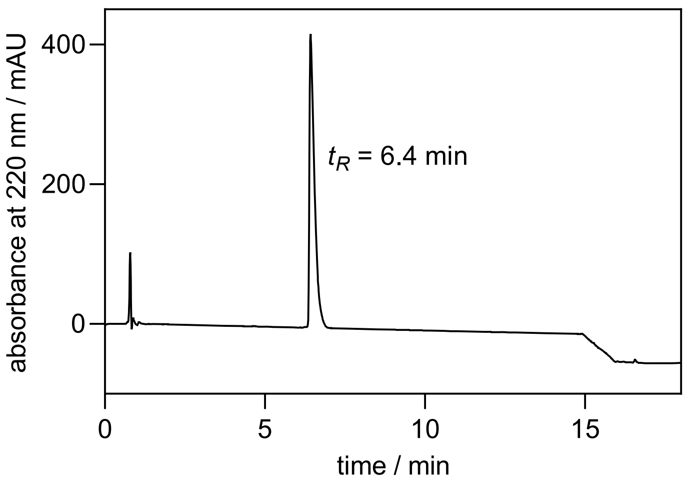

RP-HPLC analysis of compound **16**

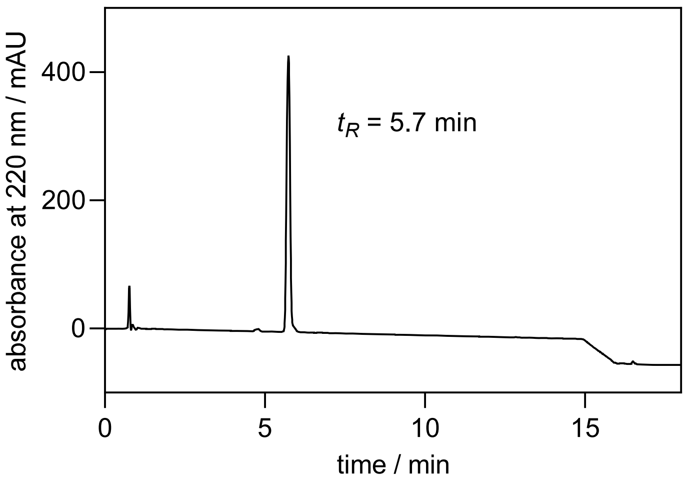

RP-HPLC analysis of compound **17**

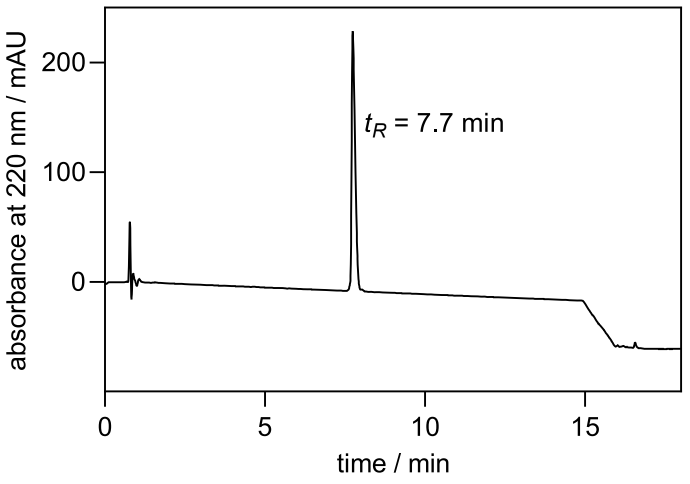

RP-HPLC analysis of compound **18**

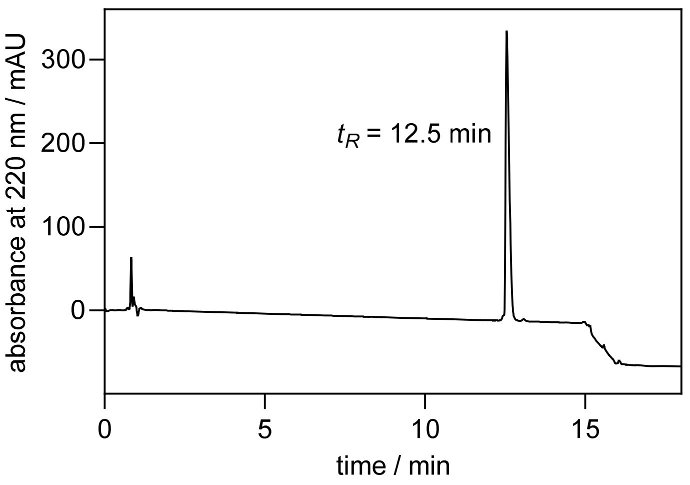

RP-HPLC analysis of compound **19**

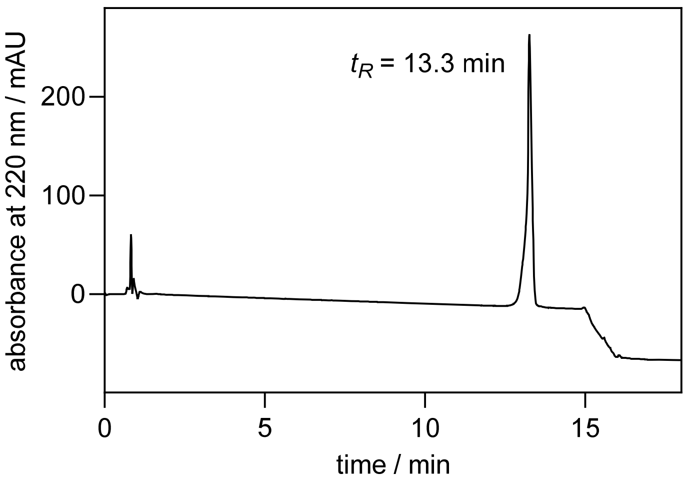

RP-HPLC analysis of compound **20**

5. ^{1}H-NMR spectra of compounds 6-9, 10a, 13 and 18, and ^{13}C-NMR spectra of compounds 6-9 and 10a in DMSO-d_6 and DMSO-d_6/D_2O 4:1

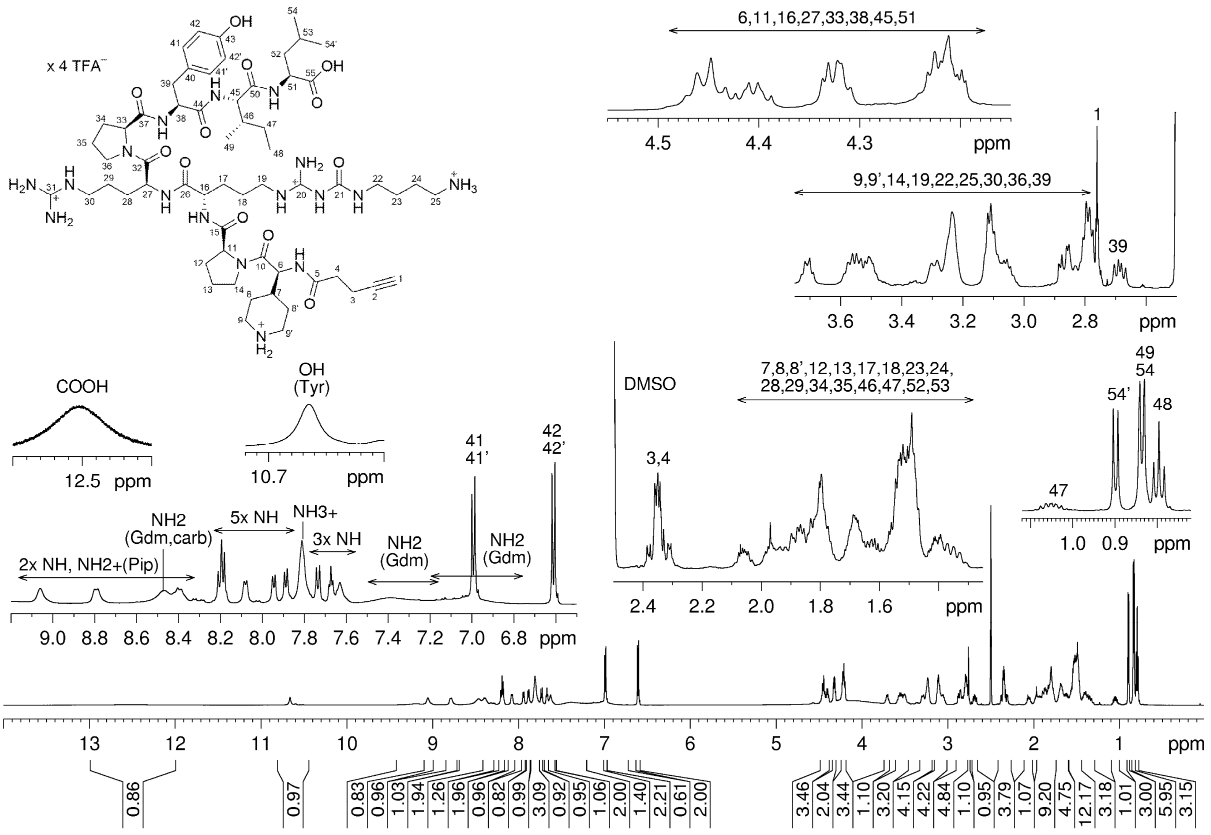

^{1}H-NMR spectrum (600 MHz, DMSO-d_6) of compound **6**

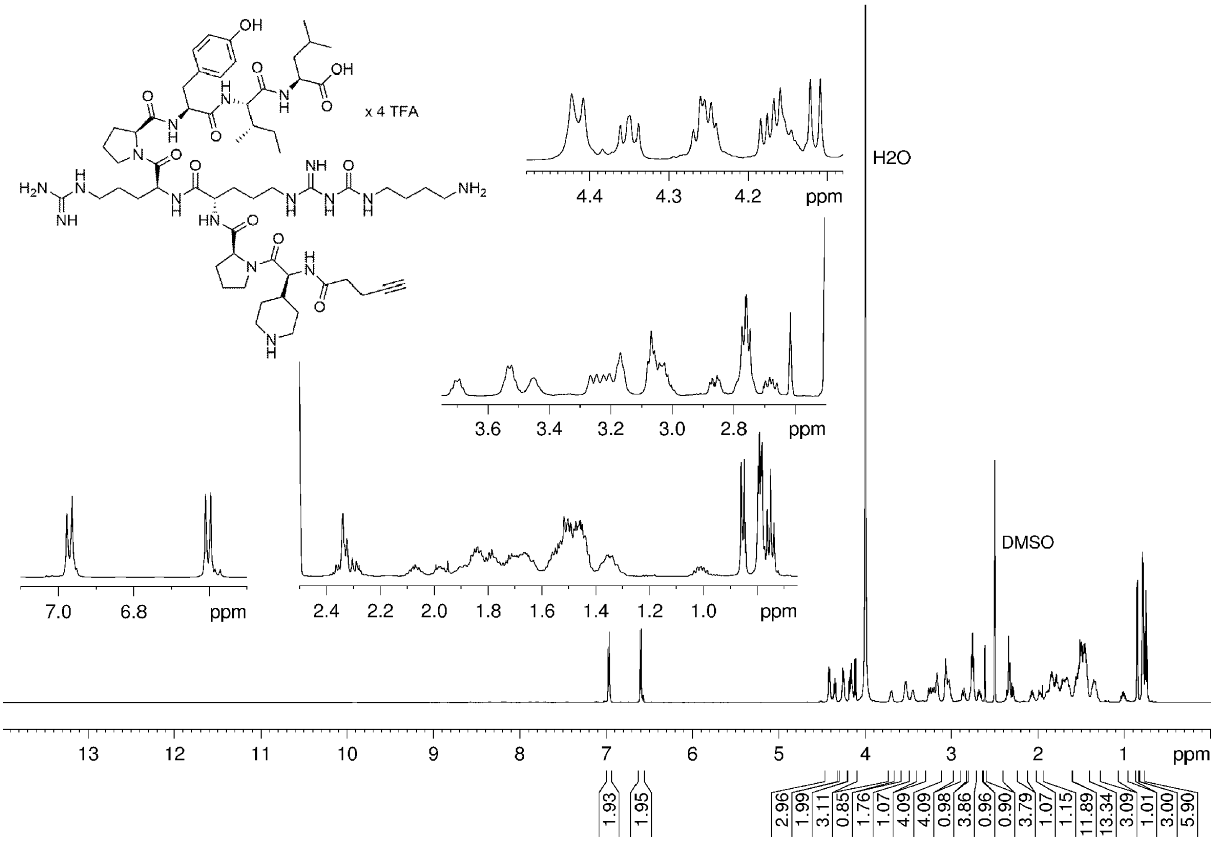

^{1}H-NMR spectrum (600 MHz, DMSO-d_6/D_2O 4:1 v/v) of compound **6**

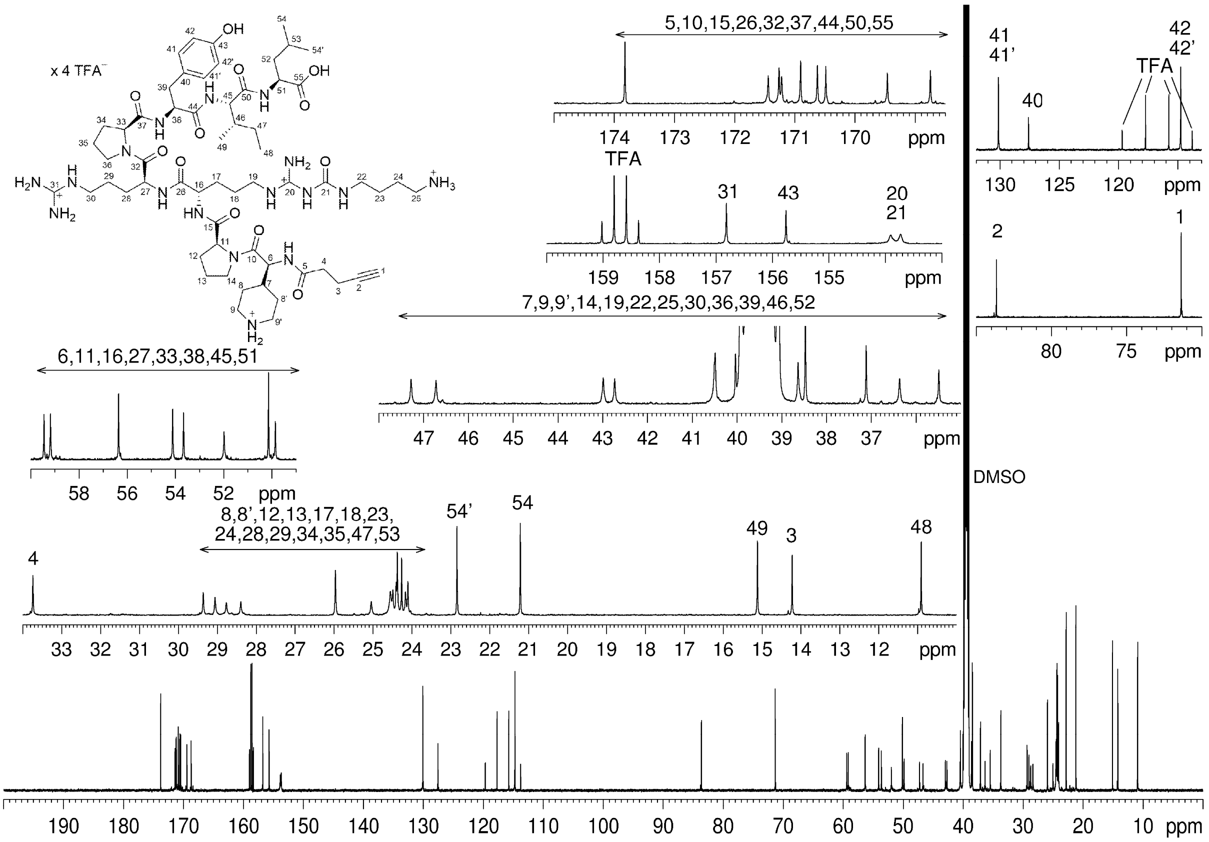

^{13}C-NMR spectrum (150 MHz, DMSO-d$_6$) of compound **6**

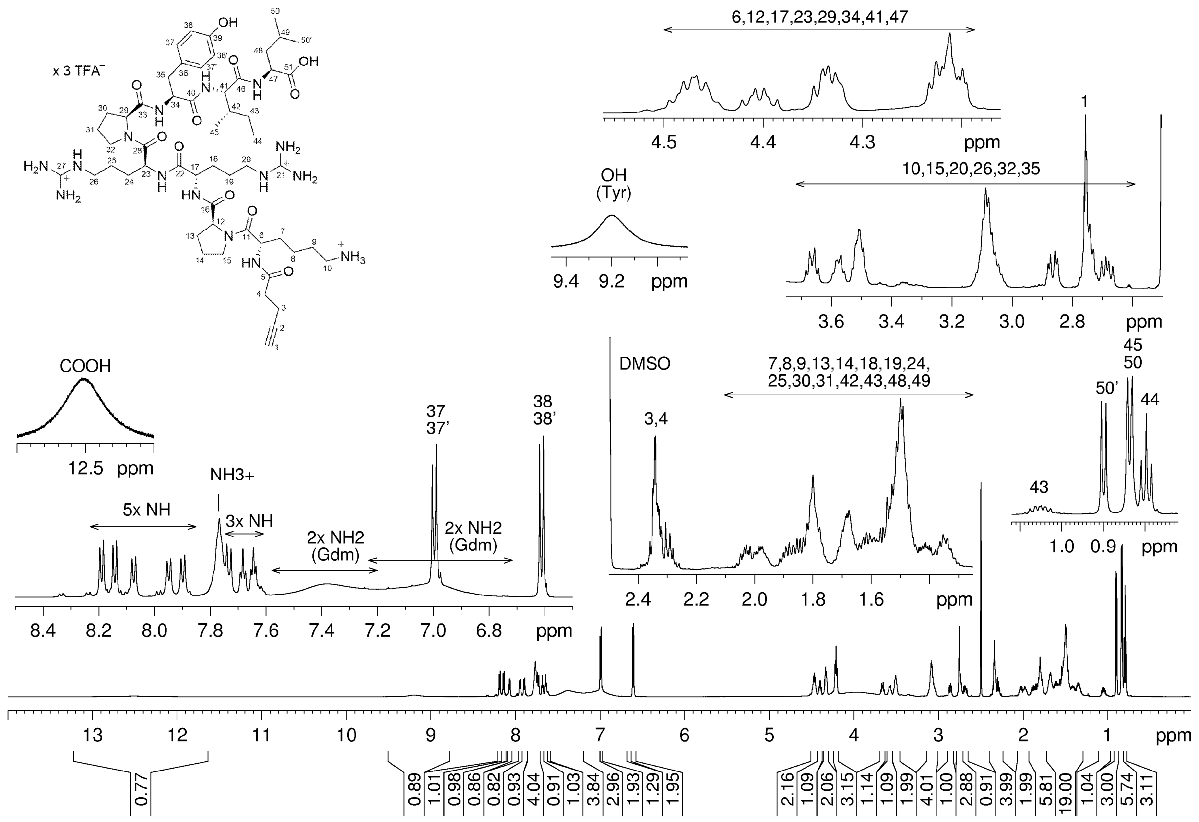

^{1}H-NMR spectrum (600 MHz, DMSO-d$_6$) of compound **7**

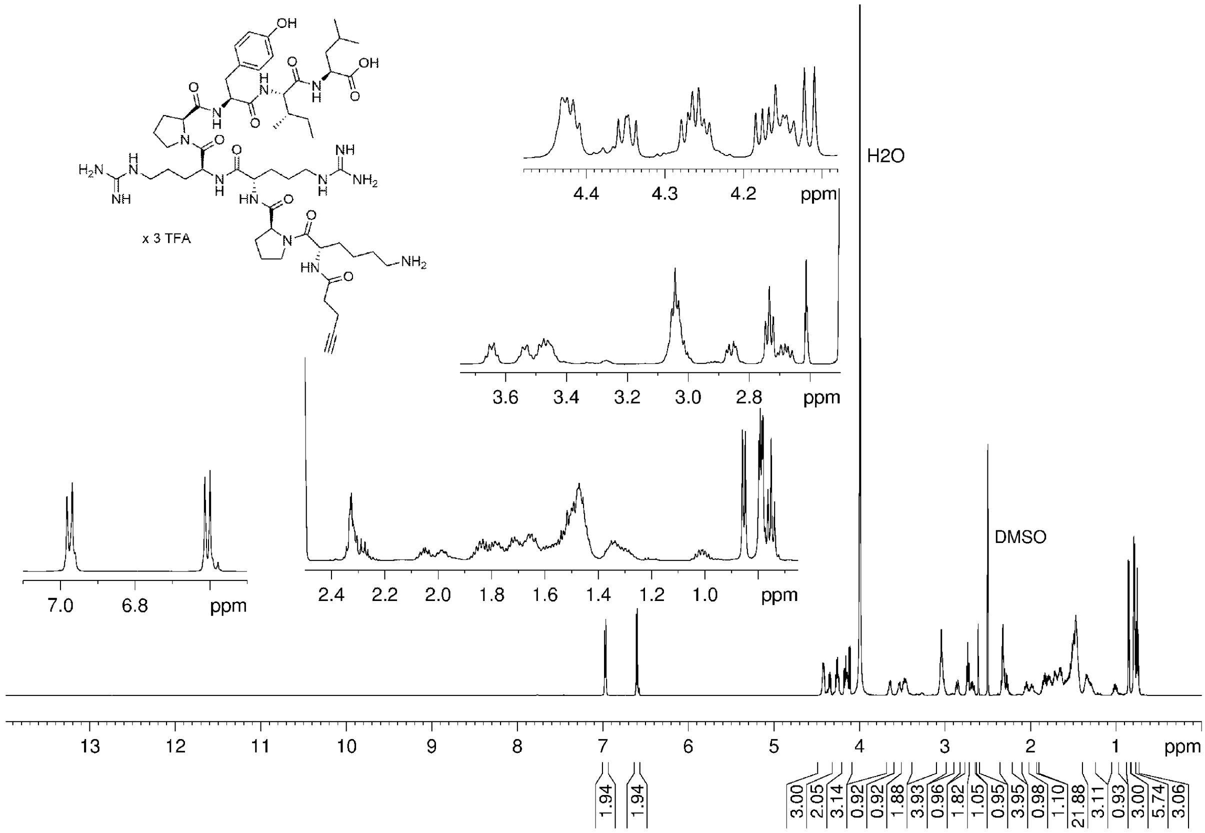

^{1}H-NMR spectrum (600 MHz, DMSO-d$_6$/D$_2$O 4:1 v/v) of compound **7**

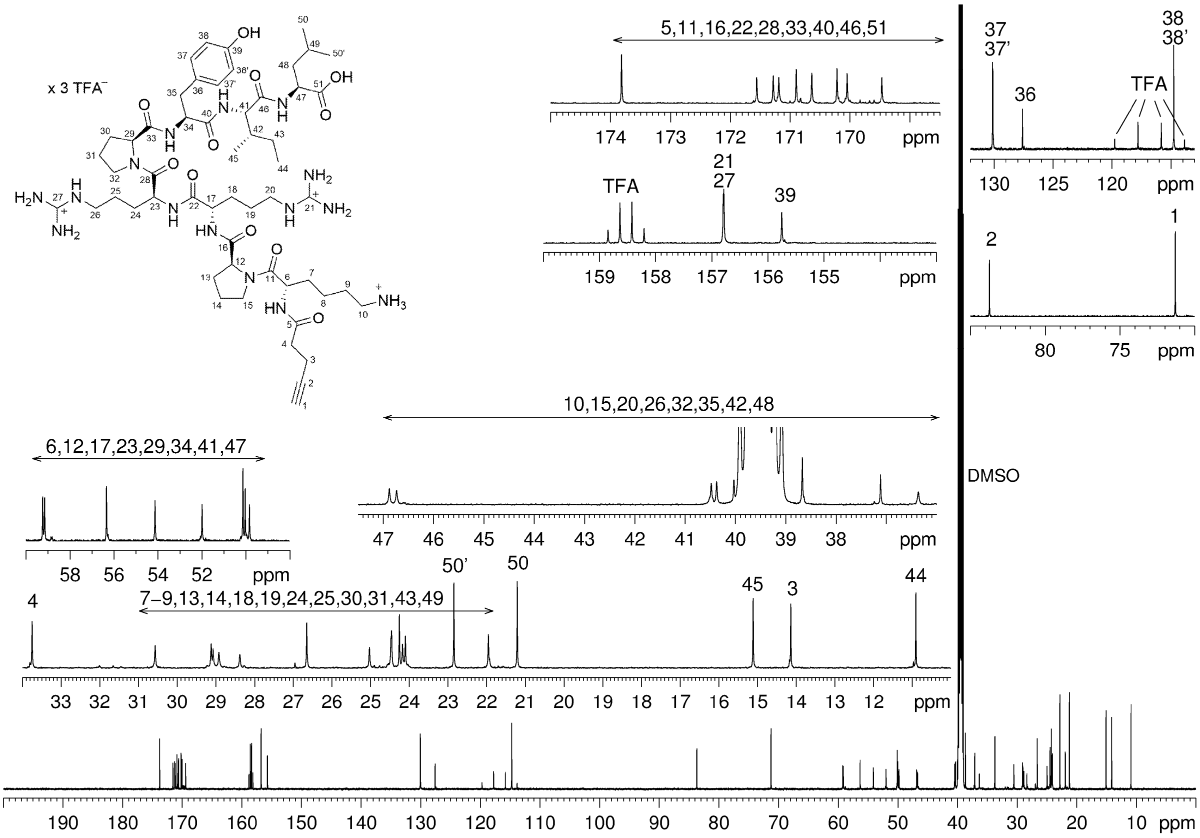

^{13}C-NMR spectrum (150 MHz, DMSO-d_6) of compound **7**

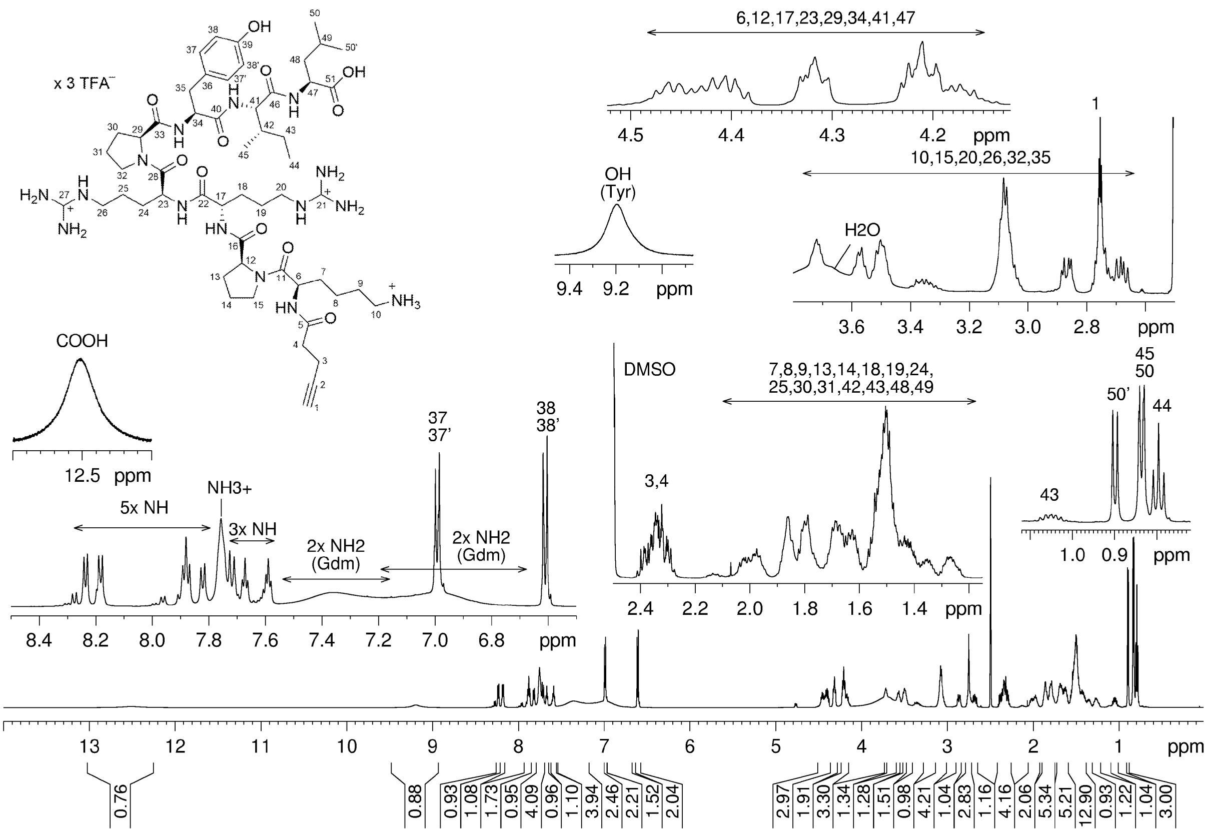

^{1}H-NMR spectrum (600 MHz, DMSO-d$_6$) of compound **8**

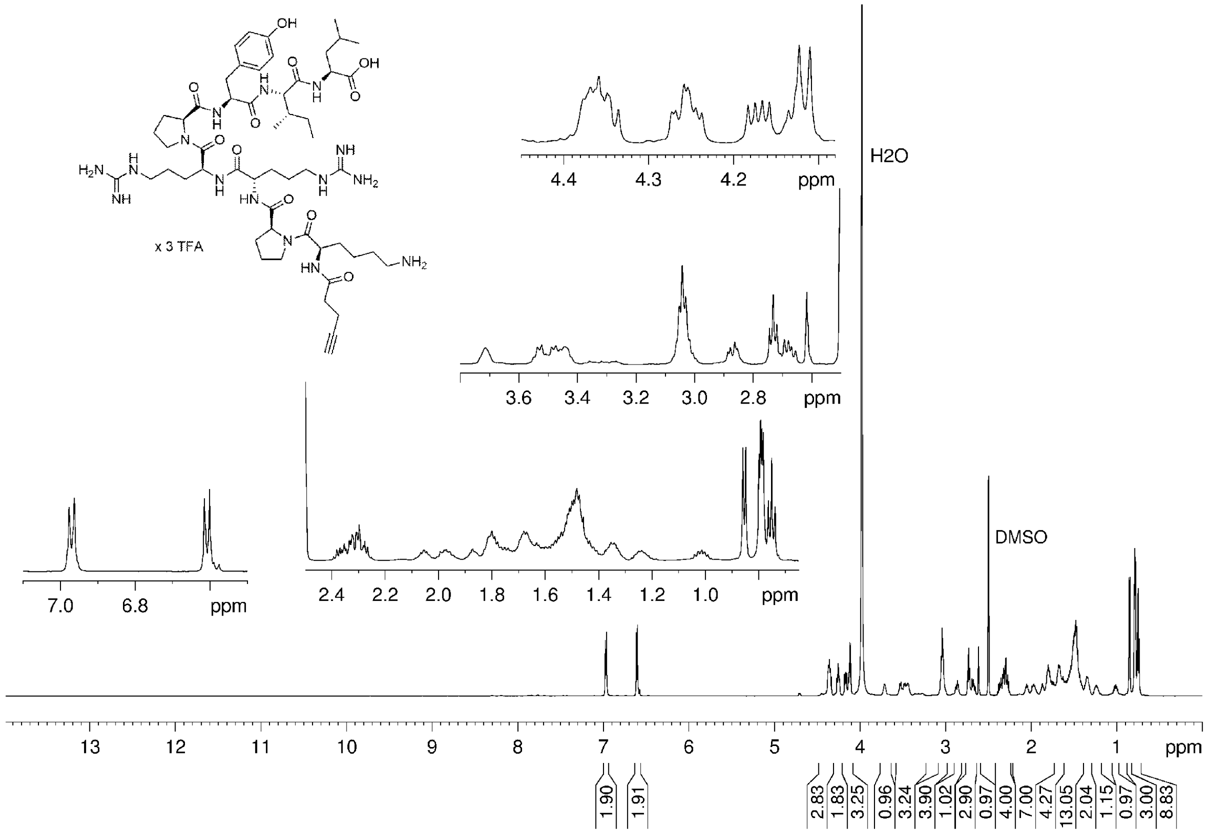

^{1}H-NMR spectrum (600 MHz, DMSO-d$_6$/D$_2$O 4:1 v/v) of compound **8**

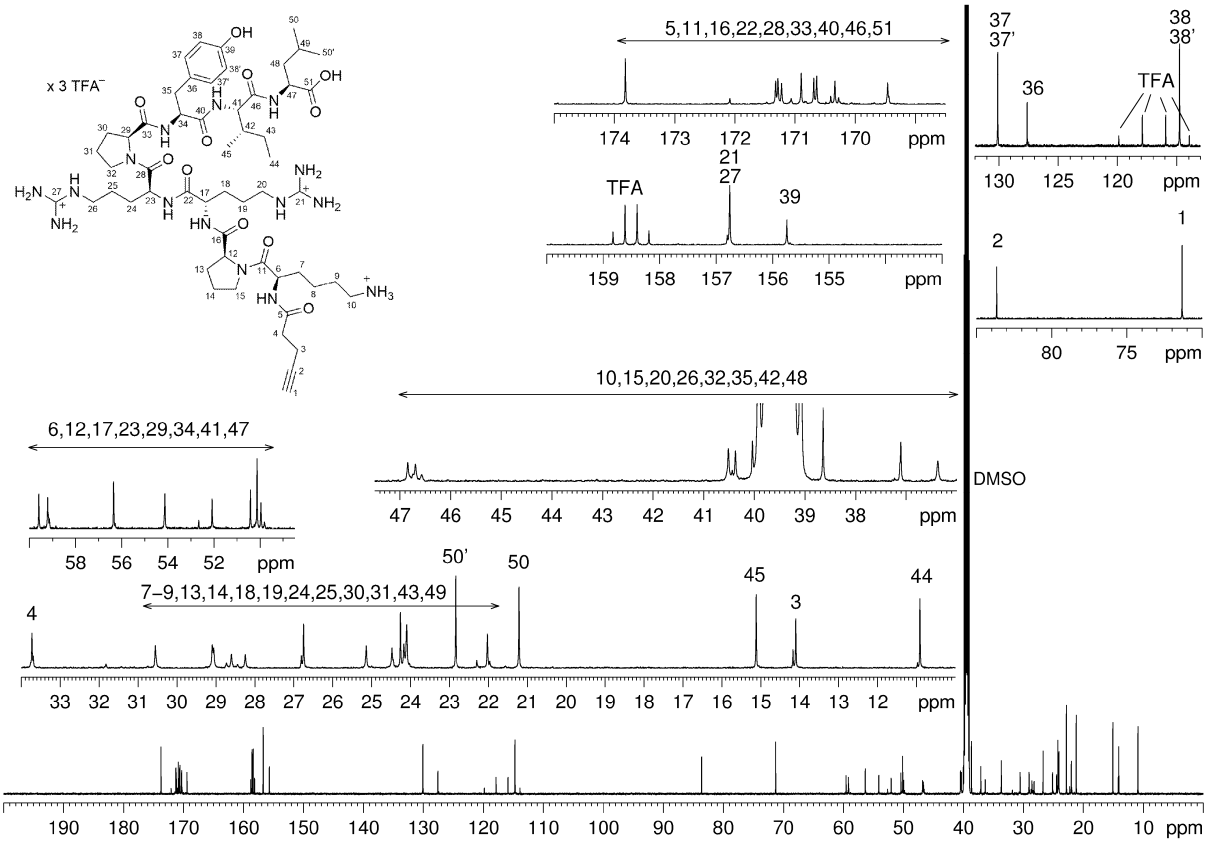

^{13}C-NMR spectrum (150 MHz, DMSO-d$_6$) of compound **8**

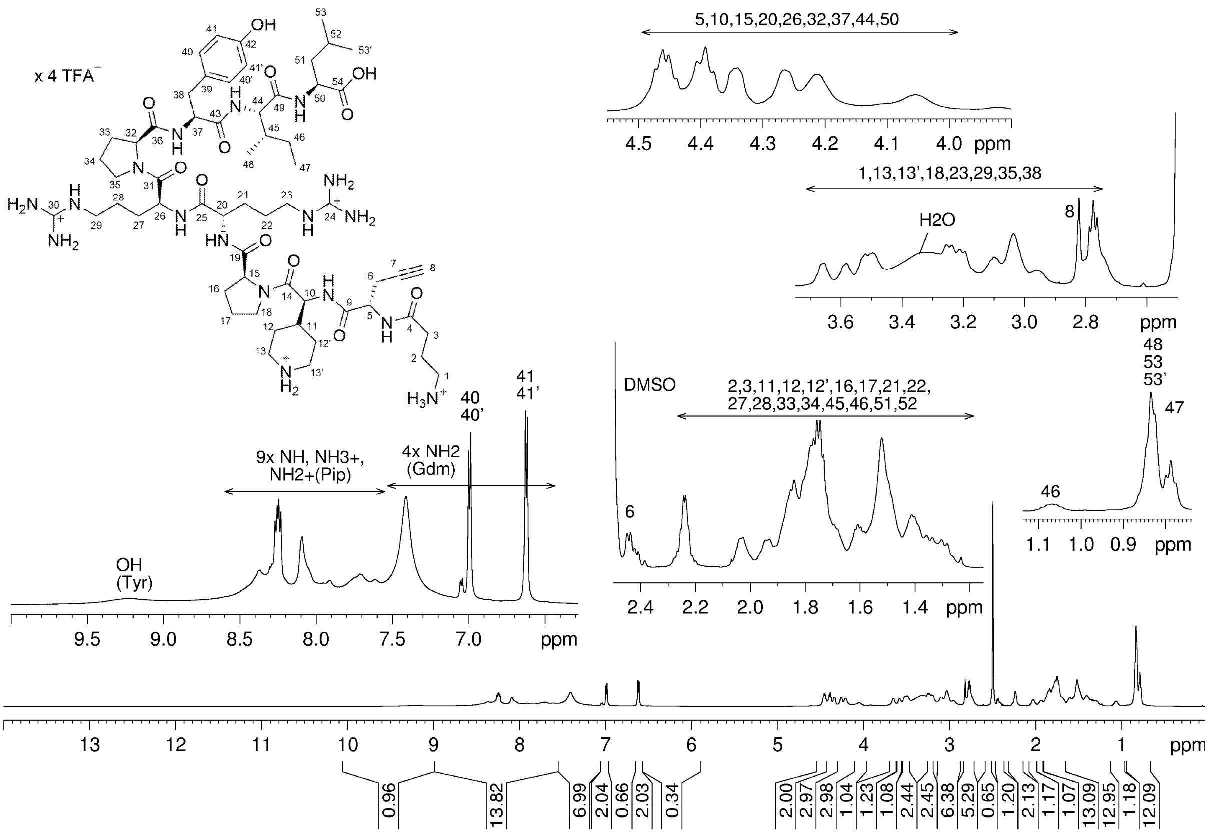

^{1}H-NMR spectrum (600 MHz, DMSO-d_6) of compound **9**

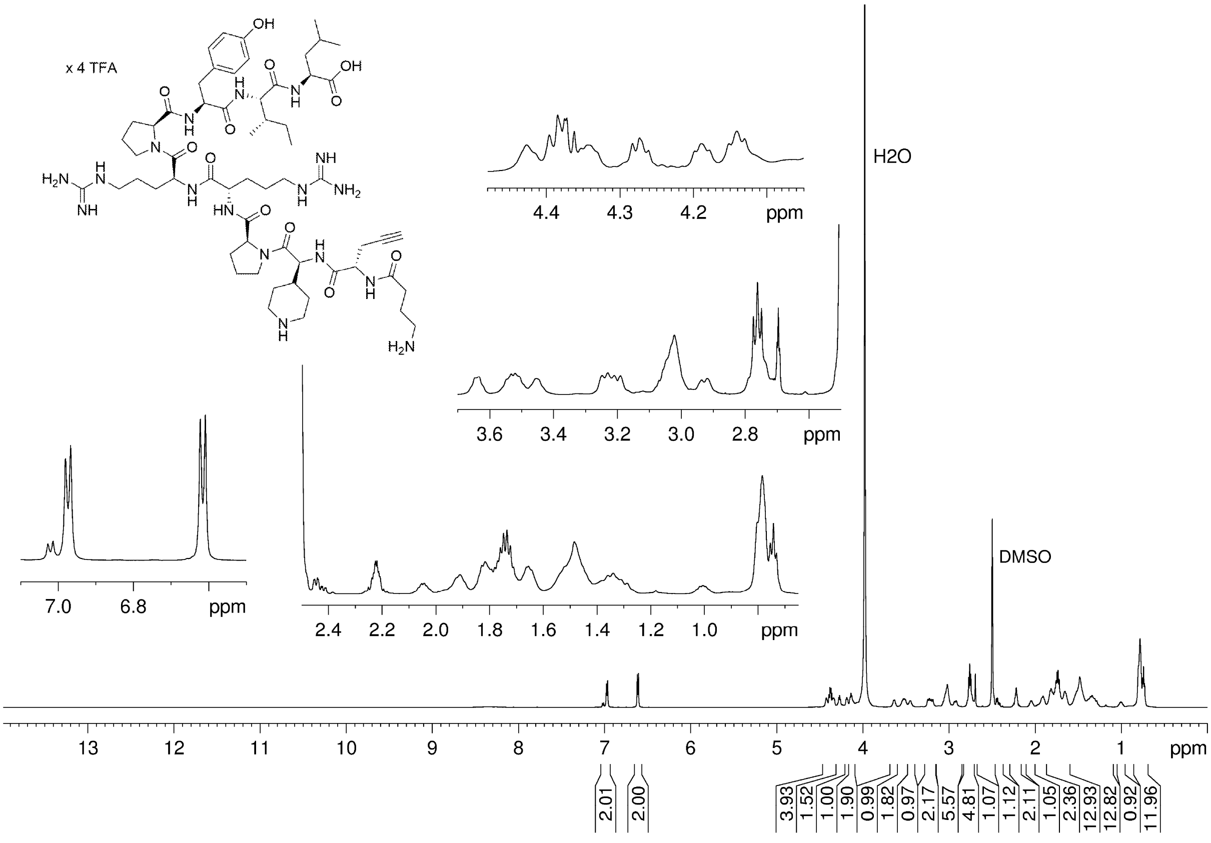

^{1}H-NMR spectrum (600 MHz, DMSO-d_6/D_2O 4:1 v/v) of compound **9**

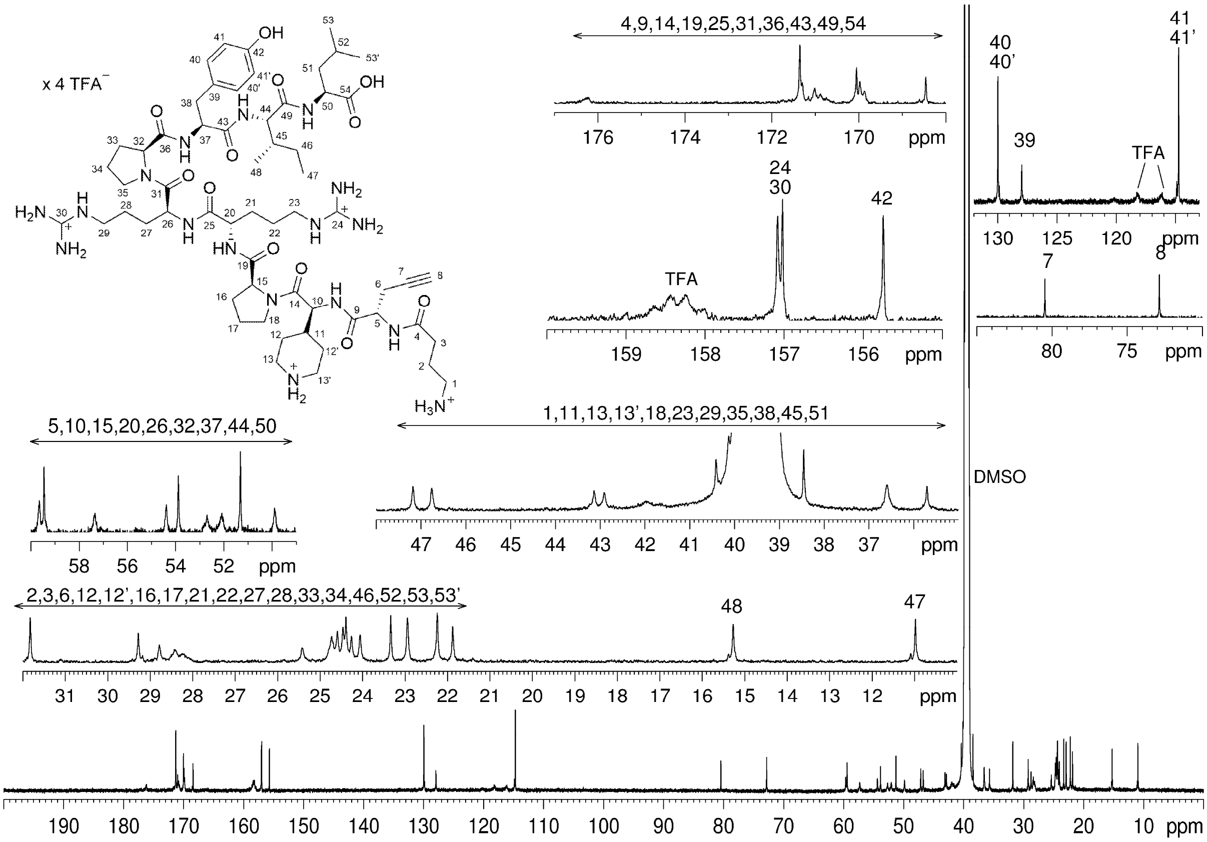

^{13}C-NMR spectrum (150 MHz, DMSO-d_6) of compound **9**

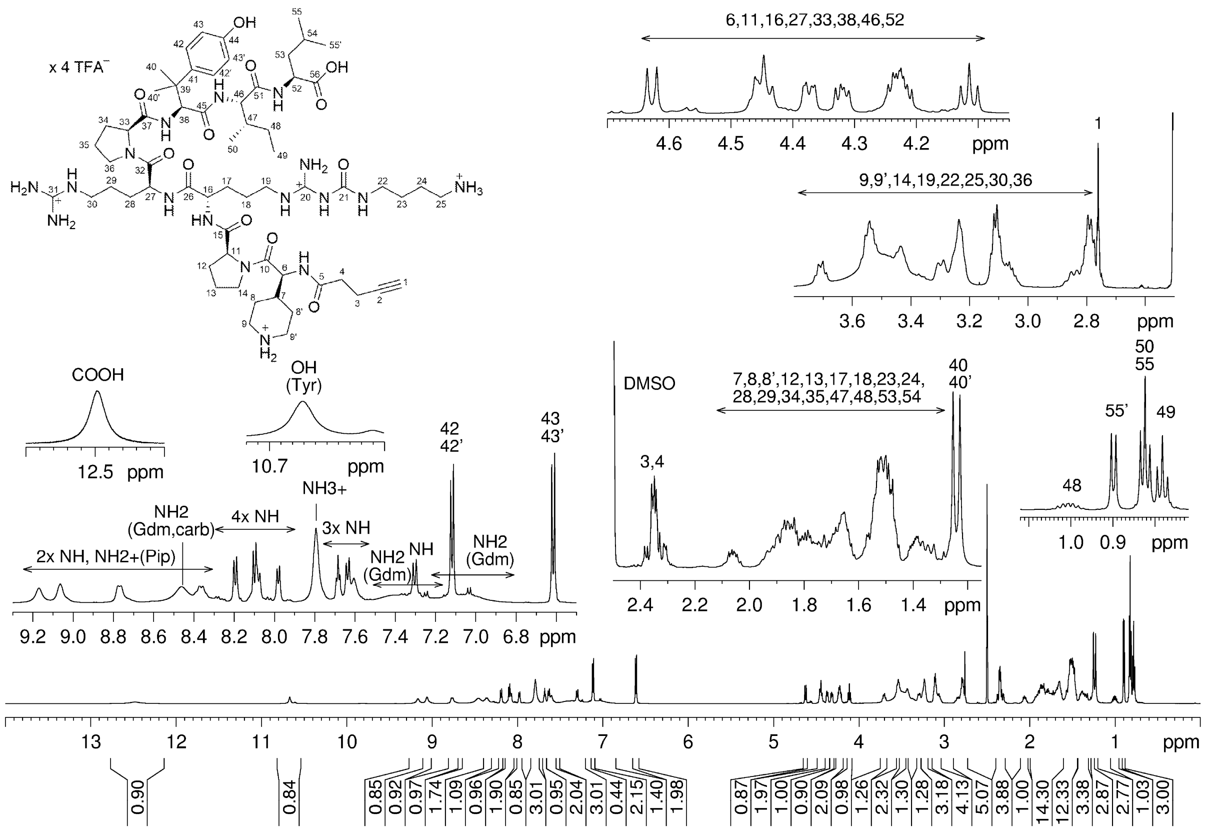

^{1}H-NMR spectrum (600 MHz, DMSO-d_6) of compound **10a**

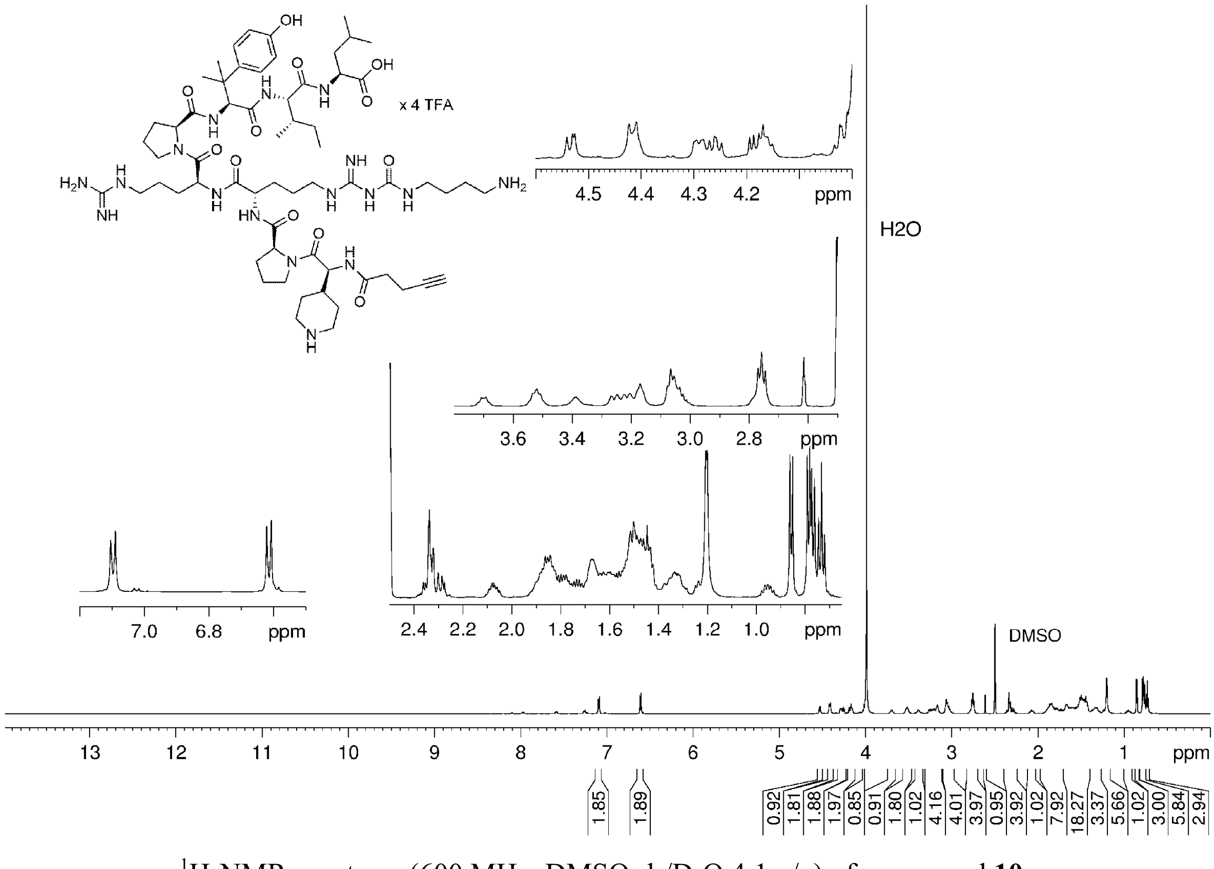

^{1}H-NMR spectrum (600 MHz, DMSO-d_6/D_2O 4:1 v/v) of compound **10a**

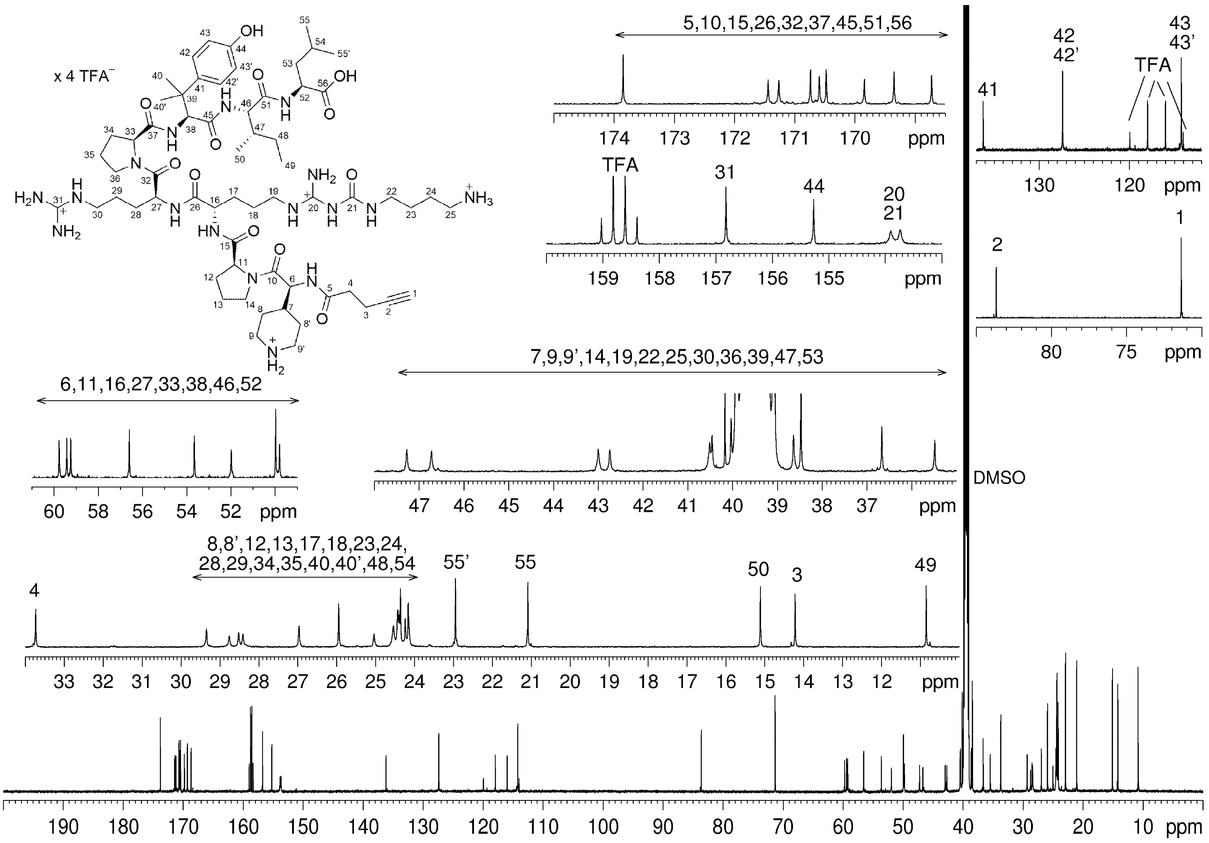

^{13}C-NMR spectrum (150 MHz, DMSO-d$_6$) of compound **10a**

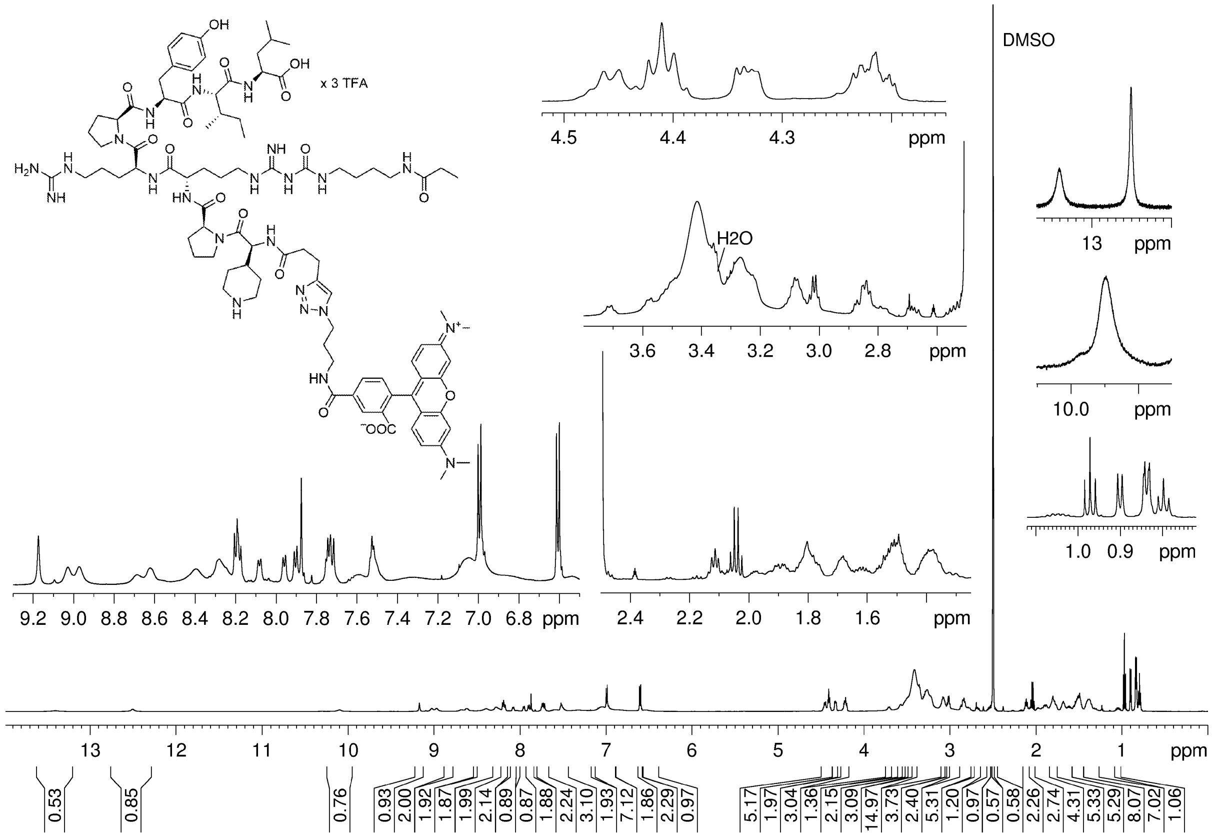

^{1}H-NMR spectrum (600 MHz, DMSO-d_6) of compound **13**

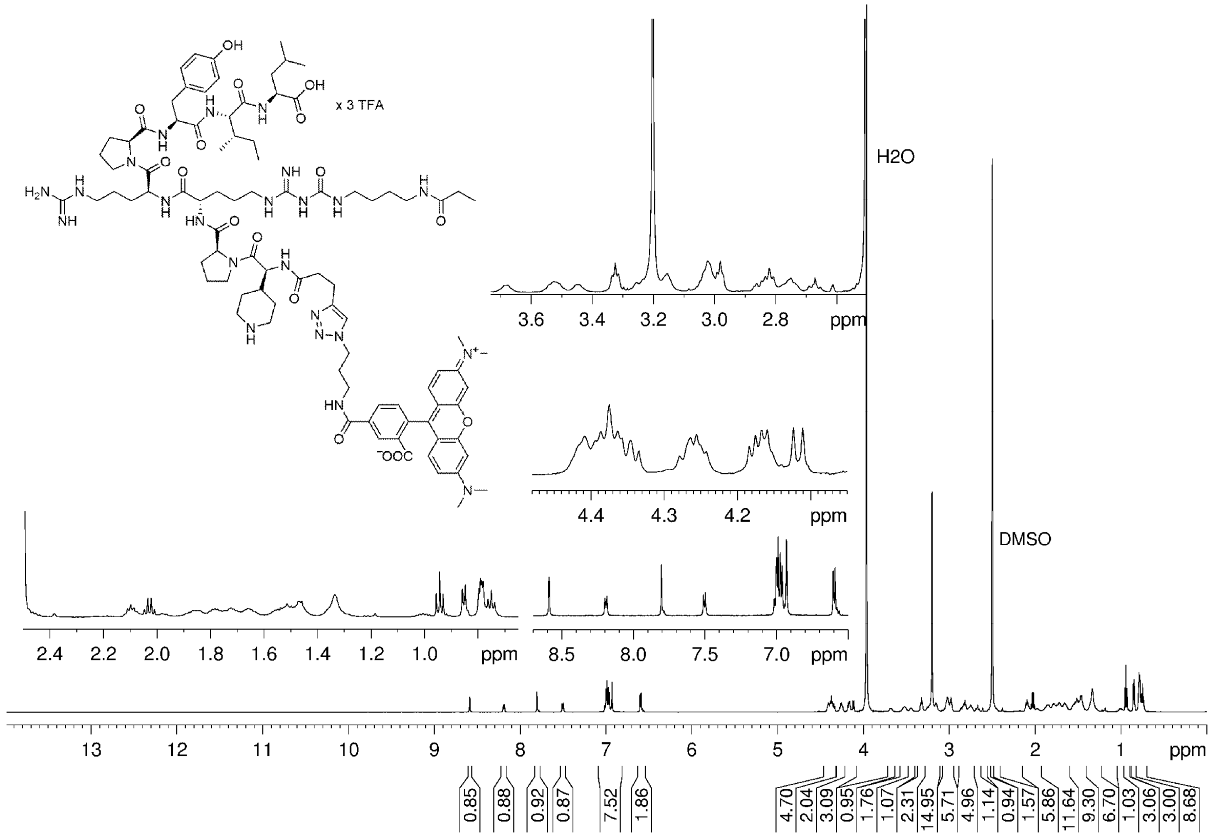

^{1}H-NMR spectrum (600 MHz, DMSO-d_6/D_2O 4:1 v/v) of compound **13**

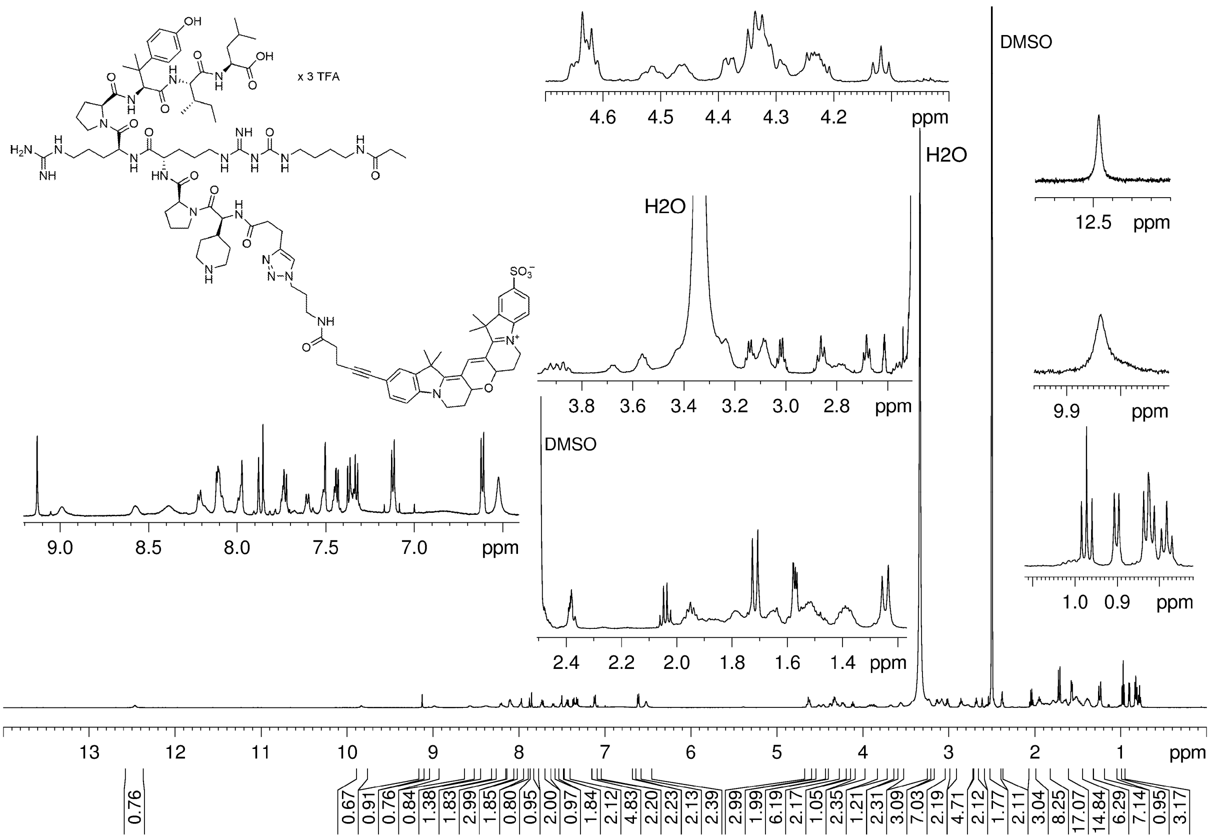

^{1}H-NMR spectrum (600 MHz, DMSO-d$_6$) of compound **18**

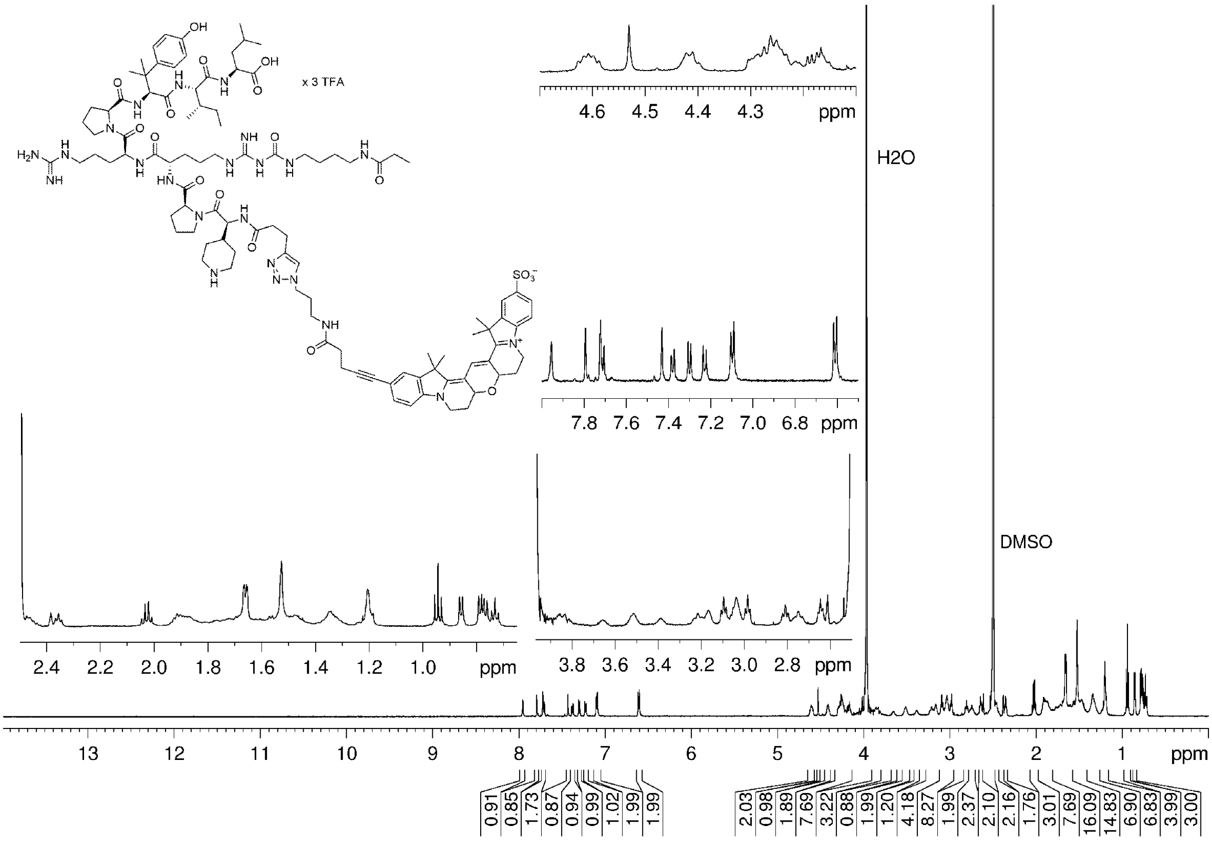

^{1}H-NMR spectrum (600 MHz, DMSO-d$_6$/D$_2$O 4:1 v/v) of compound **18**

6. References

(1) Schindler, L.; Moosbauer, J.; Schmidt, D.; Spruss, T.; Gratz, L.; Ludeke, S.; Hofheinz, F.; Meister, S.; Echtenacher, B.; Bernhardt, G.; Pietzsch, J.; Hellwig, D.; Keller, M. Development of a Neurotensin-Derived ^{68}Ga-Labeled PET Ligand with High In Vivo Stability for Imaging of NTS_1 Receptor-Expressing Tumors. *Cancers* **2022**, *14*, 4922.
